# Actions, recommendations, and public policy solutions for the management of dermatological diseases in Latin America: a systematic review

**DOI:** 10.3389/fpubh.2026.1929195

**Published:** 2026-10-19

**Authors:** Kelly Estrada-Orozco, Lina María Rincón-Martínez, Nathalie Ospina-Lizarazo, Jesús Utria-Munive, Elisabet Gutiérrez-Barbosa, María Camila Londoño-Restrepo, Genesis Andrea Bernier-Brito, Laura Marcela Garces-Perea, Lorena Mesa-Melgarejo, Lorena Herrera-Aranguren, Elin Xiomara Perea

**Affiliations:** 1 Hospital Universitario Centro Dermatológico Federico Lleras Acosta, Bogotá, Colombia; 2 Centro de Evidencia e Implementación (CEI-evidence), Bogotá, Colombia; 3 Faculty of medicine, Universidad El Bosque, Bogotá, Colombia

**Keywords:** dermatology, health policy, Latin America, skin diseases, systematic review

## Abstract

**Introduction:**

Dermatological diseases impose a substantial health burden and contribute to health inequalities across Latin America. However, data regarding regional public policies has not been systematically synthesized, limiting the identification of approaches, gaps, and best practices.

**Objective:**

To conduct a systematic review aimed at synthesizing the available literature and documents on public policy actions, recommendations, and solutions related to dermatological diseases in Latin America.

**Methods:**

Electronic databases and official government websites were searched. Documents published in Spanish, English, and Portuguese between 2015 and 2026 were included. When applicable, methodological quality was assessed according to study design. Data were narratively synthesized according to the identified groups of dermatological conditions. Programs or policies, implementation characteristics, and reported outcomes were described.

**Results:**

After screening 6,533 references identified through database searches, 74 scientific studies were included; additionally, the grey literature search identified 140 eligible documents. The analyzed documents on public policy solutions primarily focused on infectious diseases and neglected tropical diseases, particularly leprosy (15.4%) and leishmaniasis (11.2%), with most documents originating from Brazil (24.3%). The main actions identified included vaccination programs, early detection and transmission control strategies, strengthening of health surveillance and information systems, diagnostic decentralization, teledermatology, workforce training, implementation of guidelines and protocols, and community-based and intersectoral interventions. Implementation faced structural barriers such as territorial inequalities, limited problem-solving capacity at the primary care level, shortage of specialists, and weaknesses in health information systems. Implementation could potentially be facilitated through the integration of technological innovation, strengthening of primary care, community participation, and intersectoral approaches incorporating the social determinants of health.

**Conclusion:**

In Latin America, public policies in dermatology have primarily focused on disease control and reducing access gaps, with greater emphasis on infectious and neglected tropical diseases. The persistence of structural inequalities and the limited availability of data onchronic dermatological conditions highlight the need for more comprehensive, context-specific, and sustainable responses aimed at strengthening health systems and reducing inequities in dermatological care across the region.

**Systematic review registration:**

CRD420261322767 https://www.crd.york.ac.uk/PROSPERO/view/CRD420261322767

## 1 Introduction

Dermatological diseases (DDs) constitute one of the groups of non-fatal conditions with the greatest impact on global public health due to their high prevalence, chronic course, functional repercussions, and effects on mental health, productivity, and quality of life. Several analyses from the Global Burden of Disease study have shown that skin diseases, including dermatitis, acne vulgaris, psoriasis, and urticaria, are among the leading causes of disability worldwide, while conditions such as fungal skin diseases, dermatitis, acne vulgaris, and viral skin diseases are highly prevalent globally. These conditions represent a growing burden on healthcare systems, particularly in settings with limited access, delayed diagnosis, and shortages of specialists, and social and environmental inequities (1, 2).

In Latin America, contextual factors such as equatorial location, latitude, ultraviolet radiation exposure, and the distribution of skin phototypes may influence the epidemiological patterns of some of these conditions and shape health system responses (3–6). In addition, structural determinants such as socioeconomic inequalities, gaps in healthcare coverage, tropical and subtropical climatic conditions, and the persistence of infectious and neglected tropical diseases with cutaneous manifestations create a distinctive epidemiological landscape in the region (2, 3, 7, 8).

At the same time, the increasing prevalence of chronic inflammatory diseases, skin cancer, and disorders associated with population aging has generated new demands on Latin American healthcare systems (1, 4). These conditions require comprehensive responses that include health promotion, prevention strategies, epidemiological surveillance, access to diagnostic and therapeutic technologies, strengthening of primary healthcare, and reduction of territorial and population-based inequities (2). However, the analyzed documents suggest that institutional and regulatory responses to dermatological diseases are often fragmented across disease-specific programs, public health initiatives, and policies focused on insurance coverage and access to medications (9). Moreover, most available reviews have focused on clinical, epidemiological, or disease burden aspects, whereas information related to public policy, governance, models of care, health regulation, and intersectoral strategies remains poorly characterized (10, 11).

This gap limits the ability to identify successful experiences, recognize implementation gaps, and inform evidence-based decision-making processes for Latin American contexts, particularly in Colombia.

In this context, this study synthesizes the available data on public policy actions, recommendations, and solutions related to dermatological diseases in Latin America through a systematic review. Specifically, the review addresses the following question: What public policy actions, recommendations, and solutions for the management of dermatological diseases in Latin America have been reported?

## 2 Methods

A systematic literature review was conducted, and its reporting was structured in accordance with the PRISMA (Preferred Reporting Items for Systematic Reviews and Meta-Analyses) guidelines (12). The protocol was registered in PROSPERO (CRD420261322767). The literature search was performed in the Embase, MEDLINE, LILACS, Scopus, Web of Science, and Academic Search Complete databases. Additionally, a gray literature search was conducted using Google Scholar, official websites of Latin American ministries of health, and complementary snowball search strategies. Gray literature was included to capture relevant policy and implementation documents not indexed in bibliographic databases. To ensure a systematic and consistent selection process, predefined source-specific eligibility criteria were applied.

The search strategy combined controlled vocabulary terms according to each database (MeSH, DeCS, and Emtree) and free-text terms related to dermatological diseases, public policies, healthcare systems, and Latin America, including specific countries in the region. Search strategies were adapted to the characteristics of each information source (Supplementary Material 1).

Secondary studies, observational studies, and reports or evaluations of policies, programs, or interventions implemented in Latin American countries were included if they targeted the general population or subpopulations affected by the dermatological or cutaneous diseases defined in the study (Supplementary Material 2). Documents describing actions, strategies, programs, recommendations, or public policy solutions implemented since 2010 were considered, including national and subnational plans or programs, models of care (care pathways, integrated healthcare networks, and teledermatology), regulations, clinical practice guidelines, national-level recommendations and protocols, public health surveillance systems, health information systems, and access policies related to financing, insurance coverage, supply, and centralized procurement of technologies and medications.

Documents published in Spanish, English, and Portuguese between 2015 and 2026 were included, as well as regulatory documents or implementation tools published since 2010. The analysis was limited to policies, strategies, and programs implemented since 2010 in order to capture the transitional period preceding the adoption of the Sustainable Development Goals (SDGs) in 2015. During this decade, health systems began to progressively align with the principles that would later shape the 2030 Agenda. This temporal criterion makes it possible to include both initiatives developed in preparation for the post-2015 global framework and those implemented within the context of the SDGs, thereby facilitating an up-to-date analysis of recent transformations in the organization and delivery of health services across the region (13, 14). Individual clinical studies without implications for public policy decision-making or healthcare systems were excluded.

Two reviewers independently screened titles and abstracts using the Rayyan^©^ platform and subsequently assessed the full texts of potentially eligible studies. Discrepancies were resolved by consensus or through discussion with a third reviewer.

Data extraction was conducted by one reviewer using a standardized form specifically designed for the study. Information collected included author, year and country of publication, type of study or document, dermatological disease addressed, description of implemented actions or public policies, policy recommendations, implications for the healthcare system, implementation outcomes, barriers and facilitators, required resources, and limitations reported by the authors.

Risk of bias assessment was conducted by one evaluator using validated tools according to study design, including ROBIS for systematic reviews, CHEERS for economic evaluations, and the Joanna Briggs Institute (JBI) critical appraisal tools for observational and qualitative studies.

The narrative synthesis considered the methodological and conceptual heterogeneity of the included studies. Initially, a descriptive characterization of the publications was conducted according to study type, country, and health condition addressed. Subsequently, findings were organized into the following categories: infectious diseases with cutaneous manifestations (including the subcategories of parasitic, bacterial, viral, and fungal diseases, as well as documents addressing multiple infectious diseases), cutaneous neoplasms, primary dermatological diseases, systemic diseases with cutaneous manifestations, and multiple dermatological conditions. The synthesis was accompanied by summary tables including the main public policy approaches identified in the selected documents and their descriptions.

## 3 Results

The search of electronic databases identified a total of 6,533 references. After duplicate removal and title and abstract screening, 227 articles were selected for full-text assessment, of which 74 were ultimately included in the review.

Additionally, searches of institutional websites, Google Scholar, and snowballing strategies identified 140 relevant documents corresponding to regulatory documents and policy implementation tools, including clinical practice guidelines, recommendations, manuals, strategies, regulations, plans, and programs related to the dermatological conditions of interest (Figure 1). The list of included and excluded references is presented in Supplementary Material 3.

**Figure 1:**
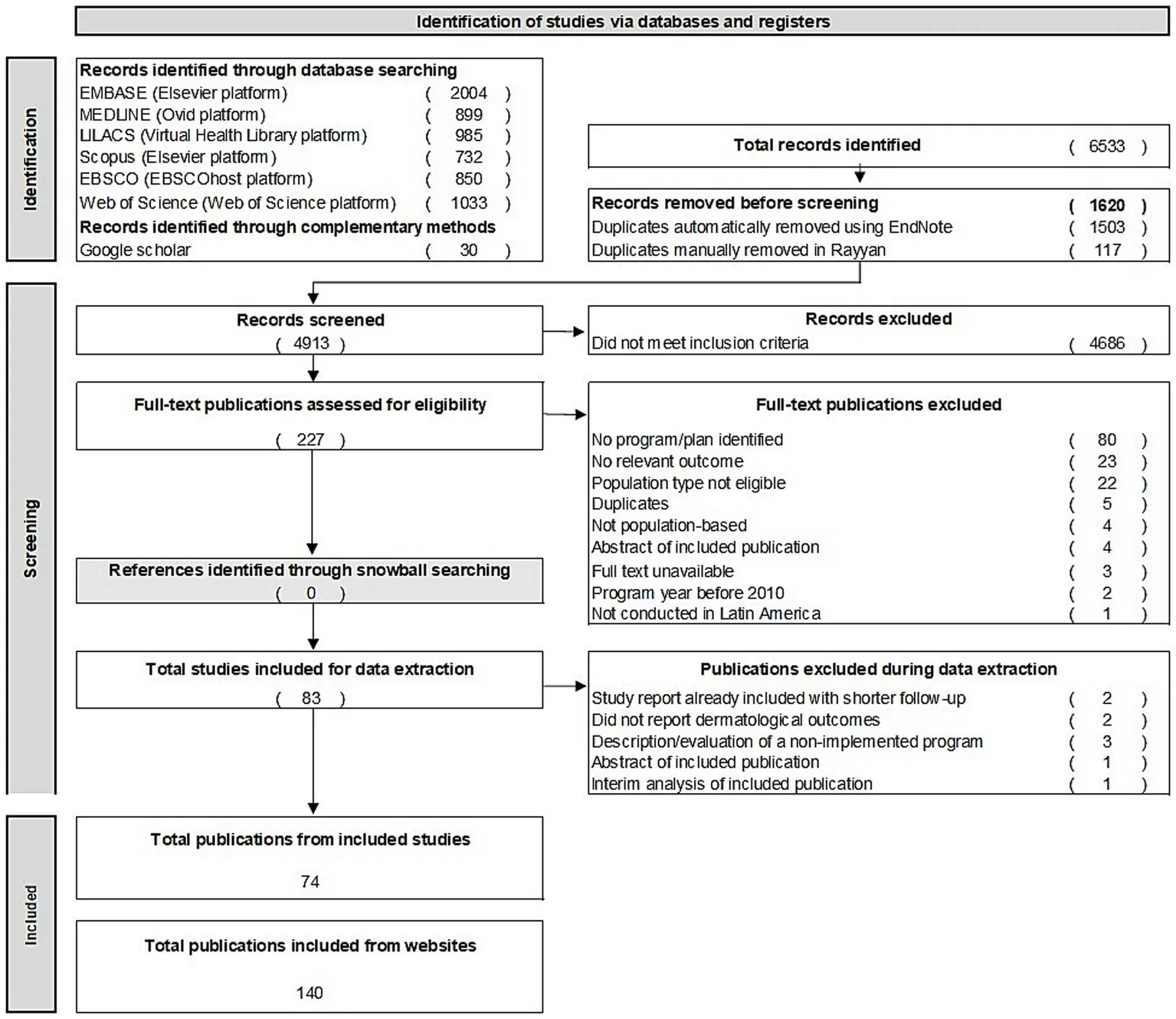
PRISMA flow diagram of the study selection process.

Among the 214 included documents, studies, tools, or policies from Brazil (24.3%), Argentina (9.3%), and El Salvador (9.3%) predominated, whereas no documents from Belize or Haiti were identified (Table 1). Regarding the dermatological conditions addressed, infectious diseases and conditions of public health interest were prominent, particularly leprosy (15.4%) and leishmaniasis (11.2%), as well as emerging conditions such as mpox (8.4%). In addition, documents addressing multiple dermatological conditions (13.1%) or several vaccine-preventable diseases (10.7%) were also identified.

**Table 1:** Frequency of dermatological conditions addressed in the included documents, by country.

Dermatological condition	AR	BO	BR	CH	CO	CR	CU	EC	ES	GU	GF	HO	MX	NI	PA	PY	PE	DR	UY	VE	LA	*n*	%
Acne						1																1	0.5
Skin cancer			1			1	1		1	2				1								7	3.3
Non-melanoma skin cancer (NMSC)			3		2							1										6	2.8
Atopic dermatitis	1		1		1																	3	1.4
Contact dermatitis	1																					1	0.5
Scabies					1							1										2	0.9
Systemic sclerosis			1																			1	0.5
Sporotrichosis			1																			1	0.5
Hidradenitis suppurativa			1																			1	0.5
Leishmaniasis	2	1	4		2	1			3	2			1	1	1	1	1			1	3	24	11.2
Leprosy	1		21		3	1		2	1				1		1	1	1					33	15.4
Systemic lupus erythematosus (SLE)			1	1				1		1						1			1			6	2.8
Cutaneous melanoma			1			1							1									3	1.4
Cutaneous myiasis					1			1			1			1								4	1.9
Mycetoma																1						1	0.5
Mpox		1	2		1	1		1	2			2	1		2	2	2		1			18	8.4
Onchocerciasis			1					2			1						2					6	2.8
Pemphigus vulgaris															1							1	0.5
Psoriasis	2		1							1		2	1									7	3.3
Actinic keratosis					1							1										2	0.9
Rickettsiosis	1											1										2	0.9
Measles	2	1					1			1					1	1						7	3.3
Tungiasis					1																	1	0.5
Various dermatological conditions	1	1	5	8				3	1	1				2	3			1		2		28	13.1
Various vaccine-preventable dermatological conditions	1		1	2	1	3		1	4	3		2	2		1	1	1					23	10.7
Varicella/Herpes zoster	6		5		1							1				2		1				16	7.5
Smallpox						1																1	0.5
HPV/Anogenital warts	2		2					3							1							8	3.7
Total	20	4	52	11	14	9	1	6	20	11	1	6	13	1	13	9	7	2	4	3	7	214	100
%	9.3	1.9	24.3	5.1	6.5	4.2	0.5	2.8	9.3	5.1	0.5	2.8	6.1	0.5	6.1	4.2	3.3	0.9	1.9	1.4	3.3		

AR, Argentina; BO, Bolivia; BR, Brazil; CH, Chile; CO, Colombia; CR, Costa Rica; CU, Cuba; EC, Ecuador; ES, El Salvador; GU, Guatemala; GF, French Guiana; HO, Honduras; MX, Mexico; NI, Nicaragua; PA, Panama; PY, Paraguay; PE, Peru; DR, Dominican Republic; UY, Uruguay; VE, Venezuela; LA, Latin America. SLE, Systemic lupus erythematosus; Mpox, Monkeypox; VDC, Various dermatological conditions; VPD-Derm, Various vaccine-preventable dermatological conditions; NMSC, Non-melanoma skin cancer; CD, Contact dermatitis; AD, Atopic dermatitis; SS, Systemic sclerosis; HS, Hidradenitis suppurativa; HZ, Herpes zoster; HPV, Human papillomavirus; AGW, Anogenital warts. The classification is based on the count of unique documents; the narrative synthesis integrates the contributions of each document according to the relevant thematic categories. Source: Prepared by the authors.

In terms of document type, clinical practice guidelines and protocols (25.2%) and recommendations/guidelines (19.2%) were highly prevalent, whereas among primary studies, cross-sectional studies were the most frequent (12.6%), as shown in Figure 2.

**Figure 2:**
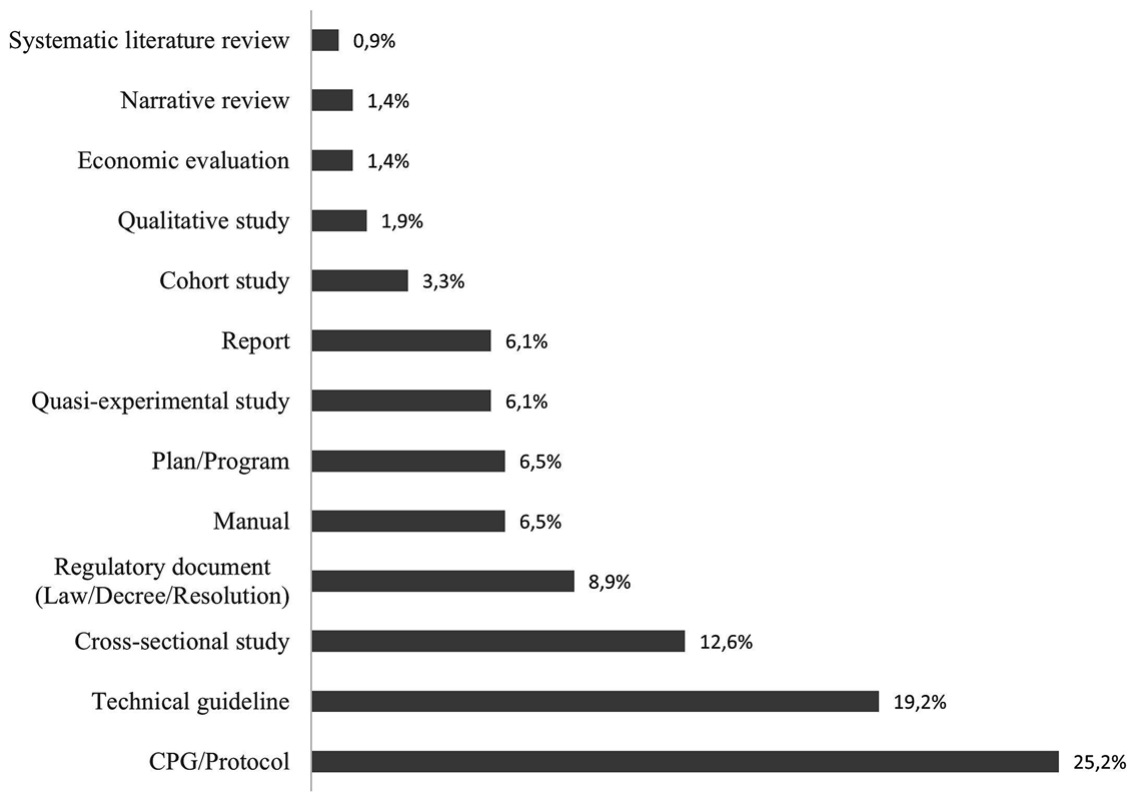
Distribution of included documents by type. Source: Prepared by the authors.

The methodological quality of studies reporting outcomes related to the implementation of public policy actions was generally variable, with moderate-to-high scores in most prevalence studies and moderate-to-low scores in most cross-sectional studies. The main strengths included the use of valid and standardized methods for measuring the conditions under study and, in several cases, the application of appropriate statistical analyses. However, common limitations were related to insufficient description of participant characteristics, potential selection and representativeness biases, the use of secondary records with possible underreporting or information loss, as well as the absence of identification and control of confounding factors. In addition, cross-sectional studies frequently showed the use of non-validated instruments, self-reported measurements, and primarily descriptive analyses, which limits causal inference and the generalizability of findings.

Overall, quasi-experimental and cohort studies showed moderate to high methodological quality, with notable strengths in the adequate measurement of outcomes and the use of appropriate statistical analyses, although frequent limitations included the absence of control groups, insufficient control of confounding, and reliance on secondary data. In qualitative studies, adequate methodological coherence was identified, although weaknesses were observed in reporting the theoretical framework and reflexivity. Systematic reviews presented an overall low risk of bias, whereas economic evaluations showed heterogeneous quality, with important differences in methodological reporting and the handling of uncertainty and costs. Complete information regarding the characteristics of each included document and risk of bias assessment (when applicable) is available in Supplementary Material 4.

The public policy actions, recommendations, and solutions identified for Latin America over the past 10 years are described according to the dermatological conditions addressed, classified into infectious diseases with cutaneous manifestations and infectious diseases in general, cutaneous neoplasms, diseases with primary dermatological involvement, systemic diseases with cutaneous manifestations, and dermatological diseases in general. Implementation contexts, as well as the most relevant challenges and achievements, are analyzed. A detailed description of the identified tools for addressing dermatological conditions and the main findings is presented in Supplementary Material 5.

### 3.1 Infectious diseases with cutaneous manifestations

A total of 159 documents (74.3%) reported public policy actions or solutions affecting infectious diseases with cutaneous manifestations.

#### 3.1.1 Parasitic diseases

A total of 37 documents (17.2%) related to parasitic diseases were identified, of which 72.7% focused on leishmaniasis, primarily originated from Brazil, Colombia, Guatemala, Mexico, and Venezuela.

##### 3.1.1.1 Leishmaniasis

Twenty-four documents were identified, mainly from countries in South and Central America, in addition to regional initiatives led by the Pan American Health Organization (PAHO). These documents predominantly consisted of manuals, protocols, programs, and observational studies aimed at strengthening surveillance, diagnosis, treatment, and vector control (15–38). The analyzed documents recognize leishmaniasis as a neglected tropical disease associated with poverty, precarious housing conditions, deforestation, migration, and limited access to healthcare services, disproportionately affecting rural and Indigenous populations, agricultural workers, children, and immunosuppressed individuals (20, 21, 24, 26–28, 30, 34). The main actions included mandatory surveillance systems (27, 28, 36, 38), strengthening of diagnostic networks (16, 23), comprehensive care guidelines, vector control through residual spraying and entomological surveillance (27, 29, 30, 33, 37), and community education initiatives (22, 28, 37). At the regional level, the PAHO Action Plan 2023–2030 (22) and the SisLeish epidemiological surveillance system (15) were particularly notable. Reported outcomes included improvements in diagnostic coverage, surveillance, and community participation, highlighting a participatory strategy implemented among the Wajãpi Indigenous population in Brazil that reduced time to diagnosis and increased cure rates after the first treatment cycle (16, 18). The main barriers included geographic challenges, underreporting, diagnostic limitations, and cultural barriers (15–18, 22, 26, 30–32, 34), whereas facilitators included international cooperation, regulatory frameworks, free treatment access, and community participation (15–18, 21–23, 28, 31, 37).

##### 3.1.1.2 Onchocerciasis

Six studies conducted primarily in Brazil, Venezuela, Guatemala, and Mexico focused on elimination programs based on mass ivermectin administration, epidemiological surveillance, and entomological and serological monitoring (39–44). These programs documented interruption of transmission and disease elimination in Guatemala and Mexico, with therapeutic coverage rates exceeding 85% and successful post-treatment surveillance (39, 44). In contrast, active transmission foci persisted in Amazonian regions of Brazil and Venezuela, associated with Indigenous communities and hard-to-reach territories (41–43). Reported outcomes included sustained reductions in parasitic burden and strengthening of community-based surveillance systems (39, 42–44). Key facilitators included community acceptance, international partnerships, and robust surveillance systems, whereas barriers included geographic dispersion, population mobility, and logistical and sociopolitical challenges in border areas (41–43).

##### 3.1.1.3 Scabies

One national guideline in Colombia and one clinical guideline in Mexico were identified, both aimed at strengthening the comprehensive management of scabies at the primary care level, particularly among vulnerable populations (45, 46). Actions included health promotion, prevention, diagnosis, treatment, epidemiological surveillance, active case finding, and the use of tools such as teledermatology and digital microscopy (45). The main barriers were diagnostic limitations, difficulties in accessing treatment, and structural conditions such as overcrowding, whereas facilitators included treatment availability, staff training, and regulatory support (45, 46).

##### 3.1.1.4 Tungiasis

For tungiasis, the Colombian National Prevention and Control Plan was identified, targeting vulnerable rural populations under a comprehensive “One Health” approach integrating interventions involving humans, animals, and the environment (47). The program implemented community surveillance, health education, free treatment with dimeticone, and interventions addressing environmental determinants through outreach teams and territorial authorities. The main barriers included underreporting, limited access to care, and unsafe parasite extraction practices, whereas facilitators included support from national policies and international technical assistance. The objectives guiding these interventions included reducing morbidity, hyperinfestation, disability, and disease-related stigma (47).

##### 3.1.1.5 Myiasis

For myiasis, four regulatory documents and clinical guidelines from Costa Rica, El Salvador, Honduras, and Panama were analyzed (48–51), aimed at strengthening epidemiological surveillance, standardizing diagnosis and treatment, and promoting community-based prevention. Actions included notification protocols, diagnostic and therapeutic algorithms, health education, and intersectoral coordination involving environmental sanitation and vector control measures (48–50).

These programs prioritized rural and vulnerable populations living in contexts of poverty and limited access to healthcare services, recognizing the role of social determinants in the persistence of the disease (48, 49). Although data on impact was limited, the main facilitators included the existence of technical guidelines and training of healthcare professionals involved in the management of myiasis, without specification of the level of care, whereas barriers included gaps in access to care and poor hygienic and sanitary conditions (49, 51).

#### 3.1.2 Bacterial diseases

Regarding parasitic infections with cutaneous manifestations, 35 documents (16.4%) were identified, primarily related to the management of leprosy in Brazil (60%).

##### 3.1.2.1 Leprosy

Thirty-three documents were identified, mainly from Brazil, Colombia, and Ecuador, as well as other Latin American countries (52–84), with a predominance of guidelines, protocols, technical standards, and national plans aimed at strengthening leprosy control and elimination programs (55, 60, 61). The analyzed documents recognize the disease as associated with poverty, rurality, and limited access to healthcare services, and describes strategies focused on standardizing diagnosis and treatment through multidrug therapy, strengthening epidemiological surveillance, mandatory notification, active case finding, contact tracing, and enhancement of health information systems and diagnostic networks (52, 54, 58–61). National programs with elimination targets by 2030 were also identified, particularly targeting rural and Indigenous communities, children under 15 years of age, and other vulnerable groups (58, 60). Evidence from Brazil also addressed social protection strategies, particularly the Bolsa Família Program, a conditional cash transfer program targeting vulnerable households. Four studies identified in a systematic review reported favorable associations between conditional cash transfers and leprosy treatment outcomes, while one reported mixed results (57, 64, 68). The main expected outcomes included early diagnosis, reduction of disability, and interruption of transmission (53, 54), whereas the primary barriers were delayed diagnosis, stigma, insufficient training at the primary care level, and territorial inequalities in diagnostic and therapeutic access (56, 59, 61). Key facilitators included the free availability of multidrug therapy, the existence of structured national programs, international support, and the progressive strengthening of surveillance systems and laboratory networks (54, 58, 60).

##### 3.1.2.2 Rickettsial diseases

Information on rickettsial diseases was derived from two clinical practice guidelines developed in Argentina and Panama, aimed at strengthening diagnosis, treatment, and epidemiological surveillance in response to underdiagnosis and the high clinical variability of these infections (85, 86). Actions included diagnostic protocols based on polymerase chain reaction (PCR) and immunofluorescence, early treatment with doxycycline even in the absence of laboratory confirmation, mandatory notification, strengthened epidemiological surveillance, and preventive measures against tick exposure (85). In Panama, unified clinical protocols were implemented in a context of high lethality, prioritizing early diagnosis and institutional coordination (86). The main barriers were diagnostic limitations and restricted laboratory access, whereas early detection and the standardization of clinical and epidemiological approaches were identified as the main facilitators for reducing mortality associated with these infections.

#### 3.1.3 Viral diseases

A total of 50 documents and studies (23.4%) addressed viral infections with dermatological manifestations, with 52.0% originating from Argentina, Brazil, and El Salvador.

##### 3.1.3.1 Measles

Seven documents were identified from Argentina, Bolivia, El Salvador, Honduras, Peru, and the Dominican Republic, mainly consisting of national guidelines and protocols aimed at strengthening epidemiological surveillance, vaccination, and rapid outbreak response (87–93). The documents identified declining vaccination coverage, accumulation of susceptible populations, population mobility, and diagnostic challenges in differentiating measles from other febrile exanthematous diseases as the main concerns (88, 90, 91). Implemented strategies included mandatory surveillance and notification systems, active case and contact tracing, routine and outbreak-response vaccination campaigns, vaccination coverage monitoring, and strengthening diagnostic and response capacity at the primary healthcare level, in addition to intersectoral actions in educational settings to promote vaccination and coordination between the health and education sectors (87–92). The main barriers identified were vaccination coverage gaps, epidemiological silence, and population mobility (90, 93), whereas the principal facilitators included free and mandatory vaccination policies, structured surveillance systems, rapid response teams, and support from specialized laboratories (90, 93). The documents emphasized that measles control depends on maintaining sustained vaccination coverage and sensitive surveillance systems with rapid response capacity to address viral reintroduction.

##### 3.1.3.2 Varicella

Sixteen documents and studies were identified from Panama, Colombia, Brazil, Argentina, and Uruguay, in addition to regional reviews for Latin America and the Caribbean, with a predominance of observational and ecological studies focused on the impact of national universal vaccination programs in response to the high burden of disease among pediatric populations (94–109). The main strategies included universal vaccination, progressive implementation of two-dose schedules, epidemiological surveillance, outbreak monitoring, and post-exposure prophylaxis. Reported outcomes showed consistent reductions in incidence, hospitalizations, and mortality across all evaluated countries: in Panama, hospitalizations decreased by up to 88.3% among children under 14 years of age (94); in Colombia, incidence decreased by 70.5% among children aged 1–4 years, along with reductions in mortality (95); and in Brazil, Argentina, and Uruguay, substantial reductions in hospitalizations, mortality, nosocomial outbreaks, and associated costs were documented (96–107). The main barriers included underreporting, passive surveillance systems, suboptimal vaccination coverage, and disruptions associated with the COVID-19 pandemic, whereas facilitators included national immunization programs, public financing, surveillance systems, and cold-chain infrastructure (95, 96, 100, 106, 108, 109).

##### 3.1.3.3 Smallpox

One regulatory document from El Salvador was identified, aimed at strengthening epidemiological surveillance in response to the risk of reemergence of vaccine-preventable diseases, including smallpox (110). The principal strategy involved the creation of an Integrated Sentinel Surveillance Management Team, using an intersectoral and health system-based approach to improve early detection, notification, and response capacity for epidemiological events. Although no quantitative outcomes, specific barriers, or facilitators were reported, the document emphasized the importance of institutional coordination, technical capacity, and operational sustainability as key elements for health risk management.

##### 3.1.3.4 HPV (human papillomavirus)

Eight documents and studies were identified from Brazil, Argentina, El Salvador, and Peru, including observational studies, technical guidelines, and national vaccination plans aimed at reducing the disease burden associated with anogenital warts, cervical intraepithelial neoplasia, and cervical cancer (111–118). The main actions included universal HPV vaccination, progressive expansion of eligible groups, simplification of vaccination schedules, strengthening of surveillance systems, and integration with screening and sexual health programs. Brazil expanded vaccination to boys and high-risk populations (112), Argentina incorporated two-dose schedules, vaccination for males, and later the nonavalent vaccine and a single-dose strategy (113, 114), whereas Peru implemented school-based and outreach vaccination strategies targeting individuals aged 9–18 years (118). Reported outcomes included significant reductions in hospitalizations due to anogenital warts and favorable vaccine safety profiles, including indirect effects in non-target populations (111, 112). The main barriers included budgetary constraints, low risk perception, family refusal, and difficulties in reaching populations outside the school system (111, 115, 117, 118), whereas facilitators included free vaccination, coordination with the educational sector, simplified vaccination schedules, and strengthened surveillance systems (113, 116–118).

##### 3.1.3.5 Mpox (Monkeypox)

Eighteen regulatory documents, guidelines, and national plans developed between 2022 and 2025 were identified from Brazil, Bolivia, Colombia, Costa Rica, Ecuador, El Salvador, Honduras, Mexico, Panama, Paraguay, Peru, and Uruguay, all aimed at responding to the mpox public health emergency (119–136). The main identified concerns were persistent transmission, introduction of cases into non-endemic countries, low population immunity, and inequities in access, particularly among key populations such as men who have sex with men, people living with HIV, and close contacts (119, 123, 129, 135).

The regional response was structured around four main axes: intensified epidemiological surveillance through mandatory notification, contact tracing, and strengthened PCR-based diagnostic capacity (120, 122–126, 128, 131, 133); clinical standardization for differential diagnosis, isolation, and symptomatic management (127, 129, 131, 136); prevention and control strategies through health education, risk communication, use of personal protective equipment (PPE), and targeted vaccination (124, 125, 128, 130, 132, 134); and comprehensive national preparedness and response plans involving intersectoral coordination and interventions at points of entry (119, 121, 134). Although normative information predominated, some studies reported relevant outcomes, including 4,264 confirmed cases among 9,473 notifications in Colombia (123), 36 confirmed cases in Costa Rica (128), and reductions in infection rates in territories with implemented policies in Brazil (121). The main barriers included limited diagnostic capacity, stigma, misinformation, and restricted access to vaccines and treatments, whereas facilitators included existing regulatory frameworks, previously established surveillance systems, support from the World Health Organization (WHO) and PAHO, availability of PCR testing, and training of healthcare personnel (121, 123–125, 128–130, 133–136).

#### 3.1.4 Fungal diseases

One cross-sectional study from Brazil and one report from Mexico were identified related on fungal infections.

##### 3.1.4.1 Mycetoma

The evidence on mycetoma consisted of a community dermatology program implemented in rural areas of Guerrero, Mexico, between 1991 and 2021, aimed at improving the diagnosis and management of this disease among vulnerable populations (137). The program combined training of local healthcare personnel, community education, periodic free healthcare campaigns, and teledermatology for remote consultation and follow-up, while also incorporating cultural adaptation and community participation. Reported outcomes included the identification of 115 cases, improvements in early detection, and reductions in complications associated with delayed diagnosis. The program remained operational for more than three decades due to institutional support, continuous training, and the use of telemedicine to expand coverage in remote areas (137).

##### 3.1.4.2 Sporotrichosis

One study conducted in Rio Grande do Sul, Brazil, was identified regarding the implementation of a referral service for the diagnosis and treatment of zoonotic sporotrichosis, aimed at reducing underdiagnosis and diagnostic delays associated with limited access to specialized services (138). The program integrated primary healthcare with a university hospital through referral protocols, mycological confirmation, mandatory notification, free itraconazole treatment, and periodic clinical follow-up. Reported outcomes included an increase in the average number of diagnosed cases from 3.25 to 11 cases per year and a reduction in time to diagnosis from 206 to 79.5 days. The main facilitators included institutional coordination, healthcare personnel training, and free access to treatment, whereas barriers included low clinical suspicion and diagnostic delays at the primary healthcare level (138).

#### 3.1.5 Multiple infectious diseases

Within the category of multiple infectious diseases, 35 documents and studies were identified from various Latin American countries and the Region of the Americas, predominantly consisting of guidelines, protocols, technical manuals, and national plans aimed at controlling vaccine-preventable diseases, neglected tropical diseases, and other emerging cutaneous infections (139–173). These documents primarily focused on addressing the persistent risk of transmission and outbreaks in contexts characterized by low vaccination coverage, population mobility, territorial inequities, and weaknesses in epidemiological surveillance systems.

The identified actions were grouped into four main areas: universal and targeted vaccination programs for measles-rubella, varicella, and HPV; strengthening epidemiological surveillance through active case finding, contact tracing, and reinforcement of laboratory capacity; integrated programs for neglected diseases such as leprosy and leishmaniasis with emphasis on early diagnosis and community-based surveillance; and strategies for clinical standardization and healthcare personnel training through guidelines and protocols (141, 142, 144, 145, 148–160, 162–173).

Reported outcomes included reductions in varicella cases and hospitalizations, maintenance of measles and rubella elimination in some countries, and strengthening of regional surveillance and response capacity for outbreaks and imported cases (139, 146–148, 150). The main barriers included insufficient vaccination coverage, misinformation, poverty, stigma, programmatic fragmentation, and difficulties in accessing healthcare services in rural areas, whereas the most relevant facilitators included national immunization programs, regulatory frameworks, public financing, international cooperation, and coordination with educational and community sectors (139, 140, 142, 143, 150, 151, 153, 154, 156–159, 164, 165, 167–169, 172).

### 3.2 Cutaneous neoplasms

A total of 18 studies and documents (8.4%) addressing the management of cutaneous neoplasms through public policy actions or solutions were identified, with the largest proportion originating from Brazil (27.8%)

#### 3.2.1 Non-melanoma skin Cancer (basal cell carcinoma and squamous cell carcinoma)

Eight documents addressing non-melanoma skin cancer were identified from Brazil and Colombia, focusing on early detection programs, strengthening of primary healthcare, and standardization of diagnostic and therapeutic approaches (174–181). Actions included screening campaigns, workforce training, development of clinical guidelines, and organizational models aimed at improving timely access to diagnosis in response to the increasing regional incidence of these conditions (175, 176, 178–181).

Among the most notable initiatives was the Brazilian National Skin Cancer Prevention Campaign (CNPCP), implemented since 1999 by the Brazilian Society of Dermatology, which improved the identification of suspicious lesions and enabled the detection of smaller basal cell carcinomas compared with patients outside the program (174, 176). The multicenter “TFD Brasil” program was also identified; this initiative was funded by the Brazilian government to expand access to photodynamic therapy through clinical training, provision of equipment, and implementation of standardized protocols, and was later extended to other Latin American countries (175). The program reported complete treatment response rates ranging from 71 to 86%, as well as strengthened technical capacity among participating centers.

The main barriers included infrastructure limitations, restricted access to biopsies, and shortages of specialized personnel, whereas clinical guidelines, workforce training, and regional cooperation acted as facilitators (176, 178–181).

#### 3.2.2 Melanoma

Four documents from Brazil, Costa Rica, and Mexico were identified, mainly consisting of clinical guidelines and recommendations aimed at strengthening early detection and timely referral programs for melanoma (180, 182–184). Actions focused on training primary healthcare personnel, standardizing diagnosis, and providing clinical follow-up for individuals with risk factors. The main barriers were delayed diagnosis and limited access to specialists and dermoscopy (180, 183), whereas the availability of protocols and strengthening of clinical capacities acted as facilitators (183).

#### 3.2.3 Actinic keratosis

Actinic keratosis was addressed in two clinical guidelines from Colombia and Mexico (185, 186), focused on prevention programs, early diagnosis, and standardized management at the primary healthcare level. Actions included workforce training, public education, and strengthening of diagnostic and therapeutic pathways. The main barriers were limited access to specialized care and geographic challenges, whereas clinical guidelines and capacity-building initiatives acted as facilitators.

#### 3.2.4 Skin cancer (not specified by histological type)

Six documents from Brazil, Cuba, El Salvador, Guatemala, and Panama addressed skin cancer in an aggregated manner, without differentiating between melanoma and non-melanoma skin cancer, focusing on comprehensive prevention and early detection programs (187–192). Actions included educational campaigns on photoprotection, strengthening of screening programs, and training of healthcare personnel, usually integrated into national cancer control programs (189–191).

Among the most notable initiatives was the school-based program “The Sun, Friend of Childhood” (“El Sol, Amigo de la Infancia”) (188), developed by the Brazilian Society of Dermatology, which combined teacher training, educational materials, and school-based activities to promote sun-protective behaviors. The program demonstrated improvements in knowledge and reductions in misconceptions regarding sun exposure. The main barriers included low adherence to photoprotection measures and limited access to specialized diagnosis (187, 190), whereas public campaigns, regulatory frameworks, and educational strategies acted as facilitators (187, 189, 190).

### 3.3 Primary dermatological conditions

A total of 14 documents (6.5%) related to public policies, actions, or strategies implemented for conditions with primary dermatological involvement were identified from nine Latin American countries.

#### 3.3.1 Acne

One clinical practice guideline from Ecuador, published in 2016, was identified for the diagnosis and treatment of acne. The guideline was directed toward multiple healthcare professionals and targeted neonatal, pediatric, adolescent, and adult populations. Its objective was to standardize and optimize care through recommendations on prevention, diagnosis, treatment, management of sequelae, and identification of psychosocial and emotional impacts associated with acne (193).

#### 3.3.2 Atopic dermatitis and contact dermatitis

The documents analyzed regarding atopic dermatitis and contact dermatitis included observational studies and clinical guidelines from Argentina, Colombia, Brazil, and Chile, primarily aimed at standardizing clinical management, strengthening primary healthcare, and recognizing occupational dermatitis as an occupational health issue (175, 192–195).

In Argentina, the “ECHO Atopic Dermatitis” tele-mentoring program was implemented, based on virtual training sessions, case discussions, and collaboration between specialists and general practitioners. The program involved 217 healthcare professionals and resulted in improvements in diagnostic and therapeutic knowledge, high participation rates, and satisfaction levels exceeding 98% (195). In Colombia, the implementation of stepwise international recommendations for atopic dermatitis was evaluated in 233 patients over a 24-month period. Interventions included education, skin care, topical therapies, phototherapy, and immunosuppressive treatment according to disease severity, resulting in reduced clinical severity (SCORAD reduction from 33 to 21), improved quality of life, and complete disease control in approximately one-third of patients (194).

The main barriers included training gaps, low adherence to guidelines, and unequal access to specialized care (194, 196, 197). Identified facilitators included standardized clinical protocols, continuous training, and teledermatology and tele-mentoring initiatives (177, 194, 195).

#### 3.3.3 Hidradenitis suppurativa

One national clinical protocol from Brazil was identified for the management of hidradenitis suppurativa (198), aimed at standardizing the diagnosis, treatment, and follow-up of this chronic and recurrent inflammatory disease, which is more common in women and typically begins during the second or third decade of life. The program established diagnostic criteria, therapeutic options, and regulatory and evaluation mechanisms within the healthcare system in order to improve comprehensive care and reduce clinical variability.

#### 3.3.4 Pemphigus vulgaris

One clinical practice guideline published in Mexico in 2013 was identified for the outpatient management of pemphigus vulgaris in adults, aimed at standardizing the diagnosis, treatment, and follow-up of this rare and potentially life-threatening autoimmune blistering disease (199). The guideline was directed toward dermatologists and family medicine physicians and included severity criteria, clinical follow-up recommendations, and selection of steroid-sparing adjuvant therapies according to patient characteristics. The proposed actions sought to reduce morbidity and mortality, minimize adverse effects associated with prolonged corticosteroid use, improve quality of life, and reduce the need for high-complexity therapies such as plasmapheresis and immunosuppressive agents (199).

#### 3.3.5 Psoriasis

For psoriasis, two cross-sectional studies from Argentina and regulatory documents from Brazil, Mexico, Panama, and Paraguay were identified, aimed at strengthening comprehensive care, standardizing management, and expanding access to systemic and biologic therapies, recognizing the substantial clinical and psychosocial burden of the disease (200–205).

The main intervention identified was the ECHO Psoriasis Argentina program (2015), based on tele-mentoring and teleconsultations, which demonstrated improvements in clinical knowledge (85–89%) and therapeutic changes in nearly 70% of cases (204, 205). The main barriers included access to high-cost therapies and low treatment adherence, whereas facilitators included clinical guidelines, regulatory frameworks, and institutional coordination (201, 202, 206).

### 3.4 Systemic diseases with cutaneous manifestations

#### 3.4.1 Systemic lupus erythematosus (SLE)

Six documents on systemic lupus erythematosus (SLE) were identified from Brazil, Chile, Ecuador, Guatemala, the Dominican Republic, and other Latin American and Caribbean countries, aimed at standardizing diagnosis and treatment, strengthening care pathways, and expanding access to specialized therapies (207–212). The clinical practice guidelines and regulatory frameworks included diagnostic algorithms, referral criteria, and structured follow-up recommendations. In the Dominican Republic, the inclusion of SLE as a catastrophic disease was proposed to ensure financial coverage and access to high-cost medications (212).

A notable initiative was the digital program “Let’s Talk About Lupus” (“Hablemos de Lupus”), focused on self-care education through social media, videos, and websites. The program reached more than 18.5 million people and improved disease understanding among 97.1% of surveyed participants (207).

The main barriers included diagnostic challenges, high treatment costs, and inequities in access to specialized care, whereas facilitators included institutional support, multidisciplinary teams, and the use of digital educational platforms (207, 209–211).

#### 3.4.2 Systemic sclerosis

One clinical practice guideline developed in Brazil in 2022 was identified for the comprehensive management of systemic sclerosis (213), aimed at standardizing diagnosis, classification, pharmacological management, and clinical follow-up of patients with cutaneous and systemic manifestations. The guideline addressed issues related to diagnostic complexity, clinical variability, and challenges in early detection and referral, as well as the disease burden on quality of life and healthcare costs. It established diagnostic criteria, evaluation of cutaneous manifestations, subtype classification, and structured follow-up strategies, while facilitating access to specialized laboratory services, medications, and care provided by trained healthcare professionals (213).

#### 3.4.3 Multiple dermatological conditions

Within the category of multiple dermatological conditions, 15 documents from Chile, Brazil, French Guiana, and Argentina were identified, mainly consisting of cross-sectional studies, programmatic reports, and protocols focused on reducing gaps in access to dermatological care in rural areas or regions with shortages of specialists through teledermatology, community programs, and strengthening referral pathways (214–227).

Teledermatology was the primary strategy, implemented since 2001 in French Guiana, 2008 in Brazil, and 2009 in Chile, integrating state telehealth networks with synchronous and asynchronous teleconsultations between primary healthcare and specialized dermatology services (214, 216–219, 222–224). In Brazil, this strategy also incorporated support from clinical protocols (216), whereas in Chile it was complemented by referral protocols (2015) and integrated network care protocols (2022) (222, 223, 226, 227). Reported outcomes included broad coverage, reductions in waiting times and referrals, decreases in unnecessary in-person consultations, diagnostic concordance rates exceeding 80%, and cost-effectiveness in Brazil (214, 216–219, 223, 224).

In Argentina, the Community Dermatology Program, implemented since 2009, expanded access in rural areas through mobile healthcare campaigns, clinical care, training, and educational activities, strengthening local capacities and student training, with subsequent replication in other provinces (220). In Brazil, the Specialized Pharmaceutical Care Component (CEAF), implemented in 2010, improved access to high-cost medications; however, legal claims persisted even for drugs included in official formularies, highlighting ongoing access gaps (221).

The main barriers included shortages of specialists, technological limitations, and high staff turnover, whereas facilitators included workforce training, coordination across levels of care, and strengthening of telehealth networks and community-based programs (214, 216–220, 222, 223).

### 3.5 Public policies associated with the included documents

As a complementary analysis, public policies and regulatory frameworks associated with the included documents were mapped in order to identify the legal and technical instruments supporting and guiding the actions, strategies, and recommendations previously described (Supplementary Material 6).

Overall, 71 public policies were identified, mainly corresponding to national regulatory frameworks, although regional initiatives promoted by PAHO and WHO were also identified, primarily aimed at the elimination of communicable diseases and neglected tropical diseases.

The identified policies addressed both specific dermatological diseases, such as leprosy, leishmaniasis, onchocerciasis, contact dermatitis, and HPV-related cancers, and broader public health frameworks related to vaccination, epidemiological surveillance, primary healthcare, and strengthening of healthcare systems. A marked concentration of strategies targeting the control and elimination of communicable and neglected tropical diseases was observed, particularly for leprosy and leishmaniasis, through approaches focused on early detection, epidemiological surveillance, active case finding, contact tracing, timely treatment, and strengthening of primary healthcare.

Regarding strategic approaches, most policies shared common components aimed at strengthening healthcare systems, integrating services, improving epidemiological surveillance, promoting equity, and addressing the social determinants of health. In addition, multiple documents emphasized community participation, intersectorality, and universal access as fundamental pillars for the implementation of sustainable interventions. These approaches were particularly evident in the regional communicable disease elimination plans promoted by PAHO/WHO.

Policies aimed at strengthening institutional and technological capacities were also identified, including telehealth strategies, digital health initiatives, diagnostic networks, and national surveillance systems. Brazil, Chile, and Colombia stood out for incorporating technological integration tools and models of care based on primary healthcare and integrated service delivery networks. Additionally, several countries included regulatory mechanisms related to quality of care, accreditation, health technology management, and access to essential medicines.

In the field of immunization and primary prevention, multiple laws, programs, and national vaccination strategies targeting vaccine-preventable diseases and cancers associated with viral infections, particularly HPV, were identified. These policies shared principles of free access, universality, equity, and financial sustainability, as well as population coverage targets and strengthening of surveillance systems.

## 4 Discussion

Addressing dermatological diseases requires understanding not only their growing burden on public health but also the responses formulated by governments and international organizations to address their main healthcare needs. These conditions represent a significant public health issue due to their high prevalence, chronic course, and impact on quality of life, in addition to their substantial contribution to the global burden of disability (1, 2). In Latin America, their impact is shaped by geographic, environmental, and social determinants, as well as by the persistence of infectious cutaneous diseases and the increasing prevalence of chronic conditions (3), making it particularly relevant to systematically analyze the strategies, programs, and public policy frameworks developed in the region for their prevention, diagnosis, and management.

The findings of this systematic review demonstrate that the response to dermatological diseases in Latin America has been primarily structured through technical and regulatory instruments, national plans, and programmatic strategies aimed at strengthening epidemiological surveillance, standardizing care, and expanding access to diagnosis and treatment. This approach reflects a prioritization of health system organization and institutional response, particularly in contexts characterized by territorial inequalities and social determinants that influence both the distribution of these conditions, exposure risks, and access to healthcare services.

However, the available information suggests that challenges persist regarding effective implementation, equity in access, and the generation of evidence to evaluate the impact of these policies on health outcomes.

Although the results are presented according to dermatological condition, the integrated analysis of the included studies allows the identification of common elements across interventions and public policy approaches that may be applicable to multiple dermatological conditions in similar contexts; therefore, these findings are discussed according to the challenges they seek to address.

### 4.1 High disease burden and persistent transmission

The analyzed documents show that policy actions in the region are primarily concentrated on infectious diseases, particularly neglected tropical diseases with cutaneous involvement, reflecting their high epidemiological burden and concentration among vulnerable populations. These diseases exhibit co-endemicity and persistent transmission in specific territories (228, 229).

Predominant strategies such as active case finding, contact tracing, and mass drug administration correspond to classic disease control approaches; however, when implemented in isolation, they are insufficient to sustainably interrupt transmission (229). In this context, the integration of interventions emerges as a key strategy, enabling multiple diseases to be addressed simultaneously, improving resource efficiency, and expanding public health impact (228). Nevertheless, implementation requires overcoming challenges related to protocol standardization, strengthening diagnostic capacities, and ensuring sustainable financing (229).

These findings also suggest that the persistence of the burden of dermatological diseases, both communicable and noncommunicable, is deeply linked to structural weaknesses within healthcare systems, reinforcing the need for comprehensive approaches.

### 4.2 Barriers to access to diagnosis and dermatological care

The findings highlight that barriers to access to dermatological care in Latin America are structural and persistent, linked to the unequal distribution of specialists, limitations in diagnostic infrastructure, and fragmentation of healthcare systems. These findings are consistent with the international literature, which emphasizes the urban concentration of dermatologists, gaps in primary healthcare, and the influence of social determinants on effective access to dermatological services (2, 9, 230). Public policies should therefore aim not only to expand the supply of services but also to reorganize models of care under integrated, territorial, and primary healthcare-centered approaches tailored to local contexts.

Teledermatology has emerged as one of the most relevant and consolidated strategies in the region, particularly in Chile, Brazil, and French Guiana, demonstrating broad coverage, reductions in waiting and referral times, decreases in unnecessary in-person consultations, and high diagnostic concordance among dermatologists (214, 216–218, 223, 224, 231). Its integration into national telehealth systems demonstrates the capacity of governments to incorporate technological innovation in response to territorial inequities and barriers to access (212, 214, 215). However, its implementation and long-term sustainability remain constrained by persistent digital divides, including unequal access to reliable internet connectivity, digital technologies, and technological literacy, particularly in rural and underserved populations. In addition, regulatory challenges, infrastructure and maintenance costs, and the need for continuous workforce training may limit scalability and sustained implementation. Addressing these barriers requires robust regulatory frameworks, continued investment in digital infrastructure, strategies to promote digital inclusion, and stronger integration with primary healthcare services (214, 216, 217, 219, 224, 231–233).

Similarly, community-based and territorial programs developed in countries such as Mexico and Argentina demonstrated promising results by combining community education, training of local personnel, clinical outreach campaigns, and telemedicine (228, 234). These experiences suggest that culturally adapted models integrated with local communities can improve early detection, expand access, and strengthen local capacities, particularly among rural and vulnerable populations. Likewise, diagnostic and therapeutic decentralization programs such as the Family Health Strategy in Brazil, and decentralized care models for diseases such as leprosy, demonstrate that strengthening primary healthcare can contribute to reducing disease burden and improving timely diagnosis (65, 70).

The identified plans and regulatory frameworks demonstrate important advances in the institutionalization of responses to dermatological diseases, particularly in Panama, Paraguay, and the Dominican Republic (203, 206, 212). These initiatives highlight the role of regulation in ensuring access, financing, and standardization of care.

These findings demonstrate important advances in institutional response capacity for dermatological diseases through specific policies, protocols, and programs; however, challenges related to sustainability, financing, territorial inequities, and impact evaluation persist. The analyzed documents suggest that the effective public policies are those integrating technological innovation, epidemiological surveillance, strengthening of diagnostic networks, community participation, and intercultural approaches, offering valuable lessons for building more equitable, responsive, and context-adapted healthcare systems for dermatological needs in the region.

### 4.3 Knowledge and training gaps

The findings demonstrate that knowledge and training gaps in dermatology represent a critical yet underaddressed component in the formulation of public policies, particularly in resource-limited settings, and are associated with the unequal distribution of specialists, barriers to access, and social inequities (2, 9, 230). Within this context, educational strategies should move beyond knowledge transfer and strengthen the problem-solving capacity of primary healthcare, reducing dependence on specialized care and aligning with integrated health system approaches (234, 235). Digital tools and tele-mentoring initiatives, such as Argentina’s Project ECHO and social media-based platforms, stand out due to their scalability, cost-effectiveness, and positive impact on knowledge acquisition and clinical decision-making, although their sustainability depends on infrastructure, protected time, and institutional support (195, 229, 236, 237).

In addition, community-based strategies, such as school programs and support groups, demonstrated meaningful effects on behaviors, adherence, and empowerment, underscoring the importance of integrating sociocultural components and community participation into public policies (228, 238), despite barriers such as stigma and limited institutionalization. Overall, the findings suggest that successful implementation requires comprehensive approaches combining training, technological innovation, strengthening of primary healthcare, and social participation. Experiences such as PACK in Brazil and academic exchange programs demonstrate that context-adapted standardization improves health system responsiveness (141, 215). Therefore, public policies should prioritize continuous training, knowledge networks, clinical guidelines, and investment in health information systems to ensure sustainable and equitable interventions (239–241).

### 4.4 Clinical variability and lack of standardization

The findings show that although clinical variability and lack of standardization continue to be identified as cross-cutting challenges in the management of dermatological diseases in Latin America, the development of clinical practice guidelines is not necessarily associated with disease burden. However, this situation may be partially explained by the difficulty in identifying local guidelines due to their lack of indexing in electronic databases and the challenges involved in locating them through manual searches on specific websites. In addition to reflecting inequities in the development and availability of standardization tools, this issue contributes to perpetuating differences in quality of care across contexts, ultimately affecting the quality of care, comparability of evidence, and the ability of healthcare systems to generate consistent responses (237).

Even more critically, the existence of clinical practice guidelines does not guarantee their implementation. It has been documented that up to 22% of healthcare professionals deviate from urticaria guideline recommendations due to reliance on individual clinical judgment, the perception that guidelines do not reflect the complexity of patients in real-world settings, and, particularly in Latin America, because guideline recommendations cannot always be implemented within local healthcare systems (242). It is therefore necessary to advance toward strategies for local adaptation of guidelines, strengthening of capacities, and mechanisms for monitoring adherence.

### 4.5 Social determinants of health

The findings of this review demonstrate that dermatological conditions cannot be understood or addressed in isolation from the social context in which they develop. Social determinants of health play a central role in shaping disease patterns, access to healthcare services, and dermatological health outcomes (2).

Cash transfer programs showed the most consistent results among the strategies described in this review, consistent with the evidence recognizing the central role of social determinants such as income, education, and access to services, and their relationship with health outcomes (2, 9, 243). In particular, low socioeconomic status is associated with poorer dermatological health outcomes through multiple interrelated mechanisms. In atopic dermatitis, psoriasis, and hidradenitis suppurativa, greater disease severity and poorer disease control have been documented among populations with lower socioeconomic status, which is associated with barriers to timely and continuous access to care (2).

Likewise, recognizing that social determinants encompass not only material conditions, social stigma emerges as a cross-cutting barrier affecting the entire continuum from healthcare-seeking behavior to the care process itself. Social and cultural dimensions such as discrimination, social exclusion, and community cohesion affect disease severity, quality of life, and mental health among affected populations (2, 243). It is therefore necessary to integrate these factors into the understanding of disease, recognizing the interaction between cutaneous conditions and social context (243), which implies moving toward comprehensive and intersectoral approaches that simultaneously address social, structural, and clinical factors.

### 4.6 Limitations and strengths

This review has limitations related to the heterogeneity of the evidence, study designs, conditions addressed, and outcomes evaluated. Searches conducted through ministry of health websites represented a challenge for the identification and retrieval of some documents; therefore, it is possible that documents published in local developer repositories or other sources were not identified.

The substantial heterogeneity across evidence sources, study designs, dermatological conditions, policy interventions, and reported outcomes limited direct comparisons and precluded quantitative synthesis; therefore, findings were synthesized narratively and should be interpreted considering this variability. In addition, eligibility was restricted to documents published in Spanish, English, and Portuguese, which may have resulted in the exclusion of relevant evidence published in other languages, although these languages capture a substantial proportion of the literature and policy documents produced in Latin America. Finally, data extraction was conducted by a single reviewer, which may have increased the risk of extraction errors or subjective interpretation compared with duplicate independent extraction.

The systematic conduct of the review, including prior publication of a protocol and the integration of diverse sources of information, constitutes one of the major strengths of the present study. This approach enabled the identification of relevant structural patterns and gaps.

Overall, the included documents demonstrated strengths in variable measurement and analytical approaches. Limitations related to applicability were identified, including incomplete characterization of participants, use of secondary data (underreporting, incomplete information, and possible misclassification errors), and insufficient control of confounding factors in analytical studies. In qualitative studies, weaknesses were observed in the explicit description of the theoretical framework and reflexivity. Taken together, the evidence presents moderate methodological quality due to the predominance of observational studies, the use of secondary data, and limitations in confounding control, which restrict causal inference. These considerations should be taken into account when interpreting the findings, particularly regarding their applicability.

## 5 Conclusion

Overall, the findings show that the identified public policy in dermatology across Latin America have primarily focused on infectious diseases and neglected tropical diseases, particularly leprosy and leishmaniasis, whereas chronic inflammatory diseases, autoimmune disorders, and other dermatological conditions have received less programmatic development and have more limited documentation available. The identified responses include vaccination programs, early detection and transmission control strategies, strengthening of surveillance and health information systems, diagnostic decentralization, teledermatology, workforce training, implementation of clinical guidelines, and community-based and intersectoral interventions, mainly aimed at reducing access gaps and controlling public health events.

The most relevant public policy solutions combine prevention, timely diagnosis, strengthening of primary healthcare, expansion of access to specialists, incorporation of technologies such as telemedicine, and community participation. However, structural barriers persist, including territorial inequalities, limited problem-solving capacity, shortages of specialists, and weaknesses in health information systems. On the other hand, implementation of interventions could be facilitated through the integration of technological innovation, strengthening of primary healthcare, community participation, and intersectoral approaches that incorporate the social determinants of health. The available information primarily originates from technical documents, guidelines, and national plans issued by ministries of health and international organizations, whereas the scientific literature is more limited and concentrated on observational studies and programmatic reports.

Taken together, these findings highlight persistent gaps in the scope and development of dermatological public policies in Latin America, particularly beyond infectious and neglected tropical diseases. Addressing these gaps requires strengthening primary healthcare, expanding equitable access to specialized services, reinforcing information and surveillance systems, and promoting territorial and intersectoral approaches responsive to the social determinants of health.

## Data Availability

The original contributions presented in the study are included in the article/Supplementary material, further inquiries can be directed to the corresponding author.
