# Supplementary material for: Actions, recommendations, and public policy solutions for the management of dermatological diseases in Latin America: a systematic review
Source: Front Public Health. 2026 Oct 19;14:1929195. doi: 10.3389/fpubh.2026.1929195 (PMC13724427; doi:10.3389/fpubh.2026.1929195)
Supplement: Supplementary file 1 [file Table_1.DOCX]

Supplementary Material

# 1 Supplementary Tables

**TABLE S1** Database and website search strategies

| **No.** | **Database:** | Medline | |
| --- | --- | --- | --- |
|  | **Search interface:** | Ovid | |
|  | **Search strategies** | **Records retrieved (n)** | **Search date** |
| #23 | #29 AND #43 AND #44 AND [medline]/lim AND [2015-2026]/py | 899 | 10/02/2026 |
| #22 | #6 AND #20 AND #21 AND [medline]/lim | 1485 | 10/02/2026 |
| #21 | #7 OR #8 OR #9 OR #10 OR #11 OR #12 OR #13 OR #14 OR #15 OR #16 | 3767548 | 10/02/2026 |
| #20 | #17 OR #18 OR #19 | 410705 | 10/02/2026 |
| #19 | mexico:ab,ti OR belize:ab,ti OR honduras:ab,ti OR 'el salvador':ab,ti OR nicaragua:ab,ti OR 'costa rica':ab,ti OR Panama:ab,ti OR cuba:ab,ti OR haiti:ab,ti OR 'dominican republic':ab,ti OR colombia:ab,ti OR venezuela:ab,ti OR ecuador:ab,ti OR peru:ab,ti OR bolivia:ab,ti OR chile:ab,ti OR argentina:ab,ti OR uruguay:ab,ti OR 'paraguay':ab,ti OR Brazil:ab,ti OR 'puerto rico':ab,ti OR guyana:ab,ti OR suriname:ab,ti OR guatemala:ab,ti | 387154 | 10/02/2026 |
| #18 | 'latin america':ab,ti | 31171 | 10/02/2026 |
| #17 | south AND 'central america'/exp | 10633 | 10/02/2026 |
| #16 | 'guideline*':ab,ti | 964821 | 10/02/2026 |
| #15 | 'recommendation*':ab,ti | 600379 | 10/02/2026 |
| #14 | 'strategic planning'/exp | 7967 | 10/02/2026 |
| #13 | 'program*':ab,ti | 1751174 | 10/02/2026 |
| #12 | 'health system*':ab,ti | 138232 | 10/02/2026 |
| #11 | 'health care':ab,ti | 612046 | 10/02/2026 |
| #10 | 'health polic*':ab,ti | 51836 | 10/02/2026 |
| #9 | 'health care policy'/exp | 248284 | 10/02/2026 |
| #8 | 'public poli*':ab,ti | 19278 | 10/02/2026 |
| #7 | 'public policy'/exp | 270944 | 10/02/2026 |
| #6 | #1 OR #2 OR #3 OR #4 OR #5 | 2246548 | 10/02/2026 |
| #5 | dermatosis:ab,ti | 14468 | 10/02/2026 |
| #4 | 'dermato* disease*':ab,ti | 5506 | 10/02/2026 |
| #3 | 'cutaneous disease*':ab,ti | 4512 | 10/02/2026 |
| #2 | 'skin disease*':ab,ti | 51361 | 10/02/2026 |
| #1 | 'skin disease'/exp | 2238859 | 10/02/2026 |

| **No.** | **Database:** | Embase | |
| --- | --- | --- | --- |
|  | **Search interface:** | Ovid | |
|  | **Search strategies** | **Records retrieved (n)** | **Search date** |
| #23 | #6 AND #20 AND #21 AND [embase]/lim AND [2015-2026]/py | 2004 | 10/02/2026 |
| #22 | #6 AND #20 AND #21 AND [embase]/lim | 2867 | 10/02/2026 |
| #21 | #7 OR #8 OR #9 OR #10 OR #11 OR #12 OR #13 OR #14 OR #15 OR #16 | 3767548 | 10/02/2026 |
| #20 | #17 OR #18 OR #19 | 410705 | 10/02/2026 |
| #19 | mexico:ab,ti OR belize:ab,ti OR honduras:ab,ti OR 'el salvador':ab,ti OR nicaragua:ab,ti OR 'costa rica':ab,ti OR Panama:ab,ti OR cuba:ab,ti OR haiti:ab,ti OR 'dominican republic':ab,ti OR colombia:ab,ti OR venezuela:ab,ti OR ecuador:ab,ti OR peru:ab,ti OR bolivia:ab,ti OR chile:ab,ti OR argentina:ab,ti OR uruguay:ab,ti OR 'paraguay':ab,ti OR Brazil:ab,ti OR 'puerto rico':ab,ti OR guyana:ab,ti OR suriname:ab,ti OR guatemala:ab,ti | 387154 | 10/02/2026 |
| #18 | 'latin america':ab,ti | 31171 | 10/02/2026 |
| #17 | south AND 'central america'/exp | 10633 | 10/02/2026 |
| #16 | 'guideline*':ab,ti | 964821 | 10/02/2026 |
| #15 | 'recommendation*':ab,ti | 600379 | 10/02/2026 |
| #14 | 'strategic planning'/exp | 7967 | 10/02/2026 |
| #13 | 'program*':ab,ti | 1751174 | 10/02/2026 |
| #12 | 'health system*':ab,ti | 138232 | 10/02/2026 |
| #11 | 'health care':ab,ti | 612046 | 10/02/2026 |
| #10 | 'health polic*':ab,ti | 51836 | 10/02/2026 |
| #9 | 'health care policy'/exp | 248284 | 10/02/2026 |
| #8 | 'public poli*':ab,ti | 19278 | 10/02/2026 |
| #7 | 'public policy'/exp | 270944 | 10/02/2026 |
| #6 | #1 OR #2 OR #3 OR #4 OR #5 | 2246548 | 10/02/2026 |
| #5 | dermatosis:ab,ti | 14468 | 10/02/2026 |
| #4 | 'dermato* disease*':ab,ti | 5506 | 10/02/2026 |
| #3 | 'cutaneous disease*':ab,ti | 4512 | 10/02/2026 |
| #2 | 'skin disease*':ab,ti | 51361 | 10/02/2026 |
| #1 | 'skin disease'/exp | 2238859 | 10/02/2026 |

| **No.** | **Database:** | Scopus | |
| --- | --- | --- | --- |
|  | **Search interface:** | Scopus | |
|  | **Search strategies** | **Records retrieved (n)** | **Search date** |
| #1 | ( ALL ( "skin disease*" ) OR ALL ( "cutan* disease*" ) OR ALL ( "dermato* disease*" ) OR ALL ( dermatosis ) ) | 369764 | 11/02/2026 |
| #2 | ( TITLE-ABS-KEY ( public policy ) OR TITLE-ABS-KEY ( health care policy ) OR TITLE-ABS-KEY ( public health ) OR TITLE-ABS-KEY ( program* ) OR TITLE-ABS-KEY ( strategic planning ) OR TITLE-ABS-KEY ( recommendation* ) OR TITLE-ABS-KEY ( guideline* ) OR TITLE-ABS-KEY ( health care ) OR TITLE-ABS-KEY ( health system ) ) | 10544762 | 11/02/2026 |
| #3 | ( TITLE-ABS-KEY ( latin america ) OR TITLE-ABS-KEY ( south america ) OR TITLE-ABS-KEY ( Central America ) OR TITLE-ABS-KEY ( mexico OR guatemala OR belize OR honduras OR "el salvador" OR nicaragua OR "costa rica" OR Panama OR cuba OR haiti OR "dominican republic" OR colombia OR venezuela OR ecuador OR peru OR bolivia OR chile OR argentina OR uruguay OR paraguay OR Brazil OR "puerto rico" OR guyana OR suriname ) ) | 1420631 | 11/02/2026 |
| #4 | ( ALL ( "skin disease*" ) OR ALL ( "cutan* disease*" ) OR ALL ( "dermato* disease" ) OR ALL ( dermatosis ) ) AND ( TITLE-ABS-KEY ( public policy ) OR TITLE-ABS-KEY ( health care policy ) OR TITLE-ABS-KEY ( public health ) OR TITLE-ABS-KEY ( program* ) OR TITLE-ABS-KEY ( strategic planning ) OR TITLE-ABS-KEY ( recommendation* ) OR TITLE-ABS-KEY ( guideline* ) OR TITLE-ABS-KEY ( health care ) OR TITLE-ABS-KEY ( health system ) ) AND ( TITLE-ABS-KEY ( latin america ) OR TITLE-ABS-KEY ( south america ) OR TITLE-ABS-KEY ( Central America ) OR TITLE-ABS-KEY ( mexico OR guatemala OR belize OR honduras OR "el salvador" OR nicaragua OR "costa rica" OR Panama OR cuba OR haiti OR "dominican republic" OR colombia OR venezuela OR ecuador OR peru OR bolivia OR chile OR argentina OR uruguay OR paraguay OR Brazil OR "puerto rico" OR guyana OR suriname ) ) | 1161 | 11/02/2026 |
| #5 | ( ALL ( "skin disease*" ) OR ALL ( "cutan* disease*" ) OR ALL ( "dermato* disease" ) OR ALL ( dermatosis ) ) AND ( TITLE-ABS-KEY ( public policy ) OR TITLE-ABS-KEY ( health care policy ) OR TITLE-ABS-KEY ( public health ) OR TITLE-ABS-KEY ( program* ) OR TITLE-ABS-KEY ( strategic planning ) OR TITLE-ABS-KEY ( recommendation* ) OR TITLE-ABS-KEY ( guideline* ) OR TITLE-ABS-KEY ( health care ) OR TITLE-ABS-KEY ( health system ) ) AND ( TITLE-ABS-KEY ( latin america ) OR TITLE-ABS-KEY ( south america ) OR TITLE-ABS-KEY ( Central America ) OR TITLE-ABS-KEY ( mexico OR guatemala OR belize OR honduras OR "el salvador" OR nicaragua OR "costa rica" OR Panama OR cuba OR haiti OR "dominican republic" OR colombia OR venezuela OR ecuador OR peru OR bolivia OR chile OR argentina OR uruguay OR paraguay OR Brazil OR "puerto rico" OR guyana OR suriname ) ) AND PUBYEAR > 2014 AND PUBYEAR < 2027 | 732 | 11/02/2026 |

| **No.** | **Database:** | EBSCO | |
| --- | --- | --- | --- |
|  | **Search interface:** | EBSCO | |
|  | **Search strategies** | **Records retrieved (n)** | **Search date** |
| #1 | skin disease* OR cutan* disease* OR dermato* disease* OR dermatosis | 193022 | 11/02/2026 |
| #2 | public policy OR health care policy OR public health OR program* OR strategic planning OR recommendation* OR guideline* OR health care OR health system | 20551253 | 11/02/2026 |
| #3 | latin america OR south america OR Central America OR (mexico OR guatemala OR belize OR honduras OR "el salvador" OR nicaragua OR "costa rica" OR Panama OR cuba OR haiti OR "dominican republic" OR colombia OR venezuela OR ecuador OR peru OR bolivia OR chile OR argentina OR uruguay OR paraguay OR Brazil OR "puerto rico" OR guyana OR suriname) | 4852648 | 11/02/2026 |
| #4 | ("latin america" OR "south america" OR "Central America" OR mexico OR guatemala OR belize OR honduras OR "el salvador" OR nicaragua OR "costa rica" OR Panama OR cuba OR haiti OR "dominican republic" OR colombia OR venezuela OR ecuador OR peru OR bolivia OR chile OR argentina OR uruguay OR paraguay OR Brazil OR "puerto rico" OR guyana OR suriname) AND (public policy OR health care policy OR public health OR program* OR strategic planning OR recommendation* OR guideline* OR health care OR health system) AND (skin disease* OR cutan* disease* OR dermato* disease* OR dermatosis) | 1019 | 11/02/2026 |
| #5 | ("latin america" OR "south america" OR "Central America" OR mexico OR guatemala OR belize OR honduras OR "el salvador" OR nicaragua OR "costa rica" OR Panama OR cuba OR haiti OR "dominican republic" OR colombia OR venezuela OR ecuador OR peru OR bolivia OR chile OR argentina OR uruguay OR paraguay OR Brazil OR "puerto rico" OR guyana OR suriname) AND (public policy OR health care policy OR public health OR program* OR strategic planning OR recommendation* OR guideline* OR health care OR health system) AND (skin disease* OR cutan* disease* OR dermato* disease* OR dermatosis) | 850  (Duplicates removed during export) | 11/02/2026 |

| **No.** | **Database:** | LILACS | |
| --- | --- | --- | --- |
|  | **Search interface:** | BVS | |
|  | **Search strategies** | **Records retrieved (n)** | **Search date** |
| #1 | ((skin disease*) OR (cutan* disease*) OR (dermato* disease*) OR (dermatosis)) AND ((public policy) OR (health care policy ) OR (public health ) OR (program* ) OR (strategic planning ) OR (recommendation*) OR (guideline* ) OR (health care) OR (health system)) AND ((latin america ) OR (south america ) OR (Central America) OR (mexico OR guatemala OR belize OR honduras OR (el salvador) OR nicaragua OR (costa rica) OR panama OR cuba OR haiti OR (dominican republic) OR colombia OR venezuela OR ecuador OR peru OR bolivia OR chile OR argentina OR uruguay OR paraguay OR Brazil OR (puerto rico) OR guyana OR suriname)) | 985 | 11/02/2026 |

| **No.** | **Database:** | Web of Science | |
| --- | --- | --- | --- |
|  | **Search interface:** | Web of Science | |
|  | **Search strategies** | **Records retrieved (n)** | **Search date** |
| #1 | (((TS=(skin disease*)) OR TS=(cutan* disease*)) OR TS=(dermato* disease* )) OR TS=(dermatosis) | 211708 | 11/02/2026 |
| #2 | (((((((((TS=(public policy)) OR TS=(health care policy)) OR TS=( public health)) OR TS=(program*)) OR TS=(strategic planning)) OR TS=(recommendation* )) OR TS=(guideline*)) OR TS=(health care)) OR TS=(health system)) | 4738797 | 11/02/2026 |
| #3 | ((((TS=(latin america)) OR TS=(south america)) OR TS=(Central America)) OR TS=(mexico OR guatemala OR belize OR honduras OR "el salvador" OR nicaragua OR "costa rica" OR Panama OR cuba OR haiti OR "dominican republic" OR colombia OR venezuela OR ecuador OR peru OR bolivia OR chile OR argentina OR uruguay OR paraguay OR Brazil OR "puerto rico" OR guyana OR suriname )) | 862301 | 11/02/2026 |
| #4 | ((((TS=(skin disease*)) OR TS=(cutan* disease*)) OR TS=(dermato* disease*)) OR TS=(dermatosis)) AND (((((((((TS=(public policy)) OR TS=(health care policy)) OR TS=( public health)) OR TS=(program*)) OR TS=(strategic planning)) OR TS=(recommendation* )) OR TS=(guideline*)) OR TS=(health care)) OR TS=(health system)) AND ((((TS=(latin america)) OR TS=(south america)) OR TS=(Central America)) OR TS=(mexico OR guatemala OR belize OR honduras OR "el salvador" OR nicaragua OR "costa rica" OR Panama OR cuba OR haiti OR "dominican republic" OR colombia OR venezuela OR ecuador OR peru OR bolivia OR chile OR argentina OR uruguay OR paraguay OR Brazil OR "puerto rico" OR guyana OR suriname )) | 1033 | 11/02/2026 |

**Grey literature search**

|  | **Search 1** |
| --- | --- |
| **Source** | Google scholar |
| **Search date** | 12/04/2026 |
| **Search limits** | 2015-2026 No citation searching |
| **Search strategy** | (dermatología OR "enfermedades dermatológicas" OR "enfermedades de la piel") AND ("política pública" OR programas OR estrategias) AND ("latinoamérica" OR méxico OR belice OR honduras OR "el salvador" OR nicaragua OR "costa rica" OR panamá OR cuba OR haití OR "república dominicana" OR colombia OR venezuela OR ecuador OR perú OR bolivia OR chile OR argentina OR uruguay OR paraguay OR brasil OR "puerto rico" OR guyana OR surinam OR guatemala) |
| **Records retrieved (n) (n)** | First 30 results; sorted by relevance |
|  |  |
|  | **Search 2** |
| **Source** | Google scholar |
| **Search date** | 12/04/2026 |
| **Search limits** | 2015-2026 No citation searching |
| **Search strategy** | (dermatology OR "skin diseases" OR "cutaneous diseases" OR "skin conditions") AND ("public policy" OR "health policy" OR programs OR strategies) AND ("latin america" OR mexico OR belize OR honduras OR "el salvador" OR nicaragua OR "costa rica" OR Panama OR cuba OR haiti OR "dominican republic" OR colombia OR venezuela OR ecuador OR peru OR bolivia OR chile OR argentina OR uruguay OR paraguay OR Brazil OR "puerto rico" OR guyana OR suriname OR guatemala) |
| **Records retrieved (n) (n)** | First 30 results; sorted by relevance |

**Website searches**

| **Country** | **Website consulted** | **Date** | **Search strategy/ observation** | **Results** |
| --- | --- | --- | --- | --- |
| Argentina | <https://www.argentina.gob.ar/salud> | 3/03/2026 | site:argentina.gob.ar/salud (política OR programa OR guía OR protocolo OR plan OR ruta OR estrategia OR prevención OR control OR vigilancia OR ley OR resolución) (teledermatología OR "Carcinoma basocelular" OR "Carcinoma escamocelular" OR Melanoma)  site:argentina.gob.ar/salud (política OR programa OR guía OR protocolo OR plan OR ruta OR estrategia OR prevención OR control OR vigilancia OR ley OR resolución) ("Pénfigo" OR "Dermatitis herpetiforme" OR Esclerodermia OR Lupus)  site:argentina.gob.ar/salud (política OR programa OR guía OR protocolo OR plan OR ruta OR estrategia OR prevención OR control OR vigilancia OR ley OR resolución) (Paniculitis OR "Eritema nodoso" OR "Paniculitis lúpica" OR Celulitis OR Lipoma OR Angiolipoma OR "Quiste epidermoide" OR "Absceso subcutáneo")  site:argentina.gob.ar/salud (política OR programa OR guía OR protocolo OR plan OR ruta OR estrategia OR prevención OR control OR vigilancia OR ley OR resolución) (Impétigo OR Erisipela OR Foliculitis OR Herpes OR Verrugas OR VPH OR "Molusco contagioso" OR Sarampión OR varicela OR viruela OR mpox)  site:argentina.gob.ar/salud (política OR programa OR guía OR protocolo OR plan OR ruta OR estrategia OR prevención OR control OR vigilancia OR ley OR resolución) ("Tiña" OR "Candidiasis cutánea" OR Onicomicosis OR Micetoma OR Esporotricosis OR Cromoblastomicosis)  site:argentina.gob.ar/salud (política OR programa OR guía OR protocolo OR plan OR ruta OR estrategia OR prevención OR control OR vigilancia OR ley OR resolución) (Escabiosis OR Pediculosis OR "Larva migrans cutánea" OR Tungiasis OR Miasis)  site:argentina.gob.ar/salud (política OR programa OR guía OR protocolo OR plan OR ruta OR estrategia OR prevención OR control OR vigilancia OR ley OR resolución) (Lepra OR Leishmaniasis OR rickettsia OR Oncocercosis OR "Úlcera de Buruli")  site:argentina.gob.ar/salud (política OR programa OR guía OR protocolo OR plan OR ruta OR estrategia OR prevención OR control OR vigilancia OR ley OR resolución) (Psoriasis OR Dermatitis OR "Liquen plano" OR Rosácea OR "Urticaria crónica" OR "Hidradenitis supurativa")  site:argentina.gob.ar/salud (política OR programa OR guía OR protocolo OR plan OR ruta OR estrategia OR prevención OR control OR vigilancia OR ley OR resolución) (Alopecia OR "Acné vulgar" OR Hiperhidrosis OR Onicocriptosis OR Vitiligo OR Melasma OR "Hiperpigmentación postinflamatoria") | 11 |
| Belize |  | 7/03/2026 | No government websites were identified. | 0 |
| Bolivia | [https://www.asuss.gob.bo/ https://www.minsalud.gob.bo/](https://www.asuss.gob.bo/) | 7/03/2026 | site:gob.bo (política OR programa OR guía OR protocolo OR plan OR ruta OR estrategia OR prevención OR control OR vigilancia OR ley OR resolución) (teledermatología OR "Carcinoma basocelular" OR "Carcinoma escamocelular" OR Melanoma) site:gob.bo (política OR programa OR guía OR protocolo OR plan OR ruta OR estrategia OR prevención OR control OR vigilancia OR ley OR resolución) ("Pénfigo" OR "Dermatitis herpetiforme" OR Esclerodermia OR Lupus) site:gob.bo (política OR programa OR guía OR protocolo OR plan OR ruta OR estrategia OR prevención OR control OR vigilancia OR ley OR resolución) (Paniculitis OR "Eritema nodoso" OR "Paniculitis lúpica" OR Celulitis OR Lipoma OR Angiolipoma OR "Quiste epidermoide" OR "Absceso subcutáneo") site:gob.bo (política OR programa OR guía OR protocolo OR plan OR ruta OR estrategia OR prevención OR control OR vigilancia OR ley OR resolución) (Impétigo OR Erisipela OR Foliculitis OR Herpes OR Verrugas OR VPH OR "Molusco contagioso" OR Sarampión OR varicela OR viruela OR mpox) site:gob.bo (política OR programa OR guía OR protocolo OR plan OR ruta OR estrategia OR prevención OR control OR vigilancia OR ley OR resolución) ("Tiña" OR "Candidiasis cutánea" OR Onicomicosis OR Micetoma OR Esporotricosis OR Cromoblastomicosis) site:gob.bo (política OR programa OR guía OR protocolo OR plan OR ruta OR estrategia OR prevención OR control OR vigilancia OR ley OR resolución) (Escabiosis OR Pediculosis OR "Larva migrans cutánea" OR Tungiasis OR Miasis) site:gob.bo (política OR programa OR guía OR protocolo OR plan OR ruta OR estrategia OR prevención OR control OR vigilancia OR ley OR resolución) (Lepra OR Leishmaniasis OR rickettsia OR Oncocercosis OR "Úlcera de Buruli" OR "Enfermedad de Lyme") site:gob.bo (política OR programa OR guía OR protocolo OR plan OR ruta OR estrategia OR prevención OR control OR vigilancia OR ley OR resolución) (Psoriasis OR Dermatitis OR "Liquen plano" OR Rosácea OR "Urticaria crónica" OR "Hidradenitis supurativa") site:gob.bo (política OR programa OR guía OR protocolo OR plan OR ruta OR estrategia OR prevención OR control OR vigilancia OR ley OR resolución) (Alopecia OR "Acné vulgar" OR Hiperhidrosis OR Onicocriptosis OR Vitiligo OR Melasma OR "Hiperpigmentación postinflamatoria") | 3 |
| Brazil | <https://www.gov.br/> | 7/03/2026 | site:gov.br/saude (política OR programa OR guia OR protocolo OR plano OR estratégia OR prevenção OR controle OR vigilância OR lei OR portaria) (teledermatologia OR "Carcinoma basocelular" OR "Carcinoma espinocelular" OR Melanoma) site:gov.br/saude (política OR programa OR guia OR protocolo OR plano OR estratégia OR prevenção OR controle OR vigilância OR lei OR portaria) ("Pênfigo" OR "Dermatite herpetiforme" OR Esclerodermia OR Lúpus) site:gov.br/saude (política OR programa OR guia OR protocolo OR plano OR estratégia OR prevenção OR controle OR vigilância OR lei OR portaria) (Paniculite OR "Eritema nodoso" OR "Paniculite lúpica" OR Celulite OR Lipoma OR Angiolipoma OR "Cisto epidermoide" OR "Abscesso subcutâneo") site:gov.br/saude (política OR programa OR guia OR protocolo OR plano OR estratégia OR prevenção OR controle OR vigilância OR lei OR portaria) (Impetigo OR Erisipela OR Foliculite OR Herpes OR Verrugas OR HPV OR "Molusco contagioso" OR Sarampo OR varicela OR variola OR mpox) site:gov.br/saude (política OR programa OR guia OR protocolo OR plano OR estratégia OR prevenção OR controle OR vigilância OR lei OR portaria) ("Tinea" OR "Candidíase cutânea" OR Onicomicose OR Micetoma OR Esporotricose OR Cromoblastomicose) site:gov.br/saude (política OR programa OR guia OR protocolo OR plano OR estratégia OR prevenção OR controle OR vigilância OR lei OR portaria) (Escabiose OR Pediculose OR "Larva migrans cutânea" OR Tungíase OR Miíase) site:gov.br/saude (política OR programa OR guia OR protocolo OR plano OR estratégia OR prevenção OR controle OR vigilância OR lei OR portaria) (Hanseníase OR Leishmaniose OR rickettsia OR Oncocercose OR "Úlcera de Buruli" OR "Doença de Lyme") site:gov.br/saude (política OR programa OR guia OR protocolo OR plano OR estratégia OR prevenção OR controle OR vigilância OR lei OR portaria) (Psoríase OR Dermatite OR "Líquen plano" OR Rosácea OR "Urticária crônica" OR "Hidradenite supurativa") site:gov.br/saude (política OR programa OR guia OR protocolo OR plano OR estratégia OR prevenção OR controle OR vigilância OR lei OR portaria) (Alopecia OR "Acre vulgar" OR Hiperidrose OR Onicocriptose OR Vitiligo OR Melasma OR "Hiperpigmentação pós-inflamatória") | 10 |
| Chile | <https://diprece.minsal.cl/> | 6/03/2026 10/03/2026 | Handsearching | 6 |
| Colombia | [https://www.minsalud.gov.co/ https://www.ins.gov.co](https://www.minsalud.gov.co/) | 7/03/2026 | site:gov.co (política OR programa OR guía OR protocolo OR plan OR ruta OR RIAS OR estrategia OR prevención OR control OR vigilancia OR ley OR resolución) (teledermatología OR "Carcinoma basocelular" OR "Carcinoma escamocelular" OR Melanoma) site:gov.co (política OR programa OR guía OR protocolo OR plan OR ruta OR RIAS OR estrategia OR prevención OR control OR vigilancia OR ley OR resolución) ("Pénfigo" OR "Dermatitis herpetiforme" OR Esclerodermia OR Lupus) site:gov.co (política OR programa OR guía OR protocolo OR plan OR ruta OR RIAS OR estrategia OR prevención OR control OR vigilancia OR ley OR resolución) (Paniculitis OR "Erythema nodoso" OR "Paniculitis lúpica" OR Celulitis OR Lipoma OR Angiolipoma OR "Quiste epidermoide" OR "Absceso subcutáneo") site:gov.co (política OR programa OR guía OR protocolo OR plan OR ruta OR RIAS OR estrategia OR prevención OR control OR vigilancia OR ley OR resolución) (Impétigo OR Erisipela OR Foliculitis OR Herpes OR Verrugas OR VPH OR "Molusco contagioso" OR Sarampión OR varicela OR viruela OR mpox)  site:gov.co (política OR programa OR guía OR protocolo OR plan OR ruta OR RIAS OR estrategia OR prevención OR control OR vigilancia OR ley OR resolución) ("Tiña" OR "Candidiasis cutánea" OR Onicomicosis OR Micetoma OR Esporotricosis OR Cromoblastomicosis)  site:gov.co (política OR programa OR guía OR protocolo OR plan OR ruta OR RIAS OR estrategia OR prevención OR control OR vigilancia OR ley OR resolución) (Escabiosis OR Pediculosis OR "Larva migrans cutánea" OR Tungiasis OR Miasis)  site:gov.co (política OR programa OR guía OR protocolo OR plan OR ruta OR RIAS OR estrategia OR prevención OR control OR vigilancia OR ley OR resolución) (Lepra OR Leishmaniasis OR rickettsia OR Oncocercosis OR "Úlcera de Buruli" OR "Enfermedad de Lyme")  site:gov.co (política OR programa OR guía OR protocolo OR plan OR ruta OR RIAS OR estrategia OR prevención OR control OR vigilancia OR ley OR resolución) (Psoriasis OR Dermatitis OR "Liquen plano" OR Rosácea OR "Urticaria crónica" OR "Hidradenitis supurativa")  site:gov.co (política OR programa OR guía OR protocolo OR plan OR ruta OR RIAS OR estrategia OR prevención OR control OR vigilancia OR ley OR resolución) (Alopecia OR "Acné vulgar" OR Hiperhidrosis OR Onicocriptosis OR Vitiligo OR Melasma OR "Hiperpigmentación postinflamatoria") | 11 |
| Costa Rica | [https://www.imprentanacional.go.cr/ https://www.ministeriodesalud.go.cr](https://www.imprentanacional.go.cr/) | 7/03/2026 | Handsearching | 8 |
| Cuba | <https://especialidades.sld.cu/> | 7/03/2026 | Handsearching | 1 |
| Ecuador | <https://www.salud.gob.ec/> | 7/03/2026 | Handsearching | 6 |
| El Salvador | <https://asp.salud.gob.sv/regulacion/default.asp> | 19/03/2026 | Handsearching | 19* |
| Guatemala | [https://osarguatemala.org/ley-de-vacunacion-decreto-6-2022/ https://www.igssgt.org/ https://epidemiologia.mspas.gob.gt/](https://osarguatemala.org/ley-de-vacunacion-decreto-6-2022/) | 10/03/2026 | Handsearching | 7 |
| Haití | Idioma francés |  | Handsearching | 0 |
| Honduras | <https://www.salud.gob.hn/sshome/> | 13/03/2026 14/03/2026 | Handsearching | 6 |
| Mexico | [www.imss.gob.mx https://www.gob.mx/](http://www.imss.gob.mx/) | 6/03/2026 08/03/2026 | site:gob.mx (política OR programa OR guía OR protocolo OR plan OR ruta OR estrategia OR prevención OR control OR vigilancia OR ley OR resolución) (teledermatología OR "Carcinoma basocelular" OR "Carcinoma escamocelular" OR Melanoma) site:gob.mx (política OR programa OR guía OR protocolo OR plan OR ruta OR estrategia OR prevención OR control OR vigilancia OR ley OR resolución) ("Pénfigo" OR "Dermatitis herpetiforme" OR Esclerodermia OR Lupus) site:gob.mx (política OR programa OR guía OR protocolo OR plan OR ruta OR estrategia OR prevención OR control OR vigilancia OR ley OR resolución) (Paniculitis OR "Eritema nodoso" OR "Paniculitis lúpica" OR Celulitis OR Lipoma OR Angiolipoma OR "Quiste epidermoide" OR "Absceso subcutáneo") site:gob.mx (política OR programa OR guía OR protocolo OR plan OR ruta OR estrategia OR prevención OR control OR vigilancia OR ley OR resolución) (Impétigo OR Erisipela OR Foliculitis OR Herpes OR Verrugas OR VPH OR "Molusco contagioso" OR Sarampión OR varicela OR viruela OR mpox) site:gob.mx (política OR programa OR guía OR protocolo OR plan OR ruta OR estrategia OR prevención OR control OR vigilancia OR ley OR resolución) ("Tiña" OR "Candidiasis cutánea" OR Onicomicosis OR Micetoma OR Esporotricosis OR Cromoblastomicosis) site:gob.mx (política OR programa OR guía OR protocolo OR plan OR ruta OR estrategia OR prevención OR control OR vigilancia OR ley OR resolución) (Escabiosis OR Pediculosis OR "Larva migrans cutánea" OR Tungiasis OR Miasis) site:gob.mx (política OR programa OR guía OR protocolo OR plan OR ruta OR estrategia OR prevención OR control OR vigilancia OR ley OR resolución) (Lepra OR Leishmaniasis OR rickettsia OR Oncocercosis OR "Úlcera de Buruli" OR "Enfermedad de Lyme") site:gob.mx (política OR programa OR guía OR protocolo OR plan OR ruta OR estrategia OR prevención OR control OR vigilancia OR ley OR resolución) (Psoriasis OR Dermatitis OR "Liquen plano" OR Rosácea OR "Urticaria crónica" OR "Hidradenitis supurativa") site:gob.mx (política OR programa OR guía OR protocolo OR plan OR ruta OR estrategia OR prevención OR control OR vigilancia OR ley OR resolución) (Alopecia OR "Acné vulgar" OR Hiperhidrosis OR Onicocriptosis OR Vitiligo OR Melasma OR "Hiperpigmentación postinflamatoria") | 11 |
| Nicaragua | <https://www.minsa.gob.ni/> | 13/03/2026 | Handsearching | 1 |
| Panama | [https://www.minsa.gob.pa](https://www.minsa.gob.pa/) | 13/03/2026 | Handsearching | 12 |
| Paraguay | [https://dgvs.mspbs.gov.py](https://dgvs.mspbs.gov.py/) | 12/03/2026 13/03/2026 | Handsearching | 9 |
| Peru | <https://cdn.www.gob.pe/> | 13/03/2026 14/03/2026 | Handsearching | 7 |
| Dominican Republic | <https://repositorio.msp.gob.do/> | 13/03/2026 | Handsearching | 2 |
| Uruguay | <https://www.gub.uy/> | 13/03/2026 | Handsearching | 3 |
| Venezuela | <https://mpps.gob.ve/> | 13/03/2026 | No documents were identified. Access to epidemiological protocols could not be obtained. One document was identified through the database search | 0* |
| PAHO |  | 3/03/2026 | Snowball searching | 4* |
| *One document identified through the database search was included with these documents for data extraction. | | | | |

Source: Authors

**TABLE S2** List of dermatological conditions

| Basal cell carcinoma | Onychomycosis |
| --- | --- |
| Squamous cell carcinoma | Scabies |
| Melanoma | Pediculosis |
| Pemphigus | Cutaneous larva migrans |
| Pemphigus vulgaris | Buruli ulcer |
| Bullous pemphigoid | Leprosy |
| Dermatitis herpetiformis | Cutaneous leishmaniasis |
| Scleroderma | Diffuse cutaneous leishmaniasis |
| Cutaneous lupus | Mucocutaneous leishmaniasis |
| Panniculitis | Rickettsial infections |
| Erythema nodosum | Onchocerciasis |
| Lupus panniculitis | Cutaneous myiasis |
| Cellulitis | Tungiasis |
| Lipoma | Psoriasis |
| Angiolipoma | Atopic dermatitis |
| Epidermoid cyst | Contact dermatitis |
| Subcutaneous abscess | Seborrheic dermatitis |
| Impetigo | Lichen planus |
| Erysipelas | Rosacea |
| Cellulite | Chronic urticaria |
| Folliculitis | Hidradenitis suppurativa |
| Herpes simplex | Mycetoma |
| Herpes zoster | Sporotrichosis |
| Warts (HPV) | Chromoblastomycosis |
| Molluscum contagiosum | Alopecia areata |
| Measles | Androgenetic alopecia |
| Chickenpox | Acne vulgaris |
| Smallpox | Hyperhidrosis |
| Mpox | Onychocryptosis |
| Tinea corporis | Vitiligo |
| Tinea pedis | Melasma |
| Tinea capitis | Post-inflammatory hyperpigmentation |
| Cutaneous candidiasis |  |

**TABLE S3** List of included and excluded studies

**List of included studies - electronic databases**

| **No.** | **Author, year** | **Title** |
| --- | --- | --- |
| 1 | WHO, 2017 | Progress towards eliminating onchocerciasis in the WHO Region of the Americas: elimination of transmission in the north-east focus of the Bolivarian Republic of Venezuela |
| 2 | Abi-Saab, 2021 | Economic assessment of teledermatology in the state of Santa Catarina - Brazil |
| 3 | Ahmed, 2022 | The effect of conditional cash transfers on the control of neglected tropical disease: a systematic review |
| 4 | Falleiros-Arlant, 2019 | Burden of varicella in Latin America and the Caribbean: findings from a systematic literature review |
| 5 | Rodrigues-Barbieri, 2016 | Impact of a Reference Center on Leprosy Control under a Decentralized Public Health Care Policy in Brazil |
| 6 | Barrenechea, 2025 | Impact of single-dose varicella vaccination in Argentina, by province: a time series study |
| 7 | Beltrán, 2016 | Leprosy Drug Resistance Surveillance in Colombia: The Experience of a Sentinel Country |
| 8 | Bierrenbach, 2025 | Impact of HPV vaccination on the hospitalizations for anogenital warts and high-grade cervical intraepithelial neoplasia in Brazil: A national analysis |
| 9 | Botto, 2016 | Evidence of suppression of onchocerciasis transmission in the Venezuelan Amazonian focus |
| 10 | Buzza, 2019 | Overall Results for a National Program of Photodynamic Therapy for Basal Cell Carcinoma: A Multicenter Clinical Study to Bring New Techniques to Social Health Care |
| 11 | Buzza, 2016 | Photodynamic therapy: Progress toward a scientific and clinical network in Latin America |
| 12 | Casas, 2021 | Community Dermatology in Argentina |
| 13 | Criado, 2023 | Skin cancer prevention campaign in childhood: survey based on 3676 children in Brazil |
| 14 | da Costa, 2024 | A cost-effectiveness analysis of a novel algorithm to sequentially diagnose leprosy based on manufactured tests under the SUS perspective |
| 15 | Mazzuoccolo, 2020 | High impact of a national psoriasis telementoring clinic on medical practices for patients in underserved areas |
| 16 | de Abreu - Pinto, 2020 | Number of cases of varicella and hospitalization in a pediatric reference hospital in brazil after introducing the vaccine |
| 17 | de Andrade, 2018 | Effect of Brazil's Conditional Cash Transfer Programme on the new case detection rate of leprosy in children under 15 years old |
| 18 | de Araujo, 2024 | Progress Toward Onchocerciasis Elimination in Brazil |
| 19 | de Melo, 2024 | Evaluation of the public policy impacts on Monkeypox in Brazil |
| 20 | de Oliveira, 2021 | Socioeconomic impact of high-cost drugs in Brazilian dermatology. Legal and financial aspects, and impact on clinical practice |
| 21 | do Nascimento, 2024 | Creation and development of support groups for leprosy self-care in a state in the northeast of Brazil |
| 22 | Drenkard, 2022 | Let's Talk about Lupus. Overview of an Innovative, High-Reach, Online Program to Fill the Education Gaps of Latin Americans Living with Lupus |
| 23 | MINSAL, El Salvador, 2025 | Procedimiento para la vigilancia y control de la leishmaniasis |
| 24 | Falleiros-Arlant, 2017 | Varicella-related hospitalizations: Brazilian retrospective analysis after the inclusion of a vaccine in the national immunization program |
| 25 | Fernandez-Santos, 2020 | Post-elimination surveillance in formerly onchocerciasis endemic focus in southern mexico |
| 26 | Pujadas-Ferrer, 2023 | Varicella in Uruguay. Epidemiological situation and prevention strategies. Period 2008 to 2021 |
| 27 | Franca, 2023 | Spatial analysis reveals failures in leprosy control activities in a hyperendemic city in Brazil |
| 28 | Fuenzalida, 2025 | Pediatric Teledermatology in the General Teledermatology Practice: Population Variances in Contrasting Health Care Scenarios |
| 29 | Gatica, 2023 | Teledermatology Evaluation and Feedback Systems: A Tool for Improving Care |
| 30 | Gatica, 2015 | Store-and-forward teledermatogy in Chile: A contribution to primary health care |
| 31 | Gentile, 2022 | Impact of varicella vaccination in Argentina: Seroprevalence in children and adults in a pediatric hospital |
| 32 | Gentile, 2021 | Impact of varicella vaccine on nosocomial outbreaks and management of post exposure prophylaxis following in a paediatric hospital |
| 33 | Giglio, 2022 | Trends in Varicella Burden of Disease Following Introduction of Routine Childhood Varicella Vaccination in Argentina: A 12-Year Time Series Analysis |
| 34 | Giglio, 2023 | Estimating the health economic impact of the universal varicella vaccination in Argentina from 2015 to 2019 |
| 35 | Gonzalez-Coloma, 2019 | Teledermatology in Remote Parts of Chile: Experience in 4 Isolated Rural Areas |
| 36 | Grana, 2025 | Unveiling hidden leprosy in underserved populations of the Amazonas state through active case-finding- the Amazon Skin Health Program (2023) |
| 37 | Lages, 2025 | Correlation and Trends in Primary Health Care and Family Health Strategy Coverage of Leprosy Detection in Minas Gerais |
| 38 | Lipof, 2017 | Establishment of a dermatology global health outreach and residency partnership program in Guatemala |
| 39 | Luna, 2022 | ECHO project in atopic dermatitis in Argentina: An innovative strategy to reach underserved areas with up to date knowledge, first year of experience |
| 40 | Machado, 2022 | Spatio-temporal analysis of leprosy risks in a municipality in the state of Mato Grosso-Brazilian Amazon: results from the leprosy post-exposure prophylaxis program in Brazil |
| 41 | Maciel, 2015 | The Brazilian states' capacity in eliminating the hansen endemic |
| 42 | Maia-Elkhoury, 2017 | SisLeish: A multi-country standardized information system to monitor the status of Leishmaniasis in the Americas |
| 43 | Manubens, 2023 | Teledermatologí­a sincrónica en tiempos de Covid-19. Percepción del dermatólogo |
| 44 | Martos, 2022 | Community intervention programmes with people affected by leprosy: Listening to the voice of professionals |
| 45 | Mauro, 2019 | Adverse events following Quadrivalent HPV vaccination reported in Sao Paulo State, Brazil, in the first three years after introducing the vaccine for routine immunization (March 2014 to December 2016) |
| 46 | Mazzuoccolo, 2019 | WhatsApp: A Real-Time Tool to Reduce the Knowledge Gap and Share the Best Clinical Practices in Psoriasis |
| 47 | Medeiros, 2024 | Evaluation of the public health laboratory network for tegumentary leishmaniasis in an endemic area of Brazil |
| 48 | Mendizabal, 2021 | Cutaneous leishmaniasis control in Alta Verapaz (northern Guatemala): evaluating current efforts through stakeholders’ experiences |
| 49 | Messagier, 2019 | Teledermatology Use in Remote Areas of French Guiana: Experience From a Long-Running System |
| 50 | Miranda, 2023 | The impact of varicella vaccination: A 2005-2019 interrupted time series analysis |
| 51 | Moreno, 2016 | Alternative approaches for cutaneous leishmaniasis surveillance in indigenous areas - case study among the Amapa Wajapi people |
| 52 | Morice, 2023 | Health workers vaccination: Experience and lessons learned from Costa Rica |
| 53 | Nunes, 2025 | Sistema de vigilância da leishmaniose tegumentar no Ceará, 2012-2022 |
| 54 | OPS, 2019 | Programa de Control de Leishmaniasis. Normas, pautas y procedimientos para el diagnóstico y control |
| 55 | Pawaskar, 2022 | The impact of universal varicella vaccination on the clinical burden of varicella in Colombia: A National database Analysis, 2008-2019 |
| 56 | Pescarini, 2020 | Effect of a conditional cash transfer programme on leprosy treatment adherence and cure in patients from the nationwide 100 Million Brazilian Cohort: a quasi-experimental study |
| 57 | Poester, 2022 | Sporotrichosis hyperendemic in Southern Brazil: Twelve years of challenges |
| 58 | Ribeiro, 2020 | Impact of the tetra viral vaccine introduction on varicella morbidity and mortality in the Brazilian macro regions |
| 59 | Richards, 2015 | One Hundred Years After Its Discovery in Guatemala by Rodolfo Robles, Onchocerca volvulus Transmission Has Been Eliminated from the Central Endemic Zone |
| 60 | Richards, 2016 | A Knowledge, Attitudes and Practices Survey Conducted Three Years after Halting Ivermectin Mass Treatment for Onchocerciasis in Guatemala |
| 61 | Richardus, 2021 | Leprosy post-exposure prophylaxis with single-dose rifampicin (LPEP): an international feasibility programme |
| 62 | Estrada-Roberto, 2021 | Mycetoma and the Community Dermatology Program, Mexico |
| 63 | Romanholo, 2018 | Surveillance of intradomiciliary contacts of leprosy cases: perspective of the client in a hyperendemic municipality |
| 64 | Saboya, 2023 | Lessons learned from the implementation of integrated serosurveillance of communicable diseases in the Americas |
| 65 | Sanchez, 2017 | Clinical impact in the real life of guidelines recommendations for atopic dermatitis in a tropical population (TECCEMA cohort) |
| 66 | Scotta, 2018 | Early impact of universal varicella vaccination on childhood varicella and herpes zoster hospitalizations in Brazil |
| 67 | Silva, 2022 | Impact of six years of routine varicella vaccination on the disease-related hospitalizations at Minas Gerais, Brazil |
| 68 | Soares, 2023 | Integrated control of neglected tropical diseases in Brazil: document review of a national campaign in light of WHO recommendations |
| 69 | de Souza, 2017 | Assessment of the implementation of the Leprosy Control Program in Camaragibe, Pernambuco State, Brazil |
| 70 | Teixeira, 2025 | The Minha Casa Minha Vida social housing programme and leprosy in Brazil: an analysis of the 100 Million Brazilian Cohort (2010-2015) |
| 71 | Vitiritti, 2024 | Hidden leprosy in a low-endemic area in southern Brazil: changes in endemicity following an active search |
| 72 | von Wangenheim, 2019 | Creating a Web Infrastructure for the Support of Clinical Protocols and Clinical Management: An Example in Teledermatology |
| 73 | Wakiyama, 2017 | Initial basal cell carcinomas diagnosed in the National Campaign for Skin Cancer Prevention are smaller than those identified by the conventional medical referral system |
| 74 | Zonta, 2024 | Supporting a rapid primary care response to emergent communicable disease threats with PACK (Practical Approach to Care Kit) in Florianópolis, Brazil |

**List of included documents - web pages**

| **No.** | **Author, year** | **Document Title** | **Country/Region** |
| --- | --- | --- | --- |
| 1 | Ministerio de Salud de la Nación Argentina, 2023 | Notificación de Enfermedad Febril Exantemática -EFE- (Sarampión/ Rubéola) al Sistema Nacional de Vigilancia de la Salud (SNVS2.0) | Argentina |
| 2 | Ministerio de Salud de la Nación Argentina, 2024 | Lineamientos Técnicos y Manual de Vacunación. Vacuna contra el Virus del Papiloma Humano (VPH) | Argentina |
| 3 | Ministerio de Salud de la Nación Argentina, 2025 | Lineamientos de campaña focalizada contra sarampión en contexto de brote 2025 – AMBA | Argentina |
| 4 | Ministerio de Salud de la Nación Argentina, 2021 | Lineamientos técnicos Introducción de la segunda dosis de vacuna contra varicela al Calendario Nacional de Inmunizaciones | Argentina |
| 5 | Congreso de la Nación Argentina, 2018 | Ley 27491 Control de enfermedades prevenibles por vacunación | Argentina |
| 6 | Ministerio de Salud de la Nación Argentina, 2016 | Resolución 1977/2016 Incorporación vacuna VPH en varones y esquema dos dosis | Argentina |
| 7 | Superintendencia de Riesgos del Trabajo (SRT), 2018 | Guía de actuación y diagnóstico de enfermedades profesionales: Dermatitis por contacto | Argentina |
| 8 | Superintendencia de Riesgos del Trabajo (SRT), 2025 | Leishmaniasis | Argentina |
| 9 | Administración Nacional de Laboratorios e Institutos de Salud "Dr. Carlos G. Malbrán" (ANLIS-Malbrán), Ministerio de Salud de la Nación Argentina, 2016 | Guía de diagnóstico y tratamiento de la Fiebre Manchada por Rickettsia parkeri | Argentina |
| 10 | Ministerio de Salud de la Nación Argentina, 2018 | Consenso sobre prevención, diagnóstico y tratamiento de la lepra | Argentina |
| 11 | Pan American Health Organization (PAHO), 2023 | Manual de procedimientos para la vigilancia y el control de las leishmaniasis en la Región de las Américas | Region of the Americas |
| 12 | Pan American Health Organization (PAHO), 2024 | Plan de acción para fortalecer la vigilancia y el control de las leishmaniasis en las Américas 2023-2030 | Region of the Americas |
| 13 | Ministerio de Salud de la Nación Argentina, Administración Nacional de Laboratorios e Institutos de Salud "Dr. Carlos G. Malbrán" (ANLIS-Malbrán), 2022 | Red Nacional de Diagnóstico de Leishmaniasis | Argentina |
| 14 | Pan American Health Organization (PAHO), 2025 | Iniciativa para la Eliminación de Enfermedades 30+. Acelerar la eliminación de enfermedades transmisibles y problemas relacionados en la Región de las Américas: Marco de seguimiento y acción | Region of the Americas |
| 15 | Autoridad de Supervisión de la Seguridad Social de Corto Plazo (ASUSS), 2019 | Normas de Diagnóstico y Tratamiento Dermatología | Bolivia |
| 16 | Ministerio de Salud y Deportes – Dirección General de Epidemiología, 2022 | Guía para la vigilancia epidemiológica de la Viruela del Mono | Bolivia |
| 17 | Programa Nacional de Vigilancia y Control de la Leishmaniasis, Ministerio de Salud y Deportes, 2015 | Norma Nacional y Manual de Procedimientos Técnicos de Leishmaniasis | Bolivia |
| 18 | Ministerio de Salud, 2018 | Norma Técnica de Salud que establece el Esquema Nacional de Vacunación | Peru |
| 19 | Ministerio de Educación, 2025 | Protocolo de Bioseguridad para la prevención del Sarampión en instituciones educativas del Sistema Educativo Plurinacional | Bolivia |
| 20 | Ministerio da Saúde / CONITEC, 2022 | Diretrizes Diagnósticas e Terapêuticas – Melanoma Cutâneo | Brazil |
| 21 | Ministerio da Saúde, 2022 | Protocolo Clínico e Diretrizes Terapêuticas Lúpus Eritematoso Sistêmico | Brazil |
| 22 | Ministerio da Saúde, 2022 | Protocolo Clínico e Diretrizes Terapêuticas da Esclerose Sistêmica | Brazil |
| 23 | Ministerio da Saúde, 2024 | Atenção Primária Prisional – Guia Prático Dermatologia | Brazil |
| 24 | Ministerio da Saúde, 2024 | Guia de Vigilância em Saúde - 6a edição revisada | Brazil |
| 25 | Ministerio da Saúde, 2022 | Monkeypox: Orientações técnicas para a assistência à saúde | Brazil |
| 26 | Ministerio da Saúde, 2017 | Manual de Vigilância da Leishmaniose Tegumentar | Brazil |
| 27 | Ministerio da Saúde, 2017 | Guia prático sobre a hanseníase | Brazil |
| 28 | Ministerio da Saúde, 2022 | Protocolo Clínico e Diretrizes Terapêuticas da Hanseníase | Brazil |
| 29 | Ministerio da Saúde, 2024 | Estratégia Nacional para Enfrentamento à Hanseníase 2024-2030 | Brazil |
| 30 | Ministerio da Saúde, 2025 | Protocolo Clínico e Diretrizes Terapêuticas de Dermatite Atópica | Brazil |
| 31 | Ministerio da Saúde, 2019 | Protocolo Clínico e Diretrizes Terapêuticas da Hidradenite Supurativa. | Brazil |
| 32 | Ministerio da Saúde, 2021 | Protocolo Clínico e Diretrizes Terapêuticas da Psoríase | Brazil |
| 33 | Ministerio de Salud, 2020 | Decreto 55 Exento: Dispone vacunación obligatoria contra sarampión, rubéola y parotiditis a población que indica | Chile |
| 34 | Servicio de Salud Metropolitano Sur Oriente (SSMSO), Ministerio de Salud, 2022 | Protocolo de referencia y contrarreferencia de dermatología | Chile |
| 35 | Ministerio de Salud, 2013 (Update of the 1st edition of 2011). | Guía Clínica AUGE Lupus Eritematoso Sistémico | Chile |
| 36 | Servicio de Salud Metropolitano Sur Oriente (SSMSO), Ministerio de Salud, 2022 | Protocolo Resolución en Red General de Dermatología | Chile |
| 37 | Superintendencia de Seguridad Social (SUSESO), 2018 (current versión 2024–2025) | Compendio de Normas del Seguro Social de Accidentes del Trabajo y Enfermedades Profesionales - Protocolo de patologías dermatológicas | Chile |
| 38 | Ministerio de Salud, 2021 | Decreto 68 Exento: Modifica decreto que dispone vacunación obligatoria contra enfermedades inmunoprevenibles | Chile |
| 39 | Ministerio de Salud y Protección Social, 2014 | Guía de Práctica Clínica con evaluación económica para la prevención, diagnóstico, tratamiento y seguimiento del cáncer de piel no melanoma: carcinoma basocelular | Colombia |
| 40 | Ministerio de Salud y Protección Social, 2014 | Guía de práctica clínica con evaluación económica para la prevención, diagnóstico, tratamiento y seguimiento del cáncer de piel no melanoma: carcinoma escamocelular de piel | Colombia |
| 41 | Ministerio de Salud y Protección Social, 2014 | Guía de Práctica Clínica con evaluación económica para la prevención, diagnóstico, tratamiento y seguimiento del cáncer de piel no melanoma: queratosis actínica. Para uso de profesionales de salud. | Colombia |
| 42 | Ministerio de Salud y Protección Social, 2022 | Control de la escabiosis, en el marco del programa nacional de enfermedades infecciosas desatendidas. | Colombia |
| 43 | Instituto Nacional de Salud, 2024 | Protocolo de vigilancia en salud pública Mpox | Colombia |
| 44 | Instituto Nacional de Salud, 2024 | Protocolo de vigilancia en salud pública Sarampión y Rubéola | Colombia |
| 45 | Instituto Nacional de Salud, 2017 | Guía para la vigilancia por laboratorio de Mycobacterium leprae | Colombia |
| 46 | Ministerio de Salud y Protección Social, 2018 | Lineamientos para la atención clínica integral de leishmaniasis en Colombia | Colombia |
| 47 | Instituto Nacional de Salud, 2024 | Protocolo de vigilancia de Leishmaniasis | Colombia |
| 48 | Instituto Nacional de Salud, 2024 | Protocolo de vigilancia en salud pública Lepra o enfermedad de Hansen | Colombia |
| 49 | Ministerio de Salud y Protección Social, 2023 | Plan Nacional Prevención y control de la tungiasis | Colombia |
| 50 | Ministerio de Salud Pública, 2014 | Manual de normas para el tratamiento de cáncer en Costa Rica | Costa Rica |
| 51 | Comité Central de Farmacoterapia, Gerencia Médica, Caja Costarricense de Seguro Social 2024 | Lineamiento y formulario de verificación para el tratamiento farmacológico en pacientes con melanoma maligno en contexto adyuvante o metastásico. | Costa Rica |
| 52 | Dirección de Vigilancia de la Salud - Ministerio de Salud Pública, 2022 | Lineamiento General para la Vigilancia de la Viruela Símica | Costa Rica |
| 53 | Ministerio de Salud Pública, 2013 | Norma Nacional de Vacunación de Costa Rica | Costa Rica |
| 54 | Ministerio de Salud Pública, 2013 | Norma Nacional de Vacunación Costa Rica | Costa Rica |
| 55 | Ministerio de Salud Pública; Caja Costarricense del Seguro Social (CCSS); Centro Nacional de Referencia Virológica (INCIENSA), 2025 | Protocolo para la Vigilancia Epidemiológica del Sarampión-Rubéola | Costa Rica |
| 56 | Ministerio de Salud Pública; Caja Costarricense del Seguro Social (CCSS); INCIENSA; Pan American Health Organization (PAHO), 2022 | Protocolo para la vigilancia de la Leishmaniasis en Costa Rica | Costa Rica |
| 57 | Ministerio de Salud Pública,  INCIENSA; Caja Costarricense de Seguro Social; Servicio Nacional de Salud Animal, 2024 | Lineamiento Nacional para la Vigilancia de Miasis por Gusano Barrenador en Humanos | Costa Rica |
| 58 | Ministerio de Salud Pública, 2023 | Prevención, diagnóstico y tratamiento del cáncer de piel | Cuba |
| 59 | Ministerio de Salud Pública, 2016 | Guía de Práctica Clínica Diagnóstico y tratamiento del acné | Ecuador |
| 60 | Ministerio de Salud Pública, 2013 | Lupus Eritematoso Sistémico: Guía de Práctica Clínica | Ecuador |
| 61 | Ministerio de Salud Pública, 2022 | Lineamientos operativos para la viruela del mono o viruela símica | Ecuador |
| 62 | Ministerio de Salud Pública, 2021 | Lineamientos de la campaña de vacunación y recuperación del esquema regular | Ecuador |
| 63 | Ministerio de Salud Pública, 2025 | Protocolo de manejo clínico de la enfermedad de Hansen (lepra) | Ecuador |
| 64 | Ministerio de Salud Pública, 2025 | Estrategia Nacional de Interrupción de la Transmisión y Eliminación de la Enfermedad de Hansen (lepra) 2025-2030. | Ecuador |
| 65 | Instituto Salvadoreño Del Seguro Social, 2019 | Norma para la atención del cáncer en el ISSS | El Salvador |
| 66 | Ministerio De Salud, 2019 | Lineamientos técnicos para el rociado en el control de enfermedades de Chagas, leishmaniasis y malaria | El Salvador |
| 67 | Ministerio De Salud, 2019 | Lineamientos para la inclusión de la vacuna contra el virus del papiloma humano (VPH) al esquema nacional de vacunación. | El Salvador |
| 68 | Ministerio De Salud, 2022 | Lineamientos técnicos para la detección y control de casos de viruela símica | El Salvador |
| 69 | Ministerio De Salud, 2022 | Lineamientos técnicos para la vacunación contra la viruela símica | El Salvador |
| 70 | Ministerio De Salud, 2022 | Lineamientos técnicos para la prevención y control de la lepra | El Salvador |
| 71 | Ministerio De Salud, 2024 | Lineamientos técnicos para el control de las infecciones de transmisión sexual y hepatitis virales | El Salvador |
| 72 | Ministerio De Salud, 2024 | Lineamientos técnicos para la atención de las poblaciones clave en las clínicas de vigilancia centinela de las infecciones de transmisión sexual | El Salvador |
| 73 | Ministerio De Salud, 2024 | Lineamientos técnicos para la prevención y control de las enfermedades inmunoprevenibles | El Salvador |
| 74 | Ministerio De Salud, 2024 | Lineamientos técnicos para la prevención, vigilancia y control de enfermedades transmitidas por vectores y zoonosis | El Salvador |
| 75 | Ministerio De Salud, 2024 | Lineamientos técnicos del Sistema Nacional de Vigilancia Epidemiológica en El Salvador VIGEPES | El Salvador |
| 76 | Ministerio De Salud, 2025 | Lineamientos técnicos para la atención integral de la miasis causada por gusano barrenador en humanos | El Salvador |
| 77 | Ministerio De Salud, 2025 | Lineamientos técnicos para vacunación segura | El Salvador |
| 78 | Ministerio De Salud, 2025 | Lineamientos técnicos para la prevención y control del sarampión, rubéola, síndrome de rubéola congénita y parotiditis | El Salvador |
| 79 | Ministerio De Salud, 2023 | Norma técnica para la prevención y control de enfermedades inmunoprevenibles | El Salvador |
| 80 | Ministerio De Salud, 2014 | Acuerdo N ° 294.- Equipo de Gestión de Vigilancia Centinela Integrado de Enfermedades Inmunoprevenibles | El Salvador |
| 81 | Ministerio De Salud, 2014 | Plan nacional para la prevención, control y eliminación de las enfermedades infecciosas desatendidas | El Salvador |
| 82 | Ministerio De Salud, 2017 | Plan nacional de respuesta rápida ante casos de sarampión y rubéola | El Salvador |
| 83 | Ministerio De Salud, 2024 | Plan nacional para la prevención, el control y la eliminación de las enfermedades tropicales priorizadas en El Salvador. 2024-2026 | El Salvador |
| 84 | Congreso de la República de Guatemala - 2022 | Decreto Número 6-2022: Ley de Vacunación | Guatemala |
| 85 | Ministerio de Salud Pública y Asistencia Social (MSPAS) 2016 | Guía de prevención y atención Integral de cáncer | Guatemala |
| 86 | Instituto Guatemalteco de Seguridad Social (IGSS), 2018 edition (final revision: November 2019) | Guía de Práctica Clínica Basada en la Evidencia (GPC-BE) No. 113 “Lupus eritematoso sistémico” | Guatemala |
| 87 | Instituto Guatemalteco de Seguridad Social (IGSS), 2026 edition (revised and approved 2025) | Guía de Práctica Clínica Basada en la Evidencia (GPC-BE) No. 196 Enfermedades inmunoprevenibles: Sarampión y Rubeola | Guatemala |
| 88 | Ministerio de Salud Pública y Asistencia Social (MSPAS), Centro Nacional de Epidemiología, Guatemala, 2007 | Protocolos de Vigilancia Epidemiológica de Enfermedades Inmunoprevenibles | Guatemala |
| 89 | Ministerio de Salud Pública y Asistencia Social (MSPAS), Departamento de Epidemiología, Guatemala, 2018 | Protocolos de Vigilancia Epidemiológica de Enfermedades Vectoriales de Origen Parasitario; para este registro, corresponde a la sección 4. Leishmaniasis | Guatemala |
| 90 | Ministerio de Salud Pública y Asistencia Social (MSPAS), 2018 | Vigilancia Epidemiológica del Cáncer | Guatemala |
| 91 | Secretaría de Salud (SESAL), Programa Ampliado de Inmunizaciones  2023 | Manual para la Vigilancia Epidemiológica de las Enfermedades Prevenibles por Vacunación en Honduras | Honduras |
| 92 | Secretaría de Salud (SESAL), Programa Ampliado de Inmunizaciones (PAI), 2013 | Capítulo XIV. Vigilancia Epidemiológica del documento Normas y Procedimientos del Programa Ampliado de Inmunizaciones | Honduras |
| 93 | Secretaría de Salud (SESAL), 2024 | Lineamientos para la vigilancia epidemiológica, manejo clínico y prevención de miasis por gusano barrenador en humanos | Honduras |
| 94 | Secretaría de Salud (SESAL), 2022 | Lineamientos para el Abordaje Clínico y Vigilancia Epidemiológica de Viruela Símica Honduras | Honduras |
| 95 | Secretaría de Salud (SESAL),  2025 | Lineamiento para el manejo de casos sospechosos y confirmados de Sarampión en el Primer Nivel de Atención | Honduras |
| 96 | Secretaría de Salud (SESAL) - PAI, 2022 | Lineamientos técnicos y operativos de introducción de la Vacuna contra la Viruela Símica, Honduras | Honduras |
| 97 | Instituto Mexicano del Seguro Social (IMSS), 2011 | Diagnóstico y tratamiento de la queratosis Actínica | Mexico |
| 98 | Instituto Mexicano del Seguro Social (IMSS), 2012 | Diagnóstico y tratamiento de Escabiosis | Mexico |
| 99 | Instituto Mexicano del Seguro Social (IMSS), 2013 | Abordaje Terapéutico dermatológico ambulatorio del pénfigo vulgar en adultos | Mexico |
| 100 | Instituto Mexicano del Seguro Social (IMSS), 2013 | Tratamiento farmacológico para pacientes adultos con psoriasis en placas | Mexico |
| 101 | Instituto Mexicano del Seguro Social (IMSS), 2016 | Prevención primaria y detección oportuna del melanoma cutáneo en población general en el primer nivel de atención médica | Mexico |
| 102 | Instituto Mexicano del Seguro Social (IMSS), 2019 | Diagnóstico y tratamiento del carcinoma basocelular | Mexico |
| 103 | Secretaría de Salud de México / Centro Nacional para la Salud de la Infancia y la Adolescencia (CeNSIA). 2021 | Manual de Vacunación | Mexico |
| 104 | Secretaría de Salud de México (Dirección General de Epidemiología / InDRE), 2025 | Lineamientos para la vigilancia por laboratorio de mpox, Versión 2.0. | Mexico |
| 105 | Secretaría de Salud de México / InDRE.  2018 | Lineamientos para la Vigilancia por Laboratorio de la Enfermedades Febriles Exantemáticas. | Mexico |
| 106 | Secretaría de Salud de México, Subsecretaría de Políticas de Salud y Bienestar Poblacional, Dirección General de Epidemiología (DGE) - Instituto de Diagnóstico y Referencia Epidemiológicos, 2025 | Lineamientos para la vigilancia por laboratorio de la leishmaniasis | Mexico |
| 107 | Secretaría de Salud de México - Dirección general de epidemiología, 2019 | Manual de Procedimientos Estandarizados para la Vigilancia Epidemiológica de las Micobacteriosis | Mexico |
| 108 | Ministerio de Salud (MINSA), 2024 | Norma para el Manejo Integrado de Vectores | Nicaragua |
| 109 | Ministerio de Salud (MINSA), Caja de Seguro Social (CSS), Pan American Health Organization/World Health Organization (PAHO/WHO), and International Atomic Energy Agency (IAEA), 2019 | Plan Estratégico Nacional para la Prevención y Control del Cáncer 2019-2029 | Panama |
| 110 | Ministerio de Salud (MINSA), Caja de Seguro Social (CSS), Instituto Conmemorativo Gorgas de Estudios de la Salud (ICGES), Pan American Health Organization/World Health Organization (PAHO/WHO), 2019 | Guía para el abordaje integral de los pacientes con lepra | Panama |
| 111 | Ministerio de Salud (MINSA) (Dirección General de Salud Pública), 2022, version 4 | Guía Nacional para la Vigilancia, Prevención, Control y Atención de la Viruela Símica | Panama |
| 112 | Ministerio de Salud (MINSA), 2018 | Guía Nacional de Epidemiología | Panama |
| 113 | Ministerio de Salud (MINSA), Caja de seguro social, Dirección general de salud, Departamento de epidemiología, Programa ampliado de inmunizaciones, 2012 | Manual de normas y procedimientos del programa ampliado de inmunizaciones | Panama |
| 114 | Ministerio de Salud (MINSA), Dirección General de Salud Pública, 2022 | Plan Nacional para la Vigilancia, Prevención, Control y Atención de la Viruela Símica | Panama |
| 115 | Ministerio de Salud (MINSA), Caja de Seguro Social e Instituto Conmemorativo Gorgas de Estudios de la Salud, 2015 | Guía para el abordaje integral de la leishmaniasis en Panamá | Panama |
| 116 | Ministerio de Salud (MINSA), 2025 | Guía de Abordaje Clínico, Diagnóstico y Tratamiento de la Miasis | Panama |
| 117 | Dirección de Planificación, Departamento de Planificación de Salud Poblacional, Ministerio de Salud (MINSA), 2021 | Hacia el 2030: Enfermedades Infecciosas. Meta e Indicadores | Panama |
| 118 | Ministerio de Salud (MINSA), Instituto Conmemorativo Gorgas de Estudios de la Salud (ICGES), Caja de Seguro Social, 2019 | Guía para el Abordaje Clínico y Epidemiológico de las Rickettsiosis en Panamá | Panama |
| 119 | Asamblea Nacional de Panamá, 2022 | Ley N° 322 de 2022: Atención integral de la psoriasis | Panama |
| 120 | Ministerio de Salud (MINSA), 2025 | Decreto Ejecutivo N° 23 de 2025: Reglamenta la Ley 322 sobre psoriasis | Panama |
| 121 | Dirección General de Vigilancia de la Salud (DGVS), Ministerio de Salud Pública y Bienestar Social (MSPBS), 2022 | Guía Nacional de Vigilancia y Control de Enfermedades | Paraguay |
| 122 | Ministerio de Salud Pública y Bienestar Social (MSPBS), Dirección General de Vigilancia de la Salud (DGVS), 2021 | Guía de Tratamiento Antibiótico Empírico de infecciones más frecuentes adquiridas en la comunidad | Paraguay |
| 123 | Ministerio de Salud Pública y Bienestar Social (MSPBS), Programa Nacional de Control de VIH/SIDA/ITS (PRONASIDA), 2011 | Guía de Profilaxis y Tratamiento de las Infecciones Oportunistas en las PVVS | Paraguay |
| 124 | Ministerio de Salud Pública y Bienestar Social (MSPBS), Dirección General de Vigilancia de la Salud (DGVS), 2025 | Guía Nacional de Salud Pública para la Prevención y Control - Viruela Símica - Mpox. | Paraguay |
| 125 | Ministerio de Salud Pública y Bienestar Social (MSPBS), 2022 | Manual de Normas y Procedimientos del Programa Nacional de Control de Lepra | Paraguay |
| 126 | Ministerio de Salud Pública y Bienestar Social (MSPBS), 2018 | Manual de Diagnóstico y Tratamiento de las Leishmaniasis | Paraguay |
| 127 | Instituto de Medicina Tropical (IMT), Ministerio de Salud Pública y Bienestar Social (MSPBS), 2022 | Guía de Manejo en Pacientes con Viruela Símica Pediátricos y Adultos del I.M.T. | Paraguay |
| 128 | Ministerio de Salud Pública y Bienestar Social Paraguay, 2017 | Normas Nacionales de Vacunación, Técnico Administrativas y de Vigilancia del Programa Nacional de Enfermedades Inmunoprevenibles (PAI) | Paraguay |
| 129 | Comisión legislativa/Congreso Paraguay, 2025 | Proyecto de Ley: “Que crea el Programa Nacional de Prevención, Diagnóstico y Tratamiento de Psoriasis en Paraguay”. | Paraguay |
| 130 | Ministerio de Salud (MINSA), 2025 | Plan Nacional de Vacunación contra el Virus del Papiloma Humano para el Año Fiscal 2025 | Peru |
| 131 | Ministerio de Salud (MINSA), 2024 | Plan de preparación y respuesta ante el riesgo de introducción y propagación del nuevo clado de viruela símica (MPOX) en el Perú | Peru |
| 132 | Ministerio de Salud (MINSA), 2012 | Directiva Sanitaria para la Vigilancia Epidemiológica de Sarampión, Rubéola y otras Enfermedades Febriles Eruptivas | Peru |
| 133 | Ministerio de Salud (MINSA), 2022 | Norma Técnica de Salud para la vigilancia epidemiológica de viruela del mono (Mpox) | Peru |
| 134 | Ministerio de Salud (MINSA), 2025 | Norma Técnica de Salud para la Vigilancia Epidemiológica de Leishmaniasis en el Perú | Peru |
| 135 | Ministerio de Salud (MINSA), 2019 | Norma Técnica de Salud para la Prevención, Atención Integral y Control de la Lepra en el Perú | Peru |
| 136 | Senado de la República Dominicana, 2020 | Proyecto de Ley que agrega al catálogo de enfermedades catastróficas del Seguro Familiar de Salud la Artritis Reumatoide, la Esclerosis Múltiple y el Lupus Eritematoso Sistémico | Dominican Republic |
| 137 | Ministerio de Salud Pública y Asistencia Social (MISPAS), 2019 | Protocolo Vigilancia Sarampión | Dominican Republic |
| 138 | Ministerio de Salud Pública (MSP), 2025 | Protocolo para la profilaxis post exposición frente al virus varicela zóster | Uruguay |
| 139 | Ministerio de Salud Pública (MSP), 2022 | Guía de vigilancia 2022 Viruela símica | Uruguay |
| 140 | Ministerio de Salud Pública (MSP), 2015 | Guía Nacional de Vigilancia y Control de Enfermedades y Eventos Sanitarios de Notificación Obligatoria | Uruguay |

**List of excluded studies - electronic databases**

| **No.** | **Author, year** | **Title** | **Reason for exclusion** |
| --- | --- | --- | --- |
| 1 | Abrahams, 2016 | Resident-driven teledermatology in haiti: A symbiotic partnership in dermatologic education and health | Not a programme, public policy or strategy |
| 2 | Acurcio, 2021 | Cost-minimization analysis of teledermatology versus conventional care in the Brazilian National Health System | Preliminary results |
| 3 | Agostoni, 2015 | Control, containment and health education in the smallpox-vaccination campaigns in Mexico in the 1940s | Programme date |
| 4 | Alcala, 2025 | Latin America Cutaneous Oncology Management (LACOM) I: The Role of Skin Care in Oncology Patients and Survivors | Not a programme, public policy or strategy |
| 5 | Ali, 2024 | 53884 Teledermatology in Rural Guatemala - A Pilot Study | Not a programme, public policy or strategy |
| 6 | Aranda, 2015 | Economic, public health, and humanistic impact of a quadrivalent human papillomavirus (HPV6/11/16/18) vaccination program for females and males age 9-11 years in Mexico | Not a programme, public policy or strategy |
| 7 | Armada, 2021 | Estrategias digitales de educación en salud sobre leishmaniasis | Not a programme, public policy or strategy |
| 8 | Astley, 2021 | Home-based exercise program for adolescents with juvenile dermatomyositis quarantined during COVID-19 pandemic: a mixed methods study | Not a programme, public policy or strategy |
| 9 | Barrenechea, 2019 | Evaluation of impact of one dose varicella vaccine on the incidence of chickenpox in Argentina | Preliminary results |
| 10 | Barrera, 2024 | Comparison of Costs in Teledermatology Using PC and Camera Versus Smartphone | Not a programme, public policy or strategy |
| 11 | Batista, 2019 | Incremental Public Health and Economic Impact Linked to Higher Vaccine Coverage Rate in a Gender-Neutral Quadrivalent Human Papillomavirus Vaccination Program in Brazil | Not a programme, public policy or strategy |
| 12 | Beldarraín, 2017 | Historical overview of leprosy control in Cuba | Not a programme, public policy or strategy |
| 13 | Bernardes, 2021 | Active search strategies, clinicoimmunobiological determinants and training for implementation research confirm hidden endemic leprosy in inner São Paulo, Brazil | Not a programme, public policy or strategy |
| 14 | Bobbs, 2015 | Developing a low cost, mutually beneficial teledermatology collaboration | Not a programme, public policy or strategy |
| 15 | Borba, 2015 | Vigilância epidemiológica da hanseníase na atenção básica: o caso do município de Itaboraí, região metropolitana do Rio de Janeiro | Full text not available |
| 16 | Braga, 2025 | Global Perspectives on HPV Vaccination: Achievements, Challenges, and Lessons from the Brazilian Experience | No outcome |
| 17 | da Silva-Brandão, 2018 | Nursing care to comfort people with immunobullous dermatoses: Evaluation by fuzzy logic | Not a programme, public policy or strategy |
| 18 | Brito, 2025 | Artificial intelligence for the diagnosis of erythematous-squamous dermatological diseases: technological contributions to primary care | Not a programme, public policy or strategy |
| 19 | Castaneda-Rodríguez, 2024 | Efficacy of a burn prevention educational program to diminish the incidence of burn injuries in the pediatric population | Population type |
| 20 | Castillo, 2023 | Randomized trial evaluating an mHealth intervention for the early community-based detection and follow-up of cutaneous leishmaniasis in rural Colombia | Not a programme, public policy or strategy |
| 21 | Castro, 2024 | Effectiveness of Antileishmanial Treatments in Three Endemic Areas of Colombia: A Community-Based Cohort Study | Not a programme, public policy or strategy |
| 22 | Chouela, 2016 | Psoriasis in Latin America | Not a programme, public policy or strategy |
| 23 | Cid, 2016 | Teledermatología: experiencia en un centro de salud familiar urbano chileno | Not a programme, public policy or strategy |
| 24 | Cohn, 2019 | Gender equity in mass drug administration for neglected tropical diseases: Data from 16 countries | Not a programme, public policy or strategy |
| 25 | Cooper, 2022 | Yaws elimination in Ecuador: Findings of a serological survey of children in Esmeraldas province to evaluate interruption of transmission | Not a programme, public policy or strategy |
| 26 | Cossio, 2023 | mHealth Monitoring of Treatment of Cutaneous Leishmaniasis Patients: A Community-Based Implementation Study | Not a programme, public policy or strategy |
| 27 | Cotton, 2020 | Virtual Pediatric Emergency Department Telehealth Network Program: A Case Series | Population type |
| 28 | Cunico, 2023 | Initiatives to promote access to medicines after publication of the Brazilian Policy on the Comprehensive Care of People with Rare Diseases | Population type |
| 29 | Mazzuoccolo, 2021 | High Impact of a National Psoriasis Telementoring Clinic on Medical Practices for Patients in Underserved Areas | Duplicate |
| 30 | Cutler, 2019 | Teledermatology: Meeting the Need for Specialized Care in Rural Haiti | Not a programme, public policy or strategy |
| 31 | SouzadaCunha, 2015 | Chemoprophylaxis to control leprosy and the perspective of its implementation in brazil: A primer for non-epidemiologists | No outcome |
| 32 | Dabanch, 2019 | Chile's National Advisory Committee on Immunization (CAVEI): Evidence-based recommendations for public policy decision-making on vaccines and immunization | Population type |
| 33 | Dainton, 2017 | A narrative review of dermatologic protocols for primary care medical service trips in Latin America and the Caribbean | Not a programme, public policy or strategy |
| 34 | D´Avila, 2021 | Use of Health Services and Family Health Strategy Households Population Coverage in Brazil | Population type |
| 35 | de Carvalho, 2021 | Basic knowledge about visceral leishmaniasis before and after educational intervention among primary health care professionals in midwestern Brazil | Population type |
| 36 | de Souza, 2020 | Leprosy Screening Based on Artificial Intelligence: Development of a Cross-Platform App | Not a programme, public policy or strategy |
| 37 | Debernardi, 2020 | Teledermatology during de covid-19 pandemic in a public hospital | Not a programme, public policy or strategy |
| 38 | Denninger, 2022 | The rapid implementation of a pharmacy student vaccination program with the Department of Health to increase the delivery of intradermal monkeypox vaccinations | Population type |
| 39 | LopezDiNoto, 2026 | Real-world vitiligo management in Argentina: Bridging guidelines and practice | Not a programme, public policy or strategy |
| 40 | Diaz-Brochero, 2024 | Epidemiological findings, estimates of the instantaneous reproduction number, and control strategies of the first Mpox outbreak in Latin America | Not a programme, public policy or strategy |
| 41 | Echeverria, 2024 | Nine years of Project ECHO Psoriasis Argentina: All teach, all learn | Preliminary results |
| 42 | Edward, 2025 | Leprosy elimination: are we winning the fight or losing focus? | Not a programme, public policy or strategy |
| 43 | Eid, 2018 | Assessment of a leishmaniasis reporting system in tropical Bolivia using the capture-recapture method | Not a programme, public policy or strategy |
| 44 | Estrada-Castañon, 2015 | Qué resulta de cada jornada dermatológica en las comunidades | Not a programme, public policy or strategy |
| 45 | Ferreira, 2023 | Leprosy and tuberculosis control scenario of the national program for the improvement of access and quality of primary care in Brazil | Not a programme, public policy or strategy |
| 46 | Williams-Fonseca, 2015 | Project REENCONTRO: ethical aspects of genetic identification in families separated by the compulsory isolation of leprosy patients in Brazil | Not a programme, public policy or strategy |
| 47 | Fuentes-Silva, 2019 | Let's talk about lupus live video chats with an expert: An interactive facebook resource for patient education about lupus | Duplicate |
| 48 | de Oliveira, 2018 | Eutanásia canina como medida profilática para o controle da leishmaniose humana: uma abordagem bioética | Population type |
| 49 | Giavina-Bianchi, 2022 | Teledermatology in São Paulo, Brazil | Not a programme, public policy or strategy |
| 50 | Giavina-Bianchi, 2020 | Part II: Accuracy of Teledermatology in Skin Neoplasms | Not a programme, public policy or strategy |
| 51 | Giavina-Bianchi, 2019 | The majority of skin lesions in pediatric primary care attention could be managed by Teledermatology | Not a programme, public policy or strategy |
| 52 | Giavina-Bianchi, 2021 | Dermatologists’ perceptions on the utility and limitations of teledermatology after examining 55,000 lesions | Not a programme, public policy or strategy |
| 53 | Giavina-Bianchi, 2020-2 | Part I: Accuracy of Teledermatology in Inflammatory Dermatoses | Not a programme, public policy or strategy |
| 54 | Gilca, 2018 | Early use of the HPV 2-dose vaccination schedule: Leveraging evidence to support policy for accelerated impact | No outcome |
| 55 | Gomez, 2022 | Cost-effectiveness of Artificial Intelligence as a Decision-Support System Applied to the Detection and Grading of Melanoma, Dental Caries, and Diabetic Retinopathy | Not a programme, public policy or strategy |
| 56 | Graham, 2020 | Budget impact analysis of multiple varicella vaccination strategies: a Mexico perspective | Not a programme, public policy or strategy |
| 57 | Grayson, 2021 | 28666 Global initiatives in dermatology and education | Not a programme, public policy or strategy |
| 58 | Greisman, 2014 | Feasibility and cost of a medical student proxy-based mobile teledermatology consult service with Kisoro, Uganda, and Lake Atitlán, Guatemala | Not a programme, public policy or strategy |
| 59 | Guevara, 2018 | Elimination of onchocerciasis in Ecuador: findings of post-treatment surveillance | Not a programme, public policy or strategy |
| 60 | Henschel, 2021 | Leishmaniose visceral no estado de São Paulo, Brasil: avaliação das ações de vigilância e controle no Centro-Oeste Paulista, de 1999 a 2018, região endêmica para Leishmaniose Tegumentar | Population type |
| 61 | Hernandez-Zambrano, 2021 | Unexpected difference in acceptance of teleconsultation between patients with lupus and rheumatoid arthritis who underwent to a developed and implemented telemedicine innovative program after the declaration of quarantine due to the COVID-19 pandemic in Colombia | Not a programme, public policy or strategy |
| 62 | Insuasty, 2020 | Sistematización del programa de Teledermatología de la Universidad de Caldas - Departamento de Caldas, 2002-2010 | Not a programme, public policy or strategy |
| 63 | Insuasty, 2020 | Systematization Of The Teledermatology Program At The Universidad De Caldas-Department Of Caldas, 2002-2010 | Duplicate |
| 64 | Jalil, 2024 | EE244 Estimating Treatment Cost Savings of Gender-Neutral Nonavalent HPV Vaccination in Mexico | Not a programme, public policy or strategy |
| 65 | Jiménez-Quiñones, 2017 | Impact of a pharmacistconducted educational program on human papillomavirus vaccination rates in a low socioeconomic population in the City of Lares, Puerto Rico | Not a programme, public policy or strategy |
| 66 | Klarman, 2023 | Development And Evaluation Of Motomeds: A Pediatric Telemedicine And Medication Delivery Service For Resource-Limited Settings | Population type |
| 67 | Kolkhir, 2018 | Management of chronic spontaneous urticaria: a worldwide perspective | Not a programme, public policy or strategy |
| 68 | Kramer, 2020 | Oral health (dental treatment in epidermolysis bullosa) | Population type |
| 69 | Krisher, 2016 | Improving access to education and support for community health nurses in rural Guatemala through telehealth | Population type |
| 70 | Lang, 2024 | EE465 Modeling the Effects of Improving Varicella Vaccination Coverage Rates on Clinical and Economic Outcomes in Peru Over a 10-Year Time-Horizon | Not a programme, public policy or strategy |
| 71 | de Carvalho, 2024 | Automated Identification of Cutaneous Leishmaniasis Lesions Using Deep-Learning-Based Artificial Intelligence | Not a programme, public policy or strategy |
| 72 | Lima, 2023 | First Brazilian External Quality Assessment Program for Monkeypox Molecular Tests: Results from Two Surveys | Not a programme, public policy or strategy |
| 73 | Loescher, 2017 | Implementation of Project Students Are Sun Safe (SASS) in Rural High Schools Along the Arizona-Mexico Border | Population type |
| 74 | Lopes, 2021 | Leprosy in the context of the Family Health Strategy in an endemic scenario in Maranhão: prevalence and associated factors | No outcome |
| 75 | Luna, 2022 | Evaluation of the implementation of ECHO project in atopic dermatitis in Argentina: An innovative strategy to reach underserved areas with up-to-date knowledge | Duplicate |
| 76 | Luz, 2016 | Analysis of effectiveness of a surgical treatment algorithm for basal cell carcinoma | Not a programme, public policy or strategy |
| 77 | Luz, 2015 | Disease surveillance and patient care in remote regions: an exploratory study of collaboration among health-care professionals in Amazonia | Not a programme, public policy or strategy |
| 78 | Macedo, 2025 | Brazilian task force for the management of mucormycosis | Population type |
| 79 | Mancera-Cuevas, 2016 | A community-academic partnership educating hispanics/latinos as popular opinion leaders (pols) to increase awareness about lupus | Not from Latin America |
| 80 | Marks, 2024 | A pathway for skin NTD diagnostic development | No outcome |
| 81 | Martínez-Ripoll, 2026 | The segmentation of the Argentine healthcare system in the care of patients with epidermolysis bullosa: challenges and proposals for a comprehensive model | Population type |
| 82 | Maspero, 2017 | Translating chronic urticarial guidelines to clinical practice: A study assessing how allergists and dermatologists apply guidelines recommendations in Argentina | Not a programme, public policy or strategy |
| 83 | Mayre, 2020 | Developing evidence-based infographics for epidermolysis bullosa care in low-resource settings | Population type |
| 84 | Mena, 2022 | Demographics and distribution of dermatologists in Chile | Not a programme, public policy or strategy |
| 85 | Menezes, 2022 | Atraso na vacina tetravalente (DTP+ Hib) em crianças de 12 a 23 meses de idade: Pesquisa Nacional de Saúde, 2013 | Population type |
| 86 | Miranda, 2016 | Use of the Brazilian people's pharmacy program by older adults | Population type |
| 87 | Monsanto, 2017 | The implications of vaccine characteristics and private-sector vaccination on varicella | No outcome |
| 88 | Montoya, 2023 | Therapeutic response and safety of different treatments for cutaneous leishmaniasis in patients: A retrospective cross-sectional study | Not a programme, public policy or strategy |
| 89 | Mora, 2018 | Effect of an educational intervention based on clinical simulation in the diagnosis of psoriatic arthritis in latin american dermatologists | Not a programme, public policy or strategy |
| 90 | Mota, 2015 | Produção científica sobre teledermatologia nas bases de dados Lilacs, Medline, Scielo e Dynamed | No outcome |
| 91 | Munhoz, 2021 | Merkel cell carcinoma in Latin America: a contribution from an expanded access program for avelumab to address issues from experts’ recommendations | Not a programme, public policy or strategy |
| 92 | Navarrete, 2021 | Creation of a pioneering interdisciplinary genital dermatology unit for Hispanic men: First 269 patients | Not a programme, public policy or strategy |
| 93 | Navarrete, 2022 | Teledermatology and Artificial Intelligence | Not a programme, public policy or strategy |
| 94 | Nogueira, 2021 | Construction of the Care Network for Skin Lesions | No outcome |
| 95 | Orengo, 2023 | EPH100 Public Health Impact of Switching from a 4-Valent to a 9-Valent HPV Vaccination Program in Mexico | No outcome |
| 96 | Orengo, 2025a | Real-world Impact of Quadrivalent Human papillomavuris vaccination on Genital Warts and High-grade Cervical Intraepitelial Neoplasia Hospitalization rates in Brazil | Summary of included publication |
| 97 | Orengo, 2025b | Assessing the Public Health Impact of Transitioning from a 4-valent to a 9-valent HPV Vaccination Program in Panama | No outcome |
| 98 | Orengo, 2025c | Public Health and Economic Impact of Implementing Gender-Neutral Vaccination with the 4-Valent Human Papillomavirus Vaccine in Costa Rica | No outcome |
| 99 | Ortiz, 2017 | Modelling the effects of quadrivalent Human Papillomavirus (HPV) vaccination in Puerto Rico | No outcome |
| 100 | Oviedo, 2021 | Data management plan for a community-level study of the hidden burden of cutaneous leishmaniasis in Colombia | Not a programme, public policy or strategy |
| 101 | Palit, 2020 | Prevention of transmission of leprosy: The current scenario | No outcome |
| 102 | Romero-Palmera, 2016 | Factors Associated with the Registration and Reporting Leishmaniasis Control in Venezuela | Duplicate |
| 103 | Pedrozo, 2018 | Vigilancia intensificada de eventos de salud pública en comunidades desplazadas por inundaciones en Paraguay, 2015-2016 | No outcome |
| 104 | Perez, 2023 | Dermoscopy in Selected Latin American Countries: A Preliminary Look into Current Trends and Future Opportunities Among Dermatology Residency Programs | Not a programme, public policy or strategy |
| 105 | Pineda, 2018 | The cost-effectiveness of strategies to vaccinate against varicella in Mexico | Full text not available |
| 106 | Perez, 2024 | Use and legislation of Glyphosate in Mexico: healthcare, healthy environment and drinking water access | Not a programme, public policy or strategy |
| 107 | Pineda, 2018 | Impact of a tight control multidisciplinary rheumatology program on the quality of life of patients with autoinmune diseases in Colombia | Not a programme, public policy or strategy |
| 108 | Posso, 2024 | Sunscreens as a cost-effective measure in public health and its regulatory context in Colombia | Not a programme, public policy or strategy |
| 109 | Ramos, 2020 | A conditional cash transfer programme in Brazil improves leprosy treatment outcomes | Summary of included publication |
| 110 | Rendon, 2022 | EE329 Public Health and Economic Impact of a Gender-Neutral Quadrivalent Human Papillomavirus Vaccination Program in El Salvador | No outcome |
| 111 | Rendon, 2019 | Public Health and Economic Impact of A Human Papillomavirus Vaccination Program for Females Aged 9 Years in El Salvador | No outcome |
| 112 | Rey-Velasco, 2019 | PIN124 Impact of Chickenpox Vaccination in Colombia 2014-2017 | Summary of included publication |
| 113 | Ribeiro, 2024 | Safety profile of miltefosine in the treatment of cutaneous leishmaniasis | Not a programme, public policy or strategy |
| 114 | Richardus, 2019 | Feasibility and impact of leprosy post-exposure prophylaxis: Evidence from LPEP, a multi-country, 5-year implementation research program | Summary of included publication |
| 115 | Ritschl, 2019 | The need for personalized, non-pharmacological intervention programmes in autoimmune connective tissue disorders: Results of a EULAR-Funded Scoping Review with a Nested, Descriptive Meta-Analysis | Non-population-based |
| 116 | Rocha, 2022 | Impact of vector control actions in the abundance of Lutzomyia longipalpis in Montes Claros, Brazil | Population type |
| 117 | Venegas-Rodriguez, 2020 | Papillomavirus vaccine in Peru | No outcome |
| 118 | Rodriguez, 2023 | Leprosy Reactions: Experience in the Puerto Rico Hansen's Disease Population | Not a programme, public policy or strategy |
| 119 | Roldos, 2015 | Cost-effectiveness of quadrivalent human papilloma virus (HPV6/11/16/18) vaccination in Ecuador | No outcome |
| 120 | Romero-Palmera, 2016 | Factores asociados al registro y notificaciÃ³n de casos de leishmaniasis para el control en Venezuela | No outcome |
| 121 | RuizFuentes, 2019 | Leprosy in children: A Cuban experience on leprosy control | No outcome |
| 122 | Sacramento, 2019 | Chronicle of a consolidated success: smallpox eradication policies in Brazil (1962-1973) | Programme date |
| 123 | Saenz, 2018b | Skinhealth, a mobile application for supporting teledermatology: A case study in a rural area in Colombia | No outcome |
| 124 | Saenz, 2018 | On Using a Mobile Application to Support Teledermatology: A Case Study in an Underprivileged Area in Colombia | No outcome |
| 125 | Salvador, 2025 | Telemedicine in the clinical care of Chagas disease and American cutaneous leishmaniasis: pilot study in a public referral hospital in Brazil | Non-population-based |
| 126 | Salvador, 2025b | Telemedicine in the clinical care of neglected tropical diseases: A scoping review | Not a programme, public policy or strategy |
| 127 | Sanchez, 2023 | Healthcare Disparities in Atopic Dermatitis in Latin America: A Narrative Review | Not a programme, public policy or strategy |
| 128 | Sanchez, 2020 | Colombian Contributions Fighting Leishmaniasis: A Systematic Review on Antileishmanials Combined with Chemoinformatics Analysis | Not a programme, public policy or strategy |
| 129 | Sangers, 2024 | Position statement of the EADV artificial intelligence (AI) task force on AI‐assisted smartphone apps and web‐based services for skin disease | Not a programme, public policy or strategy |
| 130 | Santana, 2025 | Economic Evaluations and Equity in the Use of Artificial Intelligence in Imaging Examinations for Medical Diagnosis in People With Dermatological, Neurological, and Pulmonary Diseases: Systematic Review | Not a programme, public policy or strategy |
| 131 | Santos, 2018 | Vacinação pública contra o papilomavírus humano no Brasil | No outcome |
| 132 | Sepulveda, 2025 | AI-Powered Fast Screening for Neoplastic Diseases in the Chilean Population | Not a programme, public policy or strategy |
| 133 | Silva, 2023 | Occurrence of Tungiasis in Ceará State, Northeast Brazil: results of a rapid assessment method | Not a programme, public policy or strategy |
| 134 | Silva, 2024 | Human scabies and sarcoptic mange in northeast Brazil: Results from a rapid assessment method covering an entire state | Not a programme, public policy or strategy |
| 135 | Silveira, 2016 | Importance of the patient's clinical questionnaire for the diagnosis of skin cancer through teledermatology in remote areas of Brazil | Not a programme, public policy or strategy |
| 136 | Silveira, 2019 | Analysis of a decade of skin cancer prevention using a mobile unit in Brazil | No outcome |
| 137 | Smith, 2015 | Use of approximate Bayesian computation to assess and fit models of mycobacterium leprae to predict outcomes of the brazilian control program | Not a programme, public policy or strategy |
| 138 | Vieira-deSouza, 2015 | Health education in South America recarding leishmaniasis: a systematic review | Not a programme, public policy or strategy |
| 139 | Sousa, 2021 | ICNP® nursing diagnostics for people in-home care | Not a programme, public policy or strategy |
| 140 | Steinmann, 2018 | The Leprosy Post-Exposure Prophylaxis (LPEP) programme: Update and interim analysis | Preliminary results |
| 141 | Tamburus, 2025 | Diagnostic concordance challenges in dermatology: an internal analysis | Not a programme, public policy or strategy |
| 142 | Tejada, 2022 | Cost-effectiveness of human papillomavirus vaccination in girls living in Latin American countries: A systematic review and meta-analysis | No outcome |
| 143 | Tipan, 2026 | Impact of the teledermatology pilot program at Hospital General Docente Calderón in Ecuador | Non-population-based |
| 144 | Torres, 2018 | The benefits and costs of different scenarios for varicella vaccination in Chile | No outcome |
| 145 | Tropez, 2017 | The Psychological Impact of First Burn Camp in Nicaragua | Population type |
| 146 | Valipour, 2020 | Interventions for mycosis fungoides | Not a programme, public policy or strategy |
| 147 | Velazquez, 2017 | Persistence of trypanosoma cruzi DNA copies by quantitative real time PCR 12 months after treatment with benznidazole among children aged 4-15 years old in Bolivia | Full text not available |
| 148 | Velazquez, 2021 | Genodermatoses in Las Tunas Province, Cuba, 1989-2019 | Population type |
| 149 | Vlassoff, 2023 | Ensuring access to essential health products: Lessons from Colombia's leishmaniasis control and elimination initiative | No outcome |
| 150 | Williams, 2020 | 14234 Skin cancer and dermoscopy training for primary care physicians in Colombia: Effect on skin cancer diagnosis | Non-population-based |
| 151 | Wolfson, 2019 | Varicella healthcare resource utilization in middle income countries: a pooled analysis of the multi-country MARVEL study in Latin America & Europe | Not a programme, public policy or strategy |
| 152 | Yarak, 2017 | A Teledermatologia na Prática Médica | Not a programme, public policy or strategy |
| 153 | Zoni, 2019 | Epidemiological situation of yaws in the Americas: A systematic review in the context of a regional elimination goal | Population type |

TABLE S4 Risk of bias assessment of the included studies

**JBI Critical Appraisal Checklist for Prevalence Studies**

| **Author, year** | **1** | **2** | **3** | **4** | **5** | **6** | **7** | **8** | **9** | **Overall assessment** | **Rationale/Comments** |
| --- | --- | --- | --- | --- | --- | --- | --- | --- | --- | --- | --- |
| Grana, 2025 | Yes | Yes | Yes | No | Yes | Yes | Yes | Yes | N/A | 7/8 | Sociodemographic characteristics of the program participants were not reported |
| Mauro, 2019 | Yes | Yes | Yes | No | No | Yes | Yes | Yes | N/A | 6/8 | Participants’ characteristics were not reported; data were obtained from the surveillance system. Limitations due to underreporting were noted, particularly for mild adverse events and missing records. |
| Medeiros, 2024 | Yes | Yes | Yes | No | Yes | Yes | Yes | Yes | N/A | 7/8 | Participants’ characteristics were not described, and it is unclear whether data availability limitations influenced the selection of included records. |
| Richards, 2016 | No | Yes | Yes | No | UC | N/A | Yes | Yes | Yes | 5/8 | The sampling frame may not represent the entire endemic population. A bias toward higher awareness was acknowledged due to sentinel communities with greater contact with MSPAS brigades than other communities in the Central Endemic Zone. No participants’ characteristics were reported. |
| Richardus, 2021 | Yes | Yes | Yes | Yes | Yes | Yes | Yes | Yes | Yes | 9/9 |  |
| Romanholo, 2018 | Yes | UC | UC | No | Yes | Yes | UC | Yes | No | 4/9 | Recruitment depended on index case authorization, location, and willingness to participate. Participants’ sociodemographic characteristics were not reported. A standardized interview was used. Data were self-reported, with potential recall bias. |
| Vitiritti, 2024 | Yes | Yes | N/A | Yes | Yes | Yes | Yes | Yes | N/A | 7/7 |  |
| Franca, 2023 | Yes | Yes | Yes | Yes | Yes | Yes | Yes | Yes | N/A | 8/8 |  |
| Gentile, 2022 | No | Yes | Yes | No | Yes | Yes | Yes | Yes | N/A | 6/8 | The sample was drawn from a pediatric hospital, which may limit population representativeness; additional details were not described. |
| Botto 2016 | Yes | Yes | Yes | Yes | Yes | Yes | Yes | UC | Yes | 8/9 | The analysis plan is not clearly described. |
| **Items**   1. Was the sample frame appropriate to address the target population? 2. Were study participants sampled in an appropriate way? 3. Was the sample size adequate? 4. Were the study subjects and the setting described in detail? 5. Was the data analysis conducted with sufficient coverage of the identified sample? 6. Were valid methods used for the identification of the condition? 7. Was the condition measured in a standard, reliable way for all participants? 8. Was there appropriate statistical analysis? 9. Was the response rate adequate, and if not, was the low response rate managed appropriately?   **Note**: Falleiros-Arlant (2017), Poester (2022), and Pujadas-Ferrer (2023) are abstract-only publications; thus, there is insufficient information available to conduct the assessment.  N/A: not applicable; UC: unclear | | | | | | | | | | | |

**JBI Critical Appraisal Checklist for Cross-Sectional Studies**

| **Author, year** | **1** | **2** | **3** | **4** | **5** | **6** | **7** | **8** | **Overall assessment** | | **Rationale/Comments** | |  |
| --- | --- | --- | --- | --- | --- | --- | --- | --- | --- | --- | --- | --- | --- |
| Beltrán, 2016 | Yes | Yes | Yes | Yes | No | No | Yes | Yes | | 6/8 | | Limited control of confounding; small clinical subgroups (e.g., non-adherent patients). | |
| Mazzuoccolo, 2020 | Yes | UC | Yes | Yes | No | No | Yes | Yes | | 5/8 | | Lack of confounding control; missing description of study subjects. | |
| de Oliveira, 2021 | UC | UC | Yes | UC | No | No | UC | Yes | | 2/8 | | Lack of variable operationalization; absence of confounding control; predominantly narrative/descriptive approach. | |
| Fuenzalida, 2025 | Yes | UC | UC | UC | No | No | UC | UC | | 1/8 | | Lack of confounding control; limited detail in variable measurement. | |
| Gatica, 2023 | Yes | Yes | Yes | Yes | No | No | UC | No | | 4/8 | | No identification or control of confounding; lack of formal validation of quality indicators. | |
| Gatica, 2015 | Yes | Yes | Yes | No | No | No | UC | UC | | 3/8 | | Lack of rigorous methodological definition and absence of confounding control; predominantly descriptive analysis. | |
| González-Coloma, 2019 | Yes | UC | Yes | Yes | N/A | N/A | Yes | Yes | | 5/6 | | Sociodemographic characteristics of participants were not reported; possible limitation due to available records. | |
| Luna, 2022 | Yes | Yes | UC | N/A | N/A | N/A | Yes | UC | | 3/5 | | It is stated that a survey was conducted among project participants, but it is unclear whether the number of respondents corresponds to the total number of participants. Descriptive measures are not reported in detail. The instrument was not validated (survey). | |
| Manubens, 2023 | Yes | Yes | UC | N/A | N/A | N/A | Yes | UC | | 3/5 | | Response rate was considered insufficient (32.7%). The instrument was not validated (survey). Descriptive measures are not reported in detail. | |
| Mazzuoccolo, 2019 | Yes | UC | UC | N/A | N/A | N/A | Yes | Yes | | 3/5 | | Response rate was considered insufficient (63.7%). The instrument was not validated (survey); participants’ characteristics were not described. | |
| Messagier, 2019 | Yes | No | Yes | N/A | N/A | N/A | Yes | UC | | 3/5 | | All eligible cases were included; manual data extraction, limited participant characteristics, and system constraints restricted detailed assessment of MAST indicators | |
| Nunes, 2025 | Yes | Yes | Yes | N/A | N/A | N/A | Yes | Yes | | 5/5 | | Possible limitations were acknowledged regarding incomplete reporting of notifications. | |
| Wakiyama, 2017 | Yes | UC | Yes | Yes | UC | UC | Yes | Yes | | 5/8 | | Some participant characteristics were described. Possible retrospective measurement inaccuracies were acknowledged by the authors. Confounding factors were not identified. | |
| von Wangenheim, 2019 | Yes | UC | Yes | N/A | N/A | N/A | Yes | UC | | 3/5 | | Participants’ characteristics were not reported, and no explicit analysis plan was described. | |
| **Items**   1. Were the criteria for inclusion in the sample clearly defined? 2. Were the study subjects and the setting described in detail? 3. Was the exposure measured in a valid and reliable way? 4. Were objective, standard criteria used for measurement of the condition? 5. Were confounding factors identified? 6. Were strategies to deal with confounding factors stated? 7. Were the outcomes measured in a valid and reliable way? 8. Was appropriate statistical analysis used? | | | | | | | | | | | | | |

N/A: not applicable; UC: unclea

**JBI critical appraisal checklist for quasi-experimental studies**

| **Author, year** | **1** | **2** | **3** | **4** | **5** | **6** | **7** | **8** | **9** | **Overall assessment** |
| --- | --- | --- | --- | --- | --- | --- | --- | --- | --- | --- |
| **Giglio 2022** | Yes | Yes | Yes | UC | Yes | Yes | Yes | Yes | Yes | 10/10 |
| **Barrenechea 2025** | Yes | No | UC | UC | Yes | Yes | Yes | Yes | Yes | 7/10 |
| **de Andrade 2018** | Yes | Yes | Yes | UC | Yes | Yes | Yes | Yes | Yes | 10/10 |
| **Bierrenbach 2025** | Yes | No | UC | UC | Yes | UC | Yes | Yes | Yes | 7/10 |
| **Abreu e Silva 2022** | Yes | No | Yes | UC | Yes | Yes | Yes | Yes | Yes | 7/9 |
| **Miranda 2023** | Yes | No | Yes | UC | Yes | Yes | Yes | Yes | Yes | 7/9 |
| **Pawaskar 2022** | Yes | No | Yes | UC | Yes | Yes | Yes | Yes | Yes | 7/9 |
| **Ribeiro 2020** | Yes | No | Yes | UC | Yes | Yes | Yes | Yes | Yes | 7/9 |
| **Scotta 2018** | Yes | No | Yes | UC | Yes | Yes | Yes | Yes | Yes | 7/9 |
| **Rodrigues-Barbieri 2016** | Yes | No | UC | UC | Yes | Yes | Yes | Yes | Yes | 6/9 |
| **Criado 2023** | Yes | No | Yes | Yes | Yes | Yes | Yes | Yes | Yes | 8/9 |
| **de Abreu 2020** | Yes | No | Yes | UC | Yes | Yes | Yes | Yes | Yes | 7/9 |
| **Gentile 2021** | Yes | No | Yes | UC | Yes | Yes | Yes | Yes | Yes | 7/9 |
| **Items**:   1. Is it clear in the study what is the “cause” and what is the “effect” (i.e., there is no confusion about which variable comes first)? 2. Was there a control group? 3. Were the participants included in any comparisons similar? 4. Were the participants included in any comparisons receiving similar treatment/care, other than the exposure or intervention of interest? 5. Were there multiple measurements of the outcome both pre and post the intervention/exposure? 6. Were the outcomes of participants included in any comparisons measured in the same way? 7. Were outcomes measured in a reliable way? 8. Was follow-up complete and if not, were differences between groups in terms of their follow-up adequately described and analyzed? 9. Was appropriate statistical analysis used? | | | | | | | | | | |

N/A: not applicable; UC: unclear

**JBI critical appraisal checklist for cohort studies**

| **Author, year** | **1** | **2** | **3** | **4** | **5** | **6** | **7** | **8** | **9** | **10** | **11** | **Overall assessment** | **Rationale/Comments** |
| --- | --- | --- | --- | --- | --- | --- | --- | --- | --- | --- | --- | --- | --- |
| Lages, 2025 | N/A | Yes | Yes | UC | UC | N/A | Yes | Yes | N/A | N/A | Yes | 5/7 | Aggregate-level analysis using secondary population-based data; confounding control unclear. |
| Machado, 2022 | N/A | Yes | Yes | Yes | Yes | N/A | Yes | UC | N/A | N/A | Yes | 6/7 | Aggregate-level analysis using secondary population-based data; follow-up period (2016–2018) may be limited for some outcomes (e.g., spatial changes). |
| Pescarini, 2020 | Yes | Yes | Yes | Yes | Yes | Yes | Yes | Yes | No | N/A | Yes | 9/10 | Propensity score used; only complete records for outcomes were included. |
| Richards, 2015 | N/A | Yes | Yes | UC | UC | N/A | Yes | Yes | N/A | N/A | UC | 4/7 | Aggregate-level analysis using secondary population-based data; confounding control and use of inferential models not clearly reported. |
| Sánchez, 2017 | Yes | N/A | Yes | No | UC | Yes | Yes | Yes | Yes | No | Yes | 6/10 | Multivariable analysis performed, but confounders not specified; reasons for loss to follow-up reported. |
| de Melo, 2024 | Yes | Yes | UC | Yes | Yes | UC | Yes | Yes | UC | N/A | Yes | 7/10 | Limited confounding control; heterogeneous definition of policy implementation; imperfect comparability across states. |
| Teixeira, 2025 | Yes | Yes | UC | Yes | Yes | UC | Yes | Yes | Yes | N/A | Yes | 8/10 | Proxy exposure definition used; household exposure not ensured; incubation period variability and diagnosis timing may not reflect true infection time. |
| **Items**   1. Were the two groups similar and recruited from the same population? 2. Were the exposures measured similarly to assign people to both exposed and unexposed groups? 3. Was the exposure measured in a valid and reliable way? 4. Were confounding factors identified? 5. Were strategies to deal with confounding factors stated? 6. Were the groups/participants free of the outcome at the start of the study (or at the moment of exposure)? 7. Were the outcomes measured in a valid and reliable way? 8. Was the follow-up time reported and sufficient to be long enough for outcomes to occur? 9. Was follow-up complete, and if not, were the reasons for loss to follow-up described and explored? 10. Were strategies to address incomplete follow-up utilized? 11. Was appropriate statistical analysis used? | | | | | | | | | | | | | |

N/A: not applicable; UC: unclear

**JBI Critical Appraisal Checklist for Qualitative Studies**

| **Author, year** | **1** | **2** | **3** | **4** | **5** | **6** | **7** | **8** | **9** | **10** | **Overall assessment** | **Rationale/Comments** |
| --- | --- | --- | --- | --- | --- | --- | --- | --- | --- | --- | --- | --- |
| Martos-Casado, 2022 | UC | Yes | Yes | Yes | Yes | No | UC | Yes | Yes | Yes | 7/10 | The theoretical or philosophical framework is not clearly identified; researchers’ beliefs/values and reflexivity are not described, limiting reflexivity assessment. |
| Mendizábal, 2021 | UC | Yes | Yes | Yes | Yes | UC | Yes | Yes | Yes | Yes | 8/10 | The theoretical or philosophical framework is not clearly stated; researchers’ theoretical position is not explicit. Data from focus groups and interviews were complemented with document review; results were validated in separate meetings with providers and community representatives. |
| Morice, 2023 | UC | Yes | Yes | Yes | Yes | No | UC | No | UC | Yes | 5/10 | The theoretical or philosophical framework is not clearly identified; lack of reflexivity and absence of verbatim quotes or primary supporting data. Ethical approval is not reported, although verbal informed consent is mentioned. |
| do Nascimento, 2024 | Yes | Yes | Yes | Yes | Yes | No | No | UC | UC | UC | 5/10 | The theoretical or philosophical framework is not clearly identified; no verbatim quotes or primary data are reported. Ethical approval, participant representation, and linkage between analysis and conclusions are unclear. |
| **Items**   1. Is there congruity between the stated philosophical perspective and the research methodology? 2. Is there congruity between the research methodology and the research question or objectives? 3. Is there congruity between the research methodology and the methods used to collect data? 4. Is there congruity between the research methodology and the representation and analysis of data? 5. Is there congruity between the research methodology and the interpretation of results? 6. Is there a statement locating the researcher culturally or theoretically? 7. Is the influence of the researcher on the research, and vice-versa, addressed? 8. Are participants and their voices adequately represented? 9. Is the research ethical according to current criteria, and is there evidence of ethical approval by an appropriate body? 10. Do the conclusions drawn in the research report flow from the analysis or interpretation of the data? | | | | | | | | | | | | |

N/A: not applicable; UC: unclea

**ROBIS: Systematic Reviews of the Literature**

| **Domains / Author, year** | **Ahmed, 2022** | **Falleiros-Arlant, 2019** |
| --- | --- | --- |
| Domain 1: Eligibility criteria | Low | Low |
| Domain 2: Identification and selection | Low | Low |
| Domain 3: Data collection and appraisal | Low | High |
| Domain 4: Synthesis and findings | Low | Low |
| **Overall risk** | Low | Low |
| **Comments** | None | None |

**CHEERS Checklist for Economic Evaluations**

| **Item** | **Section** | **Description** | **Abi-Saab, 2021** | **da Costa, 2024** | **Giglio, 2023** |
| --- | --- | --- | --- | --- | --- |
| 1 | Title | Identify the study as an economic evaluation and specify the interventions being compared. | Yes | Yes | Yes |
| 2 | Abstract | Provide a structured summary including context, methods, results, and conclusions. | Yes | Yes | Yes |
| 3 | Background and objectives | Describe the study context, background, and objectives. | Yes | Yes | Yes |
| 4 | Health economic analysis plan | Indicate whether a health economic analysis plan was developed and where it can be accessed. | Yes | Yes | Yes |
| 5 | Study population | Describe characteristics of the study population and relevant subgroups. | Yes | No | No |
| 6 | Setting and location | Describe contextual factors relevant to the study setting and decision. | Yes | Yes | Yes |
| 7 | Comparators | Describe the interventions or strategies being compared. | Yes | Yes | Yes |
| 8 | Perspective | State the analytical perspective adopted. | Yes | Yes | Yes |
| 9 | Time horizon | Report the time horizon over which costs and outcomes were evaluated. | Yes | Yes | Yes |
| 10 | Discount rate | Report the discount rates applied to costs and outcomes. | No | Yes | Yes |
| 11 | Selection of outcomes | Describe the health outcomes included in the evaluation. | Yes | Yes | Yes |
| 12 | Measurement of outcomes | Describe how outcomes were measured or estimated. | Yes | Yes | Yes |
| 13 | Valuation of outcomes | Describe the methods used to value outcomes (e.g., preferences or utilities). | N/A | N/A | N/A |
| 14 | Measurement and valuation of resources and costs | Describe the resources used, unit costs, and methods for their valuation. | Yes | Yes | No |
| 15 | Currency, price date, and conversion | Report the currency, price year, and any inflation or currency conversion adjustments. | Yes | Yes | No |
| 16 | Rationale and description of model | Describe the model used and justify its selection (if applicable). | No | Yes | No |
| 17 | Analytics and assumptions | Describe analytical methods and structural assumptions. | No | Yes | No |
| 18 | Characterising heterogeneity | Describe methods used to assess heterogeneity or subgroup effects. | No | Yes | No |
| 19 | Characterising distributional effects | Describe distributional or equity impacts, if applicable. | No | Yes | Yes |
| 20 | Characterising uncertainty | Describe methods used to assess uncertainty, including sensitivity analyses. | No | Yes | No |
| 21 | Approach to engagement with patients and others affected by the study | Describe how patients, the public, or other stakeholders were involved, if applicable. | No | No | No |
| 22 | Study parameters | Report input parameter values, sources, and ranges. | No | Yes | Yes |
| 23 | Summary of main results | Present the main findings, including costs, outcomes, and incremental results where applicable. | No | Yes | Yes |
| 24 | Effect of uncertainty | Report the results of uncertainty or sensitivity analyses. | No | Yes | No |
| 25 | Effect of engagement with patients and others affected by the study | Report the influence of stakeholder engagement on the study, if applicable. | No | Yes | No |
| 26 | Discussion | Discuss the main findings, limitations, and implications. | Yes | Yes | No |
| 27 | Source of funding | Report funding sources and the role of the funder. | Yes | Yes | No |
| 28 | Conflicts of interest | Declare any conflicts of interest. | Yes | Yes | No |
| **Compliance (%)** | | | 59,3% | 92,6% | 51,9% |

**TABLE S5** Characteristics of Identified Tools for the Management of Dermatological Conditions

| **Implementation tool (intervention)** | **Available evidence** | **Dermatological disease** | **Implementation contexts**  Country \| geographic setting \| proposed level \| implementation level | **Outcomes** Study outcomes\| Findings | **Barriers** | **Facilitators** | **Implementation requirements/ recommendations** |
| --- | --- | --- | --- | --- | --- | --- | --- |
| **High disease burden and persistent transmission** | | | | | | | |
| Primary prevention based on vaccination programs | Guidelines, regulations, manuals, strategic plans; ecological analyses, cohort and cross-sectional studies | Varicella; measles; HPV warts; mpox | **Argentina, Brazil, Chile, Colombia, Costa Rica, Ecuador, El Salvador, Guatemala, Honduras, Mexico, Panama, Peru, Uruguay, LAC** At-risk and eligible groups \| Rural and urban areas \| Macro \| Community | **Clinical outcomes:** incidence, hospitalization, transmission \| reduction **Clinical outcomes:** HPV adverse events \| mild **System:** costs \| reduction **Implementation:** vaccination coverage \| increase | Coverage gaps  Operational and logistical challenges  Lack of standardization and interoperability of health information systems  COVID-19 pandemic impact | National immunization programs  Strengthening of primary health care (PHC)  Guidelines and technical manuals  High coverage systems  Standardized surveillance systems  Institutional support | Embed within comprehensive programmatic strategies  Ensure continuous monitoring and evaluation |
| Early detection and transmission control  (including contact tracing, screening, prophylaxis, surveillance of contacts, mass drug administration, parasitological/entomological/serological monitoring, vector control, diagnostic networks) | Regulations, guidelines, protocols and strategies; cross-sectional studies, cohort studies, economic evaluations | Leprosy; onchocerciasis; leishmaniasis; varicella | **Argentina, Brazil, El Salvador, Guatemala, Nicaragua, Uruguay, Venezuela**  At-risk populations \| Rural and urban areas \| Macro \| Community | **Clinical outcomes:** case detection \| improve **Clinical outcomes:** transmission indicators \| reduced **Implementation:** coverage \| increase | Geographic and logistical constraints  Variable quality of contact surveillance  Limited availability of trained personnel  Sociocultural factors (stigma, reluctance to disclose disease)  Mobility and insecurity  Drug availability issues  Environmental conditions | Integration into existing programs  Institutional and international support  Intersectoral coordination  Regulatory frameworks  Health workforce motivation  Family participation and education  Local adaptation | Sustained, territorially focused and context-adapted strategies  Continuous education and institutional presence  Strengthened health workforce, surveillance, laboratory capacity, medication supply chains, training and information systems |
| Strengthening health information systems and surveillance  (including integrated information systems, post-elimination surveillance, drug resistance surveillance) | Guidelines, manuals, protocols, national plans; reports; cross-sectional and cohort studies | Leishmaniasis; onchocerciasis; cysticercosis; measles; varicella; mpox; leprosy; myiasis; HPV; skin cancer | **Argentina, Bolivia, Brazil, Colombia, Costa Rica, Ecuador, El Salvador, Guatemala, Guyana, Honduras, Mexico, Nicaragua, Panama, Paraguay, Peru, Dominican Republic, Suriname, Uruguay, Venezuela** General population and eligible groups\| NS \| Macro \| Health system | **Implementation:** Feasibility \| yes **Implementation:** Performance \| variable **Clinical outcomes:** risk of relapse \| higher associated with drug resistance | Underreporting and delayed notification  Data quality issues  Technological limitations  Operational complexity and limited technical capacity  Weak laboratory capacity sustainability  Drug availability constraints  Stigma and misinformation | Standardization of indicators and procedures  Legal frameworks  Institutional and international support  Intersectoral coordination  Capacity strengthening  Community participation | Standardized integrated surveillance systems and strengthened laboratory capacity  Improved data quality and timeliness  Investment in infrastructure, HR, databases, training and coordination |
| Integrated control, surveillance and care strategies | Plans, programs, guidelines; qualitative reports and cohort studies | Leishmaniasis; leprosy; mpox; measles; rubella | **Argentina, Bolivia, Brazil, Colombia, Ecuador, El Salvador, Guatemala, Panama, Paraguay, Peru, Venezuela, LAC** General population, Health care providers \| NS \| Macro \| Health system | **Implementation:** degree of implementation \| variable **Implementation:** coverage \| increase **Clinical outcomes:** cure \| increase **Clinical outcomes:** treatment proportion \| partial **Clinical outcomes:** adherence \| variable | Lack of referral services, low problem-solving capacity, discontinuity of programs  Resource scarcity  Staff shortages  Poverty  Population mobility  Supply shortages  Access difficulties  Cultural factors (fear, shame, preference for traditional remedies)  Low health literacy, stigma | Institutional commitment  Intersectoral coordination  Strengthening local capacity  Community-based strategies  Legal frameworks and protocols | Integrated, sustainable, and primary health care–centered models  Cultural, community, and territorial approach  Emphasis on continuity of care  Strengthening of information systems  Incorporation of social determinants of health  Improved surveillance and communication |
| **Barriers to access to dermatological diagnosis and care** | | | | | | | |
| Teledermatology | Cross-sectional studies | Skin conditions; psoriasis | **Chile, Brazil, Argentina, French Guiana** Population seeking dermatology care; patients with psoriasis \| Remote/dispersed; rural; NS \| Macro; health system \| Primary care; specialized care; health system | **Implementation:**  Consultations, participation, coverage \| Goals achieved in consultations; high coverage and participation **Experience:**  Acceptability/adoption \| Adequate  Capacity building \| High  Satisfaction \| High and adequate among both professionals and patients  **Access:**  Response times \| Reduced **Care processes:**  Concordance \| High between in-person and virtual dermatologists, low between other health professionals and dermatologists **System (costs and efficiency):** Referral to in-person care \| Decreased Costs \| Economically dominant **Clinical outcomes:**  Conditions not suitable for TD \| STIs and genital conditions (4.2%), tumor-related (4.2%), pigmented disorders (2.1%), and oral pathology (0.7%) | Lack of specialists  Absence of physical and specialized examination  Audiovisual limitations and connectivity issues  Medicolegal concerns and privacy and confidentiality issues  Lack of patient follow-up and high turnover of medical staff | Proximity to health care centers and greater convenience and accessibility, especially for special populations such as pediatric patients  Availability of secure digital platforms  Moderate acceptance among dermatologists and patients  Educational value for health care staff  Network collaboration between levels of care | Strengthening digital infrastructure and connectivity  Training and increased specialist workforce  Standardized protocols  Integration into PHC networks |
| Community dermatology programs | Plan; Regulations; Cohort studies; Cross-sectional studies; Reports | Mycetoma; Leprosy; Skin conditions; Skin cancer; Psoriasis; SLE | **Brazil; Mexico; Argentina; Panama; Paraguay; Dominican Republic**  General population; primary health care users; health professionals \| Remote/dispersed; NS \| Macro \| Health system; community | **Experience:** Community outreach campaigns \| Conducted in rural areas twice a year and replicated in other provinces  **Implementation:** Program sustainability over time \| Active for 30 years; Achievements/partnerships \| International partnerships; centers in other countries; Medication demand \| Low proportion due to non-biologic therapies  **Clinical outcomes:** Detection and detection rates \| Decreasing, with cases detected at earlier stages | Heterogeneity in program coverage across regions  Poor transportation infrastructure; population mobility; socioeconomic conditions  Benefits of community programs are difficult to estimate  Lack of knowledge, training, and professionals  Lack of records and follow-up  High cost of treatments | Inclusion of medical students in community programs enables dermatology training  Community engagement through workshops for primary school children is cost-effective and rewarding  Existence of entities or structures that support the initiative  Institutional coordination | Design and implement short-, medium-, and long-term impact evaluation programs.  Develop scientific materials in simple, accessible language, free of formal jargon.  Promote interdisciplinary collaboration among all stakeholders to design appropriate educational strategies.  Ensure adequate human resources, infrastructure, technology (information and registry systems), funding (allocated state budget), and training.  Implement public policies. |
| National Skin Cancer Prevention Campaign | Cross-sectional studies | Skin cancer | **Brazil** Patients with basal cell carcinoma (BCC) \| NS \| Macro \| Community | **Clinical outcomes:** Basal cell carcinoma (BCC) tumor size \| Reduction (p<0.01) | NR | NR | Provide dermatology equipment, training, and an integrated network.  Expand public awareness of the disease, thereby reducing public health care costs.  Implement wider-coverage, mobile, and high-risk–targeted campaigns to optimize outcomes. |
| Referral protocol | Protocols; Cross-sectional studies | Leprosy; zoonotic sporotrichosis; rosacea, varicella, psoriasis, scabies, pediculosis, superficial mycoses; skin conditions | **Brazil; Chile** Patients with suspected/diagnosed cases; Rio de Janeiro and Mossoró \| NS \| Macro \| Health system | **Clinical outcomes:** Number of cases \| Reduction  Proportion of visits to a single clinic before referral \| Reduction  Detection of new cases \| Increase  Time to diagnosis \| Reduction | Difficulties in retaining physicians and nurses in Primary Health Units (UBS) and high staff turnover  Difficulties in securing sufficient financial resources to adequately train new professionals  Lack of knowledge among staff; delay in clinical suspicion  Geographical barriers; high demand and shortage of specialists | Health education; institutional coordination; free access to sporotrichosis care  Training; teledermatology; network coordination | Provide a specialized service, a mycology laboratory, and coordination with primary health care (PHC).  Monitor and address the increasing proportion of patients with other skin conditions.  Strengthen telemedicine platforms and expand technological infrastructure.  Increase availability of health care personnel. |
| Surveillance and reporting | Qualitative; Cross-sectional studies | Cutaneous leishmaniasis; leprosy | **Brazil**  Patients in the Brazilian Amazon/Caçador–Santa Catarina \| Remote/dispersed; NS \| Macro \| Health system; community | **Access:** Time to diagnosis \| Decrease (p<0.00001 **Implementation:** Identification of probable sites of infection \| Improvement **Clinical outcomes:** Cure in first treatment cycle \| Increase; Case detection \| Increase; Change in clinical classification standards and disability grading \| Reduction in grade 2 disability cases and in lepromatous patients | Cultural and geographic access barriers  Limitations of the official health system | Community participation; traditional knowledge; trained CHWs  PHC training | Integrate local knowledge into surveillance systems.  Decentralize diagnosis by training health workers in laboratory diagnostics and using alternative microscopy methods that do not require electricity.  Decentralize treatment delivery.  Use participatory methods.  Promote participatory monitoring with an intercultural approach. |
| Laboratory network | Cross-sectional studies | Cutaneous leishmaniasis | **Brazil** Patients in Minas Gerais \| NS \| Macro \| Health system | **Implementation:** Diagnostic coverage \| Increased; Adherence to the algorithm \| Very low | Diagnostic limitations; unequal access; incomplete adherence to the diagnostic algorithm | Existing infrastructure; GAL system; organized network | Expand the diagnostic network model and improve adherence to the algorithm |
| **Knowledge and training gaps** | | | | | | | |
| Digital training tools | Report | SLE | **LAC** Women who speak Spanish/Portuguese with SLE \| NS \| Intersectoral \| Community | **Experience:** Dissemination and participation on social media; user satisfaction \| High | It requires significant time commitment from production and community management teams, as well as multiple participants, to reach a broad audience. | Availability for live sessions with experts  Community management by a team of rheumatologists | Implement a low-budget program supported by small grants and in-kind contributions from stakeholders. |
| Comprehensive primary health care strategy | Report | Cutaneous leishmaniasis; Mpox | **Brazil** Health care professionals \| NS \| Health system \| Primary care | **Experience:** Usability and adherence; adoption \| Adequate | Resource-intensive in content creation and maintenance  Low participation from health service users  Limitations of the conventional training model  Limited capacity for program monitoring and evaluation | Existence of the PACK program  Flexible training curriculum  The comprehensive nature of PACK enabled the maintenance of routine primary health care (PHC) service delivery | An established primary health care program enables rapid deployment during outbreaks.  A familiar design improves usability.  An established and agile update mechanism allows for rapid changes.  It is necessary to explore methodologies that enable meaningful and rapid community participation in outbreak scenarios. |
| Self-care support groups for leprosy | Qualitative | Leprosy | **Brazil** People with leprosy \| NS \| Macro \| Specialized care | **Experience:** Implementation, organization, and continuity of self-care support groups \| Adequate | Service organization-related problems  Persistent disease-related stigma  Long-term retention of users, limiting the incorporation of new participants | Participation of professionals in group formation  Support from external stakeholders  Participation of health care teams | Health policies should support existing self-care support groups by providing subsidies and encouraging the creation of new groups.  The national leprosy control policy should include group formation as a core strategy. |
| PENN–INDERMA Dermatology Educational Exchange Program | Report | Multiple dermatoses (atopic dermatitis, scabies, verruca vulgaris, and acne) | **Guatemala** Indigenous population \| Rural \| Health system (services/institutional) \| Community | **Implementation:** Coverage \| Bidirectional rotations, consultations, and successful patient encounters | NR | International academic collaboration | Improve access to dermatology care  Develop a sustainable model  Build frameworks for local care delivery  Establish partnerships with long-term programs  Implement telemedicine |
| Educational Program: The Sun, a Friend of Childhood | Cross-sectional studies | Skin cancer | **Brazil** Children in primary education from public and private schools \| NS \| Macro \| Community | **Implementation:** Impact on children and parents \| Positive | NR | Promotional messages delivered by public figures and presented in simple formats are more likely to be well received by target audiences.  Campaigns targeting children should encourage parental involvement to maximize effectiveness and improve outcomes. | The program contributed to raising awareness among children, parents, and teachers regarding the importance of sun protection in skin cancer prevention.  Maintain and expand childhood sun protection education programs, incorporating behavioral, environmental, and policy strategies. |
| ECHO Psoriasis Project and ECHO Atopic Dermatitis Project | Cross-sectional studies | Psoriasis; Atopic Dermatitis | **Argentina** Population with psoriasis and atopic dermatitis \| Remote/dispersed \| Macro \| Specialized care | **Experience:**  Number of teleconsultations and participants \| High participation of dermatologists Recommendation to modify the therapeutic approach during teleconsultations \| 69.6%, including changes to ongoing treatment, confirmation of the current approach, and initiation of new treatment Adherence to the recommended change in therapeutic approach \| 66.4% Understanding and overall management of patients with atopic dermatitis \| Improved **Implementation:** Adoption \| Very high | Lack of adherence to treatment  Difficulties in accessing health services | Initiatives that are freely accessible and easy to implement and use are useful for supporting medical practice in patients with psoriasis.  Previous experiences (ECHO Psoriasis Project and ECHO Hidradenitis Project) achieved high levels of participation. | Through tele-mentoring, it is possible to mitigate the professional isolation experienced by nearly half of the dermatologists in the country.  It is recommended to strengthen distance medical education programs, expand tele-mentoring networks, and evaluate in future research their direct impact on quality of care and patient outcomes. |
| **Clinical variability and lack of standardization** | | | | | | | |
| Standardization of clinical practice | Guidelines, protocols, manuals; Cohort study | Leprosy, leishmaniasis, mpox, bacterial skin and soft tissue infections (cellulitis, erysipelas, rickettsioses, abscesses, and furuncles), superficial fungal infections (tinea infections and cutaneous candidiasis), parasitic infestations (scabies, pediculosis, tungiasis, myiasis, and cutaneous larva migrans), viral infections (herpes simplex and varicella), skin cancer (cutaneous melanoma, basal cell carcinoma, and squamous cell carcinoma), inflammatory and chronic dermatoses (contact dermatitis, acne, and psoriasis), and autoimmune diseases (systemic lupus erythematosus and pemphigus vulgaris) | **Argentina, Bolivia, Brazil, Chile, Colombia, Costa Rica, Cuba, Ecuador, El Salvador, Guatemala, Honduras, Mexico, Panama, Paraguay** General and at-risk populations, healthcare professionals \| NS \| Macro \| Health system | **Clinical outcomes:** Eczema severity, quality of life \| Improvement **Clinical outcomes:** Complete disease control, adherence \| Partial | Limited access to health services, specialists, and diagnostic tools  Insufficient training of healthcare personnel  Fragmentation of health services  Stigma and discrimination  Socioeconomic factors, inequality, and geographic barriers  Limitations in resources, funding, and treatment availability  Challenges related to treatment adherence and patient behaviors | Regulatory framework and institutional support  Funding and support from governmental entities and international organizations  Training of healthcare personnel and continuing professional education  Access to treatment and free or publicly funded programs  Patient education, communication, and adherence strategies  Scientific validation | Strengthen early diagnosis, screening, and active case detection  Provide continuous training for healthcare personnel and evaluators  Improve access, timely referral, and coordination across levels of care  Promote patient education, self-care, and community participation  Ensure the availability of resources, supplies, and technologies  Monitor indicators, outcomes, and quality of care  Promote adaptation to local contexts |
| **Social determinants of health** | | | | | | | |
| Conditional cash transfer programs | SR; cohort study; ecological study | Leprosy | **Brazil** Vulnerable populations \| NS \| Macro \| Community | **Clinical outcomes:** Adherence, cure, detection \| Improvement **Implementation:** Coverage \| Increase | NR | Improved household economic stability  Increased contact with health services | Integrate programs into leprosy control strategies  Cash transfers; registry system; state monitoring |
| Social housing programs | Cohort study | Leprosy | **Brazil** Vulnerable populations \| NS \| Macro \| Community | **Clinical outcomes:** Detection \| Increase | NR | NR | Investment in social housing  Comprehensive approach: integration with education, access to health services, and improvement of socioeconomic conditions |
| Participatory community programs | Qualitative | Leprosy | **Brazil** Population with leprosy \| NS \| Macro \| Community | **Experience:** Perceptions of healthcare professionals \| Social stigma and treatment refusal, gender inequalities, challenges in clinical management and follow-up, socioeconomic constraints, lack of resources (funding, diagnostics, self-care), and shortages of healthcare personnel and specialized services | NR | Community participation  Self-care groups  Patient associations  Local leadership | Stigma reduction and strengthening of regulatory frameworks  Gender equity and inclusion  Early detection and comprehensive management  Strengthening of the health system (quality, human resources for health, financing, and networks)  Humanized approach to care  Trained human resources; financing; infrastructure; specialized services; training |
| Policies on control, governance, decentralization, and surveillance | Narrative review | Leprosy | **Brazil** General population and health system \| NS \| Macro \| Community | **System:** elimination capacity \| Persistence of the disease associated with delayed diagnosis, stigma, case follow-up failures, weaknesses in surveillance systems, and the influence of social determinants | Regional inequalities  Institutional weaknesses  Inefficient decentralization  Limited resource availability  Disease relapses  Stigma | Existence of a structured health system  Recognition of leprosy as a social problem  Previous public policy trajectory | Strengthening of state and local capacities with a territorial and social determinants approach  Improved coordination  Strengthening of detection, diagnosis, follow-up, and reporting  Emphasis on continuous and comprehensive care  Promotion of community-based interventions integrated with disability prevention and rehabilitation |

LAC: Latin America and the Caribbean; NS: Not specified; TD: teledermatology; SLE; systemic lupus erythematosus; NR: Not reported; CHWs: community health workers; PHC: Primary health care; SR: Systematic review

**TABLE S6** Public policies that provide regulatory, technical and legal support to the identified tools, strategies and actions

| **Country** | **Policy** | **Objective** | **Pillar/Framework** | **Health condition** | **Year** | **Proposed level** |
| --- | --- | --- | --- | --- | --- | --- |
| Costa Rica | Plan Nacional para el Control del Cáncer de Costa Rica (2024-2030) | To reduce cancer mortality and improve survival through the implementation of effective interventions for comprehensive and timely cancer care, by establishing strategic partnerships, inter-institutional and intersectoral commitment, and the participation of social actors. | Work axis: comprehensive cancer approach | Cancer | 2024 | National |
| Cuba | Programa Integral para el Control del Cáncer (PICC) | To shift the clinical-epidemiological paradigm of the disease, making cancer a more curable condition through prevention, early diagnosis, and improved survival | NR | Cancer | 2010 (implementation); 2017 (document) | National |
| Argentina | Sistema de Riesgos del Trabajo (based on Law No. 24.557 and Decrees 658/96 and 49/14) | To reduce the incidence of occupational diseases through specific measures aimed at prevention and early detection | International Labour Organization (ILO) standards, national occupational risk legal framework, and joint management between public and specialized systems | Contact dermatitis (irritant and allergic) | 2018 | National level |
| Venezuela Brazil Guatemala | CD49.R19 - Plan de acción para la eliminación de enfermedades desatendidas y otras infecciones relacionadas con la pobreza | Implement a regional operational plan with specific elimination targets | Integration into health systems, monitoring, strengthening of national capacities | Neglected diseases | 2009 | Region of the Americas |
| Brazil | Políticas emergentes de controle de doenças infecciosas – Brasil (quadro integrado) | To prevent, early detect, control, and respond to the emergence or re-emergence of emerging and re-emerging infectious diseases, reducing their impact on public health through surveillance, rapid response, and integrated health system actions. | Early detection + integrated surveillance + rapid response + health system coordination | Emerging and re-emerging infectious diseases | 2011 | National |
| Argentina Brazil Uruguay Panama Costa Rica Colombia | Programas nacionales de inmunización | To prevent, control, and eliminate vaccine-preventable diseases through systematic, universal, and free vaccination of the population. | The program is based on:  Primary prevention in public health Equity in access to health services (universal and free vaccination) Immunization strategy as a public good  It is also guided by principles such as:  High population coverage Epidemiological surveillance Continuous updating of the immunization schedule | Vaccine-preventable diseases | 1999–2014 (First and second doses of varicella included) | National |
| Colombia | Plan National de Eliminación y Sostenimiento de la Eliminación de Enfermedades Transmisibles y Condiciones Prioritarias 2025-2031 | To contribute to interrupting transmission, achieving elimination as a public health problem, and sustaining the elimination of communicable diseases and priority conditions in Colombia. This will be achieved through the promotion of comprehensive health care with a participatory approach involving health system actors, other sectors, social and community stakeholders, and the general population; recognizing population and territorial realities to help reduce access barriers, health inequities, and social injustices, and to ensure the effective realization of the fundamental right to health for individuals, families, and communities across the national territory. | Action lines: i. Strengthening and integration of health systems and health service delivery ii. Strengthening strategic health information and surveillance systems iii. Addressing social and environmental determinants of health iv. Strengthening governance, stewardship, and financing | Vaccine-preventable diseases (e.g., measles), neglected infectious diseases (e.g., onchocerciasis), and other priority conditions (e.g., leprosy) | 2025-2031 | National |
| Venezuela Brazil Guatemala | CD55.R9 - Plan de acción para la prevención y el control de enfermedades transmisibles | To strengthen strategies for the prevention, control, and elimination of communicable diseases | Universal coverage, surveillance, integrated response, and health system strengthening | Communicable diseases (including NTDs) | 2016 | Region of the Americas |
| Latin America | Plan de acción para la eliminación de enfermedades transmisibles - Pan American Health Organization | To accelerate the elimination, control, or eradication of communicable diseases in the Region of the Americas, reducing health inequities through the strengthening of health systems, epidemiological surveillance, and universal access to effective interventions | Elimination + surveillance + health system integration + equity | Priority communicable diseases | 2008 | Region of the Americas |
| Region of the Americas | Iniciativa de la OPS para la eliminación de enfermedades: Política para aplicar un enfoque integrado y sostenible de las enfermedades transmisibles en la Región de las Américas | To provide a common and sustainable framework with prioritized lines of action to guide countries in the elimination of communicable diseases and other related problems by 2030 | 1) Strengthen integration of health systems and service delivery; 2) Strengthen strategic health information and surveillance systems; 3) Address social and environmental determinants of health; 4) Strengthen governance, stewardship, and financing | Prioritized communicable diseases (leishmaniasis, leprosy, onchocerciasis, measles, among others) and related conditions | 2019 | Regional – Region of the Americas (PAHO) |
| Panama | Plan Estratégico Nacional para la Eliminación de Enfermedades y Condiciones Relacionadas en la República de Panamá, 2025-2032 (PEET) | To contribute to the elimination of priority communicable diseases through integrated, sustainable, evidence-based, and people-centered strategies, with intersectoral and community participation | It is structured around four key strategic lines: Strengthening and integration of health systems and service delivery; Strengthening of surveillance and health information systems; Addressing social and environmental determinants; Strengthening governance, stewardship, and financing | Priority communicable diseases, leprosy, and vaccine-preventable diseases | 2025-2024 | National |
| Guatemala | Política sectorial de control de enfermedades transmitidas por vectores – Guatemala | To reduce morbidity and mortality associated with vector-borne diseases through integrated actions of prevention, epidemiological surveillance, vector control, and timely diagnosis and treatment | Integrated vector control approach based on continuous epidemiological surveillance, vector control (chemical, environmental, and community-based), strengthening of health services, and community and intersectoral participation | Vector-borne diseases | Leishmaniasis since 2003 | National |
| Brazil | Ending the neglect to attain the Sustainable Development Goals - A road map for neglected tropical diseases 2021–2030 - Overview (OMS) | To accelerate the control, elimination, and, where possible, eradication of neglected tropical diseases (NTDs) to contribute to the achievement of the Sustainable Development Goals (SDGs) by 2030, reducing disease burden, disability, and health inequities | Three global strategic pillars: accelerating programmatic actions; strengthening cross-cutting approaches; transforming operational models with country leadership | Neglected tropical diseases (NTDs) | 2020 | International/Global |
| Latin America | Resolución WHA 60.13 (2007) - World Health Assembly - WHO | To strengthen the prevention, control, and elimination of neglected tropical diseases (NTDs) through the development of comprehensive strategies including epidemiological surveillance, access to diagnosis and treatment, and international coordination. | Integrated NTD control approach based on strengthening health systems, expanding equitable access to effective interventions, improving surveillance and health information generation, and promoting international cooperation and partnerships. | Neglected tropical diseases (NTDs) | 2007 | Global |
| Venezuela Brazil Guatemala | CD48.R12 - Eliminación de enfermedades desatendidas y otras infecciones relacionadas con la pobreza | To promote the elimination of neglected diseases as a public health problem | Comprehensive approach: access to services, cost-effective interventions, and reduction of inequities | Neglected tropical diseases (including onchocerciasis, leprosy, etc.) | 2008 | Region of the Americas |
| Brazil | Estratégia Global da OMS para Doenças Tropicais Negligenciadas (DTNs) - Resolução WHA66.12 (2013). | To prevent, control, eliminate, and where possible eradicate neglected tropical diseases (NTDs), reducing their burden in vulnerable populations and contributing to the achievement of the Sustainable Development Goals (SDGs) | The strategy is based on integrated public health approaches: integrated and cost-effective interventions; preventive chemotherapy (MDA); intensified case management; strengthening of epidemiological surveillance; integration into national health systems; equity-focused approach and attention to vulnerable populations; intersectoral action (water, sanitation, education); and community participation | Neglected tropical diseases (NTDs) | Initial global plan: 2008–2015; updated framework: 2021–2030 | Global |
| Venezuela Brazil Guatemala | WHO NTD Roadmap 2021–2030 (Hoja de ruta de la OMS para las enfermedades tropicales desatendidas 2021–2030) | Global policy framework guiding the prevention, control, elimination, and where possible eradication of 20 neglected diseases and disease groups. It replaces and updates the first roadmap from 2012, aligning with the Sustainable Development Goals and the principle of “leaving no one behind” | Programmatic action, cross-cutting approaches, and operational model transformation | Neglected tropical diseases and disease groups (20 conditions) | Endorsed by the 73rd World Health Assembly in 2020 | Region of the Americas |
| Ecuador | Estrategia Nacional de Inmunizaciones (ENI) | To ensure free, equitable, and universal immunization for disease prevention | Core principles: equity, universality, free provision, and quality across all points of the public health system. Coverage and scope: the National Immunization Program aims to maintain coverage above 95% for vaccine-preventable diseases. | Immunization schedule: includes vaccines for tuberculosis, hepatitis B, rotavirus, polio, pentavalent vaccine, pneumococcal disease, measles, rubella, yellow fever, and HPV, prioritizing children, pregnant women, and older adults | 2017 | National |
| Brasil | Nota técnica – Red de laboratorios de leishmaniasis (Minas Gerais, Brasil) | To organize and strengthen the public laboratory network for leishmaniasis diagnosis, ensuring timely access, diagnostic quality, and coordination across levels of the health system. | Hierarchical diagnostic service network organization based on: decentralization of initial diagnosis (local/regional levels), centralization of confirmatory and high-complexity testing (state level), definition of referral and counter-referral flows, and standardization of diagnostic techniques (e.g., DPE, PCR) | Cutaneous leishmaniasis | 2017 | Sub-national |
| Argentina | Ley 22.964 Normas a fin de controlar la lepra en el país | To control the endemicity of leprosy in the country, the provisions of this law shall be implemented throughout the national territory. Each province, the Municipality of the City of Buenos Aires, and the National Territory of Tierra del Fuego, Antarctica, and the South Atlantic Islands shall additionally enact complementary regulatory measures | Mandatory nationwide implementation under the leadership of the national health authority in coordination with provincial and local jurisdictions Free provision and compulsory care: state guarantee of free treatment in official health facilities and the obligation of patients to comply with medical follow-up Technical and scientific updating: development of norms based on scientific progress to reduce the infectious period and avoid unnecessary isolation of patients | Leprosy | 1893 | National |
| México | Norma Oficial Mexicana NOM-027-SSA2-2007 | To establish criteria for developing procedures for the prevention and control of leprosy | Health promotion: focused on health education, social participation, and health communication to inform the population and reduce stigma and misconceptions about the disease.  Control activities: include case identification and diagnosis, treatment with multidrug therapy (MDT), supervision of medication adherence, and post-treatment surveillance.  Rehabilitation: procedures aimed at restoring physical, mental, and social functions, as well as preventing disabilities through self-care and assistive devices.  Epidemiological surveillance: includes case reporting, contact tracing, outbreak investigation, and follow-up through the National Laboratory Network | Leprosy | 2007 | National |
| Brasil | Directrices nacionales de vigilancia de lepra – Brasil (2010) - Ordenanza Administrativa (Portaria) n.º 3.125/2010 | To standardize and strengthen epidemiological surveillance actions, early diagnosis, timely treatment, and follow-up of leprosy cases and contacts, in order to reduce transmission, prevent disabilities, and progress toward its elimination as a public health problem | Early detection + contact surveillance + free treatment + primary health care integration | Leprosy (Hansen’s disease) | 2010 | National |
| Brasil | Estrategia mundial de lepra – OMS | To reduce the global burden of leprosy by interrupting transmission, ensuring early case detection, and reducing associated disability, with the aim of advancing toward its elimination as a public health problem | Detection + prevention of transmission + comprehensive management + social and rights-based approach | Leprosy (Hansen’s disease) | 2016 | Global |
| Colombia | Plan Estratégico de Colombia para el control de la lepra 2010–2015 | To reduce the burden of leprosy in Colombia through early detection, timely treatment, and reduction of transmission, with the aim of advancing toward its elimination as a public health problem | Se fundamenta en el fortalecimiento de It is based on strengthening surveillance and integrated control within the health system, with emphasis on: early diagnosis in primary care; multidrug therapy (MDT); active epidemiological surveillance; contact tracing and follow-up; disability prevention; and alignment with international guidelines | Leprosy (Hansen’s disease) | 2010-2015 | National |
| Brasil | Portaria Conjunta nº 125/2009 - It reflects the decentralization process toward primary health care  Estrategia Nacional 2024–2030 - It is the current national strategic policy | To interrupt leprosy transmission and reduce its burden as a public health problem through early diagnosis, timely treatment, contact surveillance, and reduction of disabilities and stigma | It is based on the decentralization of actions toward primary health care, integrated within the Unified Health System (SUS), with emphasis on: universal access, early detection, free treatment, epidemiological surveillance, and service integration | Leprosy (Hansen’s disease) | 2009 | National |
| Uruguay | Decreto Nº 41/012  se aprobó la actualización del Código Nacional sobre Enfermedades y Eventos Sanitarios de  Notificación Obligatoria. | To define the list of diseases and events considered subject to mandatory notification, as well as reporting deadlines, responsible parties, and sanctions established in case of non-compliance | NR | Leprosy, leishmaniasis, measles, varicella, smallpox | NR | National |
| El Salvador | Acuerdo No. 1319 "Política Nacional para la prevención y control del cáncer" | To establish the interventions required for cancer prevention and control in order to reduce the burden of disease in the Salvadoran population and improve quality of life, through health promotion, prevention, early detection and diagnosis, timely treatment, rehabilitation, palliative care, and research. | 1) Promote health to encourage healthy lifestyles and specific cancer prevention activities in the Salvadoran population; 2) Develop and integrate early detection interventions for priority cancers within the National Health System; 3) Strengthen institutional capacities for early diagnosis, timely treatment, rehabilitation, and palliative care across all levels of the RIISS to ensure equitable, comprehensive, and sustainable service delivery; 4) Strengthen institutional capacities for the creation and consolidation of a National Cancer Registry to implement nationwide cancer surveillance | Neoplasms | 2015 | National |
| Mexico | Programa Institucional 2025-2030 del Instituto Nacional de Cancerología | To achieve overall population well-being through universal and free access to oncological services, strengthening specialist training, and generating scientific knowledge for timely diagnosis and treatment | 1) Contribute to universal and free access to health services (medical and hospital care, diagnostic tests) and medications for people with any cancer condition at the tertiary care level; 2) Strengthen training and development of human capital for addressing health problems in Mexico in the field of neoplasms; 3) Contribute to the generation of oncological scientific knowledge by the Institute to support timely cancer diagnosis and treatment in the Mexican population | Neoplasms | 2025-2030 | National |
| Venezuela Brasil Guatemala | CD35.R14 - Control de la oncocercosis en las Américas | To establish regional actions for the control of onchocerciasis | Epidemiological surveillance, mass treatment (ivermectin), regional cooperation | Onchocerciasis | 1991 | Región de las Américas |
| Mexico | Programa de Acción Específico (PAE) Eliminación de la Oncocercosis 2013–2018 | To eliminate onchocerciasis as a public health problem in Mexico through treatment, surveillance, and control actions in endemic areas | The program is based on the prevention, control, and elimination of neglected communicable diseases, with strategies including mass drug administration (ivermectin), epidemiological surveillance, and post-elimination monitoring. Additionally, as with all Priority Action Programs (PAEs) in Mexico, it is aligned with the 2013–2018 Sectoral Health Program, which in turn is aligned with the 2013–2018 National Development Plan | Onchocerciasis | 2013-2018 | National |
| Argentina | Resolución 4217/2023 Inmunización contra VPH | To reduce the incidence and mortality of cervical cancer (CC), decrease the burden of disease associated with HPV and its complications, and contribute to gender equity | Free and mandatory HPV immunization integrated into the National Immunization Schedule; strategic simplification through a single-dose regimen to improve coverage and implementation efficiency; scientific evidence supporting the immunogenicity, efficacy, and safety of simplified schedules; gender equity through mandatory inclusion of both girls and boys at 11 years of age; and optimization of human, financial, and environmental resources to facilitate the integration of health strategies | HPV | 2023 | National |
| Argentina | Estrategia mundial para acelerar la eliminación del cáncer de cuello uterino como problema de salud pública. | To achieve a world in which cervical cancer is eliminated as a public health problem, reaching an incidence threshold below 4 per 100,000 women per year in all countries . | Primary prevention through HPV vaccination (90% of girls fully vaccinated by age 15); secondary prevention through screening and treatment of precancerous lesions (70% of women screened with a high-performance test at ages 35 and 45); tertiary prevention through treatment and care of invasive cancer and palliative care (90% of women with cervical disease treated); strengthening of health system enablers (human resources, access to medicines, digital technologies); partnerships, advocacy, and multisectoral communication; and surveillance, monitoring, and impact evaluation | HPV | 2020 | Mundial |
| Panama | Política Nacional de Salud 2016-2025 (MINSA) | To improve population health through strengthening health governance, health promotion, surveillance of determinants, and social participation to achieve universal access and universal health coverage | Axis I: Protecting the population through regulation and stewardship of the health system; Axis II: Improving the quality and access of health services for the population; Axis III: Promoting health through social participation | Comprehensive population health and health determinants (communicable and non-communicable diseases, environmental and social risks) | 2016-2025 | National |
| Perú | Política Nacional Multisectorial de Salud al 2030 "Perú, País Saludable" | To achieve a 5% reduction in healthy life years lost due to preventable causes by 2030, thereby decreasing premature mortality and years lived with disability . | The policy is structured around three priority objectives (POs) that serve as its strategic pillars: PO1: Improvement of healthy habits and lifestyles, aimed at transforming individual and family behaviors through health education and the creation of environments that facilitate healthy practices; PO2: Universal access to comprehensive services, focused on ensuring timely, continuous, and high-quality care while reducing health system fragmentation; PO3: Improvement of living conditions, addressing the social determinants of health (water, sanitation, road and environmental safety) to reduce population risk and vulnerability | Comprehensive health and social determinants (non-communicable diseases, communicable diseases, and injuries) | 2020-2030 | National |
| El Salvador | Decreto Nº 955 Código de Salud | To ensure adequate immunization of the population against vaccine-preventable infectious diseases through the regulation and promotion of mechanisms that guarantee access within the national health system | Free access and equity: guaranteed free administration without discrimination across the entire system, including the private sector when vaccines are provided by the State; Mandatory vaccination: immunization is compulsory as determined by the governing health authority to protect public health; Financial sustainability: establishment of a special fund of no less than 1% of the previous fiscal year's national budget; Quality and safety: use of internationally certified vaccines and strict maintenance of the cold chain | No distinction | 2012 | National |
| Honduras | Modelo Nacional de Salud | To contribute to the establishment of a plural, integrated, and properly regulated national health system, focused on the equitable improvement of the health status of individuals, families, and communities within their environment | 1. Service delivery/provision: operational axis focused on population needs; 2. Management: framework for planning and governing services under a decentralization and results-based approach; 3. Financing: ensuring solidarity-based and sustainable resources to guarantee equitable access | No distinction | 2013 | National |
| El Salvador | Norma Técnica para la Prevención y Control de las Infecciones de Transmisión Sexual | To establish technical criteria and procedures for the promotion, prevention, care, and control of sexually transmitted infections (STIs), within the field of sexual and reproductive health, with social participation and intersectoral collaboration | Sexual and reproductive health perspective; gender approach; rights-based approach; people-centered care approach; health promotion | Sexually transmitted infections (STIs) | 2012 | National |
| Paraguay | Política Nacional de Salud 2015-2030 | To advance toward universal access to health and achieve the highest possible level of universal health coverage in order to reduce health inequities and improve population quality of life within the framework of sustainable human development | The policy is based on four fundamental values that guide its implementation: Right to health: guarantee of the highest attainable standard of health as a fundamental right without distinctions; Equity: absence of unfair differences in access and health status; Solidarity: collective action for the common good and social protection; Respect for diversity: recognition of differences as a positive contribution | No distinction | 2015 | National |
| Mexico | Programa Sectorial de Salud 2025-2030. | To consolidate the country’s transformation under a development model based on well-being, social justice, and sustainability, ensuring that every person in the national territory has access to free, comprehensive, and high-quality health services | Foundational pillars: 1. Universal access: ensuring access to health services for the entire population; 2. Resolutive capacity: improving the quality and effectiveness of care within the National Health System; 3. Supplies and medicines: guaranteeing timely and free provision of medicines; 4. Promotion and prevention: improving population health and preventing avoidable diseases; 5. Integration and modernization: strengthening governance and digitalization of the health system | No distinction | 2025-2030 | National |
| Brazil | Atenção Primária à Saúde (APS) no Sistema Único de Saúde | To ensure universal, equitable, and comprehensive access to health services through a care model focused on promotion, prevention, diagnosis, treatment, and rehabilitation from the primary level of care | Universal access + comprehensive care + territorial organization + continuity of care | No distinction | 1990´s | National |
| Honduras | Código de Salud, Decreto Número 65-91 | To safeguard the right of every person to enjoy, maintain, and recover their health (understood as comprehensive well-being: biological, psychological, social, and ecological), regulating its promotion, protection, recovery, and rehabilitation | 1. Health as a human right: State obligation and individual responsibility to promote protection and integral well-being; 2. Governance and standard-setting: authority of the Ministry of Health to define national policy, planning, and sector coordination; 3. Decentralization: action at departmental and municipal levels under principles of administrative coordination; 4. Intersectoral coordination: functional integration of various ministries (Health, Labor, Education, etc.) and autonomous institutions such as IHSS; 5. Social responsibility: individuals’ duty to contribute to community health and environmental protection | No distinction | 1996 | National |
| Colombia | Decreto 903 de 2014 Por el cual se dictan disposiciones en relación con el Sistema Único de Acreditación en Salud | El The purpose of this decree is to establish provisions and adjustments to the Single Health Accreditation System as a component of the Mandatory Quality Assurance System for Health Care, as well as to define rules for its operation within the General Systems of Social Security in Health and Occupational Risks | **Principles**: 4.1 Graduality: the level of compliance required by the standards of the approved accreditation manuals will progressively increase over time, aiming to promote continuous improvement in the quality of health care; 4.2 Information management: information generated during the accreditation process will be subject to the conditions established between the accrediting entity and the entities undergoing accreditation; 4.3 Comprehensiveness: accreditation can only be granted when all processes of the applicant entity comply with the applicable accreditation standards, according to its institutional scope | No distinction | 2014 | National |
| Perú | Decreto Legislativo N° 1504, Decreto Legislativo que fortalece al Instituto Nacional de Salud para la prevencibn y control de las enfermedades, | To establish measures that strengthen the National Institute of Health and the stewardship of the Ministry of Health for the prevention and control of diseases, in order to improve public health, promote population well-being, and contribute to sustainable development | The purposes of this Legislative Decree are: a) Strengthen the National Health System to ensure intra- and intersectoral, intergovernmental articulation among public and private entities and the population for the implementation of health policies; b) Strengthen the health sector through the organization and efficient management of the National Institute of Health in support of public health; c) Improve surveillance and prevention of diseases, outbreaks, endemic, epidemic, and pandemic events nationwide; d) Contribute to the control of diseases affecting population health; e) Improve research, development, innovation, and health technologies with participation from public, private, and academic institutions | No distinction | 2020 | National |
| El Salvador | Decreto Nº 1013 Ley de Vacunas | To ensure adequate immunization of the population against vaccine-preventable infectious diseases through the regulation and promotion of mechanisms within the national health system | Free access and equity: guaranteed free administration without discrimination across the entire system, including the private sector when the State supplies the vaccine; Mandatory vaccination: immunization is compulsory as determined by the governing authority to protect public health; Financial sustainability: establishment of a special fund of no less than 1% of the previous fiscal year's national budget; Quality and safety: use of internationally certified vaccines and strict maintenance of the cold chain | No distinction | 2012 | National |
| El Salvador | Decreto Nº 955 Código De Salud | To develop the constitutional principles related to public health and social assistance of the inhabitants of the Republic, as well as to establish the rules for the organization, functioning, and powers of health authorities and their relationship with private services | To define national health policy, control and supervise its implementation; ensure the essentially technical nature of public health services and the oversight of health professions; establish mandatory coordination and collaboration among state, autonomous, and private entities for the fulfillment of health objectives; and promote a comprehensive approach based on health promotion, protection, recovery, and rehabilitation | No distinction | 1988 | National |
| Honduras | Decreto No. 288-2013 Ley De Vacunas De La República De Honduras | To establish the regulatory framework applicable to the organization and functioning of the National Vaccination Scheme as a preventive and health protection instrument through the application of safe, effective, high-quality vaccines with easy distribution and access for both national and foreign populations in the country | Financing: creation of a special fund for vaccines; Mandatory nature: vaccination as a duty of both citizens and the State; Governance: central role of the Ministry of Health in the National Immunization Program | No distinction | 2013 | National |
| Guatemala | Decreto Número 25-2024 Ley De Vacunación | To guarantee protection for the general population against vaccine-preventable diseases through universal administration and targeted vaccination of at-risk groups, using high-quality, safe, and effective vaccines | 1. Universality and free access: universal and cost-free access within public health services; 2. Equity and equality: non-discriminatory access with prioritization of vulnerable groups; 3. Availability: assurance of timely supply of vaccines and related inputs; 4. Voluntariness and freedom: vaccination is voluntary and not a requirement for employment or education | No distinction | 2024 | National |
| Guatemala | Decreto Número 7-2024 Ley Para La Atención Integral del Cáncer | To organize the State’s response actions to strengthen public policies and interventions that ensure comprehensive care for reducing cancer morbidity and mortality in Guatemala | 1. Universality: health as a universal and inalienable human right; 2. Free provision: fully free comprehensive care within public health services; 3. Accessibility: guarantee of services without physical, economic, geographic, or sociocultural barriers; 4. Sustainability: State obligation to ensure lasting financial, human, and technological resources; 5. Quality: care based on scientific evidence and humanized care | No distinction | 2024 | National |
| Guatemala | Decreto Número 90- 97 Código de Salud | To ensure the most complete physical, mental, and social well-being of the population, guaranteeing the right to health through actions of prevention, promotion, recovery, and rehabilitation without any discrimination | It is based on five guiding principles: 1. Health as a human right and public good: the State must guarantee life and comprehensive development, recognizing health as an inalienable right; 2. National stewardship: the Ministry of Health exercises leadership, regulation, oversight, and evaluation of all health sector institutions; 3. Decentralization and deconcentration: organization of services based on population needs and administrative modernization; 4. Social participation: guarantee of community participation in planning, organization, and oversight of health services; 5. Equity, solidarity, and subsidiarity: foundational principles for policy design and resource allocation, prioritizing the most underserved populations | No distinction | 1997 | National |
| Chile | Estrategia de Salud Digital como política pública que enmarca el Programa Nacional de Telesalud 2018 y el programa operativo Hospital Digital | To improve access, timeliness, and quality of health care through the use of digital technologies, enabling the provision of remote services across the entire continuum of care (promotion, prevention, diagnosis, treatment, rehabilitation, and palliative care) | It is based on: use of information and communication technologies (ICT) in health; equitable and universal access to health services; people-centered care; and integration within health care networks | No distinction | 2017-2018 | National |
| Peru | La Ley N° 28010, Ley General de Vacunas | To ensure adequate immunization of the Peruvian population against vaccine-preventable diseases by establishing a legal framework that guarantees the procurement, financing, and free administration of vaccines | Free and mandatory access: free and compulsory access to vaccines under the National Immunization Schedule for all inhabitants of the country; Protected financing: resources allocated to vaccine procurement are priority and earmarked, and cannot be diverted to other purposes; Technical stewardship: the Ministry of Health is the governing authority responsible for defining the national schedule, technical standards, and supervision of coverage targets; Universality and equity: guaranteed immunization without discrimination throughout the national territory, integrating both public and private sectors in achieving objectives | No distinction | 2003 | National |
| Argentina | Ley 27491 Control de enfermedades prevenibles por vacunación | To regulate the implementation of a public policy for the control of vaccine-preventable diseases | Free vaccination and equitable access to vaccination services across all stages of life; mandatory vaccination for all inhabitants; primacy of public health over individual interest; availability of vaccines and vaccination services; and participation of all health sectors and other actors linked to social determinants to achieve adequate coverage levels | No distinction | 2019 | National |
| Nicaragua | Ley N°. 423, Ley General de Salud | To safeguard the right of every person to enjoy, maintain, and recover their health, regulating the principles, rights and obligations, as well as actions for health promotion, prevention, recovery, and rehabilitation | The law is based on the following fundamental principles (Art. 5): 1. Free provision: guaranteed for vulnerable groups (mother-child, older adults, persons with disabilities); 2. Universality: health service coverage for the entire population; 3. Solidarity: access to essential services through contribution and resource redistribution; 4. Comprehensiveness: integrated actions of prevention, promotion, treatment, and rehabilitation; 5. Equity: access opportunities prioritizing vulnerable populations | No distinction | 2002 | National |
| Ecuador | Ley orgánica de salud | To regulate actions that enable the effective realization of the universal right to health enshrined in the Political Constitution of the Republic and the law | Equity and comprehensiveness; solidarity and universality; non-waivability and indivisibility; participation and plurality; quality and efficiency | No distinction | 2025 | nacional |
| Mexico | Modelo de Atención en Salud – Sistema Nacional de Salud de México | To guarantee effective, equitable, and continuous access to health services for the entire population through a comprehensive model that prioritizes health promotion, disease prevention, timely care, and continuity of care, especially for vulnerable populations | Primary Health Care (PHC) + equity + preventive approach + territorial organization of the health system | No distinction | 1980-1990 | National |
| Mexico | Norma Oficial Mexicana NOM-017-SSA2-2012, Para la vigilancia epidemiológica. | To establish the criteria, specifications, and operational guidelines of the National Epidemiological Surveillance System (SINAVE) for the systematic, timely, and reliable collection of relevant health information | Morbidity surveillance: covers the registration and reporting of communicable and non-communicable diseases, emerging and re-emerging diseases, and malignant neoplasms; Mortality surveillance: includes mandatory reporting of all conditions defined in the current International Classification of Diseases (ICD); Special surveillance: applies to conditions or potential risks that, due to their magnitude or relevance, require additional information and specific monitoring mechanisms; Public health emergency surveillance: focuses on new or re-emerging events that pose an immediate risk to population health; International surveillance: coordinated by the General Directorate of Epidemiology as the National Focal Point, in accordance with the International Health Regulations | No distinction | 2013 | National |
| Ecuador | Plan Decenal de Salud 2022-2031 (PDS) | To improve the health status and quality of life of the country’s population and to make the right to health effective through a comprehensive approach to its determinants | Health equity: reducing inequalities in access to universal and free services through the addressing of social and environmental determinants; health promotion: fostering well-being and healthy lifestyles across all stages of life; preventive medicine: reducing the burden of disease and preventable deaths; timely and quality care: ensuring comprehensive services with an emphasis on primary care; integrated and efficient health system: transforming the National Health System to ensure universal access and coverage | No distinction | 2022-2030 | National |
| Colombia | Plan Decenal Salud Pública 2022-2031 | To generate a strategy that ensures the integrality and sustainability of public health management by strengthening participatory mechanisms and technical and human capacities, thereby consolidating comprehensive health planning through the articulation of actors and sectors at national and territorial levels and strengthening health authority | 1. Governance and stewardship of public health; 2. Indigenous peoples and communities, rural populations, women, LGBTIQ+ sectors, and other populations by condition and/or situation; 3. Social determinants of health; 4. Primary Health Care (PHC); 5. Climate change, emergencies, disasters, and pandemics; 6. Public health knowledge and health sovereignty; 7. Health workforce | No distinction | 14 de junio de 2022 | National |
| Brazil | Política brasileira de telessaúde (Programa Telessaúde Brasil Networks – Brasil. Ministério da Saúde. Portaria GM/MS nº 2.546/2011) | To connect primary health care (PHC) with specialists through digital tools such as teleconsultation, tele-education, and tele-diagnosis | It is structured on the pillars of the Telessaúde Brasil Redes program: teleconsultation, tele-education (continuing professional training), tele-diagnosis, and formative second opinion. Its general foundation is the technological integration of the public health system | No distinction | 2007 | National |
| Colombia | Política de Atención Integral en Salud (articulado con Decreto 903 de 2014) | To orient the health system toward generating the best possible health conditions for the population through the regulation of the conditions under which actors intervene, ensuring access to health services that are timely, effective, and of quality for the preservation, improvement, and promotion of health, thereby guaranteeing the right to health, in accordance with “Ley 1751 de 2015, Estatutaria de Salud”. | Strategies: 1. Primary Health Care (PHC) as the core strategy, integrating population needs, system response, and available resources, with a family- and community-health approach that strengthens interdisciplinary teams and capacities; 2. Care as a shared responsibility between individuals, families, communities, the State, and the environment, emphasizing self-care and collective responsibility for health protection; 3. Comprehensive risk management in health, integrating public health, insurance, and service delivery to minimize risk and manage disease; 4. Differential approach in care, adapting the model to territorial, population, and service structure characteristics as key success factors of the health system | No distinction | enero 1 de 2016 | National |
| Global | Política global OMS: vacunación contra varicela en inmunización rutinaria | To reduce the population burden of varicella disease through the implementation of routine vaccination in countries with adequate epidemiological conditions and coverage levels, aiming to prevent cases, severe complications, and outbreaks, and to decrease associated morbidity | Universal childhood immunization strategy; health equity and primary prevention principle; epidemiological evidence on disease burden and vaccine effectiveness; critical implementation condition of sustained high coverage (≥80%) to avoid age shift in infection; integration into National Immunization Programs (NIP) | No distinction | 2000 | Global |
| Brazil | Política Nacional de Assistência Farmacêutica (PNAF) - (decretos 2.577/2006 e 204/2007) | To guarantee equitable, comprehensive, and rational access to essential medicines as part of the right to health, contributing to comprehensive care within the Unified Health System (SUS) | It is based on: the constitutional right to health in Brazil; universal and equitable access to medicines; rational use of medicines; and comprehensiveness of health care | No distinction | 2004 | National |
| Brazil | Política Nacional de Atenção Básica (PNAB) como política pública que estabelece a APS no Brasil. | To organize and strengthen Primary Health Care (PHC) as the entry point and coordinating axis of the health system, ensuring universal access, comprehensiveness of care, and coordination of care within the Unified Health System (SUS) | It is based on: the universal right to health; equitable and territorialized access; comprehensiveness of care; care coordination from PHC; and a family- and community-based approach. It also relies on organizational principles such as population registration (adscription), multiprofessional team-based work, and continuity of care over time | No distinction | 2006 | National |
| Bolivia | Política Nacional de Calidad en Salud (RM: Nº 0414 de 11 de julio de 2023) | To systematically and permanently ensure equity and quality in the care provided to users of health services and internal users through the implementation of a Quality Management System and the development of a culture of quality, emphasizing respect for rights and dignified treatment of individuals, families, and communities | 1. Evaluation and updating of PRONACS and regulatory frameworks; 2. Patient safety; 3. Continuous quality improvement and health auditing; 4. Health quality surveillance | No distinction | 11 de julio de 2023 | National |
| Costa Rica | Política Nacional de Salud 2023-2030 | To improve the health conditions of the population through addressing the social determinants of health, universal access and coverage, digital health, research and technological innovation, health surveillance, environmental health, and health promotion | Axis: 1. Quality, access, and coverage; 2. Research and knowledge management; 3. Health surveillance; 4. Innovation, digital health, and health technologies; 5. Environmental health; 6. Health promotion | No distinction | 2023 | National |
| Brazil | Política Nacional de Vigilância em Saúde - Sistema Nacional de Notificação de Doenças (SINAN) - inclui o Programa Nacional de Controle da Leishmaniose | To organize and strengthen health surveillance actions throughout the national territory in order to identify health risks and damages, prevent and control diseases and other public health events, and guide decision-making and timely response within the health system | It is based on an integrated surveillance model that articulates epidemiological surveillance, sanitary surveillance, environmental surveillance, and occupational health surveillance | No distinction | 2000 | National |
| Brazil | Políticas públicas de proteção social no Brasil, expressas principalmente por meio do Programa Bolsa Família e do Sistema de Assistência Social. | To reduce poverty and social inequality through conditional cash transfers, guaranteeing a minimum income for vulnerable households and promoting access to basic health, education, and nutrition services | Conditional cash transfers (CCTs); rights-based social protection approach; conditionalities including school attendance for children and adolescents, health check-ups (vaccination, prenatal care, child growth monitoring); and intersectoral integration between health, education, and social assistance sectors | No distinction | 2004 | National |
| Brazil | Programa “Minha Casa Minha Vida” (MCMV) do Brasil | To reduce the housing deficit in Brazil and improve living conditions for the population, especially low-income households, through access to adequate, safe housing with basic services | It is based on a social policy approach focused on structural determinants: access to adequate housing and housing subsidies; reduction of poverty and social inequality; improvement of habitability conditions (space, materials, sanitation); articulation with urban infrastructure and basic services; and prioritization of vulnerable populations | No distinction | 2009 | National |
| Colombia | Sistema Obligatorio de Garantía de la Calidad en Salud (SOGCS), regulado por el Decreto 1011 de 2006 | To ensure the quality of health care in Colombia through the implementation of a mandatory system that improves patient safety, accessibility, timeliness, appropriateness, and continuity of health services | Regulation, continuous improvement, measurement, and accreditation | No distinction | 2006 | National |
| Bolivia | Política de la Salud Familiar Comunitaria Intercultural (SAFCI) | To contribute to the elimination of health-related social exclusion; to strengthen and deepen effective social participation in health decision-making; to provide health services that consider individuals, families, and communities; and to recognize, respect, value, and integrate biomedical medicine with the traditional medicine of indigenous and rural peoples, thereby improving the population’s living conditions | The SAFCI policy is implemented through two components: (1) the Family, Community, and Intercultural Health Care Model; and (2) the Participatory Management and Social Control Model in Health | No distinction | 01 de abril de 2013 | National |
| Brazil | Política Nacional de Gestão de Tecnologias em Saúde | To maximize health benefits achievable with available resources, ensuring population access to effective and safe technologies under equitable conditions | 4.1 Use of scientific evidence to support decision-making through Health Technology Assessment; 4.2 Improvement of technology incorporation processes; 4.3 Rationalization of technology use; 4.4 Support for strengthening education and research in health technology management; 4.5 Systematization and dissemination of information; 4.6 Strengthening of governmental structures; 4.7 Political-institutional and intersectoral coordination | No distinction | 2010 | National |
| Argentina | Ley 15465 Enfermedades Infecciosas | To ensure the protection of public health through mandatory reporting of cases and carriers of infectious diseases, enabling the immediate adoption of isolation, prevention, and sanitary control measures | Mandatory nationwide reporting of both suspected and confirmed cases as well as carriers of pathogens; strategic classification of diseases into groups (A, B, C, and D) to determine reporting deadlines and response urgency; professional and technical responsibility involving physicians, veterinarians, dentists, laboratory staff, and auxiliary personnel within the surveillance system | No distinction | 1960 | National |

**Glossary**

**Clinical Practice Guideline:** Clinical practice guidelines are statements that include recommendations intended to optimize patient care and are informed by a systematic review of the evidence and an assessment of the benefits and harms of alternative treatment options (1).

**Health Policy:** According to the World Health Organization (WHO), health policy is defined as “the decisions, plans, and actions (and omissions) undertaken to achieve specific health care goals within a society or undertaken by a set of institutions and organizations at the national, state, and local levels to promote public health” (2).

**Health Program:** A program is a set of activities aimed at addressing specific types of health problems and health-related aspirations of a population within its environment. It includes the specification of the problems to be addressed, the resources to be used, and the manner in which these resources are to be organized and managed (3).

**Policy-Process:** The sequence of activities that occur to move a proposed solution to an identified problem from the government agenda to formulation to implementation to modification (4).

**References**

1.	Institute of Medicine (U.S.), Graham R, editors. Clinical practice guidelines we can trust. Washington, DC: National Academies Press; 2011. 266 p.

2.	O’Brien GL, Sinnott SJ, Walshe V, Mulcahy M, Byrne S. Health policy triangle framework: Narrative review of the recent literature. Health Policy OPEN. 2020 Dec;1:100016. doi:10.1016/j.hpopen.2020.100016

3.	Montoya-Aguilar C. Los programas y la programación de la atención de salud. CMS. 2005 Sep 26;45(3):224–31.

4.	Kreidler, M. L. Health care policy and politics. [Internet]. 2009. Available from: https://www.ebsco.com/research-starters/consumer-health/health-care-policy-and-politics/
